# Involving Sales Managers in Sales Force Compensation Design

**DOI:** 10.1177/0022243720969174

**Published:** 2020-12-14

**Authors:** Rob Waiser

**Keywords:** agency theory, asymmetric information, delegation, sales force compensation, sales manager

## Abstract

Sales force incentive design often involves significant participation by sales managers in designing the compensation plans of salespeople who report to them. Although sales managers hold valuable territory-level information, they may benefit from misrepresenting that information given their own incentives. The author uses a game theoretic model to show (1) how a firm can efficiently leverage a manager’s true knowledge and (2) the conditions under which involving the manager is optimal. Under the proposed approach, the firm delegates sales incentive decisions to the manager within restrictive constraints. She can then request relaxed constraints by fulfilling certain requirements. The author shows how these constraints and requirements can be set to ensure the firm’s best possible outcome given the manager’s information. Thus, this “request mechanism” offers an efficient, reliable alternative to approaches often used in practice to incorporate managerial input, such as internal negotiations and behind-the-scenes lobbying. The author then identifies the conditions under which this mechanism outperforms the well-established theoretical approach of offering the salesperson a menu of contracts to reveal territory-level information.

For over 30 years, the dominant paradigm in sales force incentive compensation theory has been the principal–agent model. The standard application to sales, first offered by Basu et al. (1985), involves a firm (principal) seeking to design an optimal compensation plan for a salesperson (agent) without being able to observe the salesperson’s selling effort. This two-player model provides valuable insights and has spawned numerous extensions. However, its treatment of the firm as a single decision-making entity masks the interesting and important role commonly played by sales managers.

For example, I worked with a firm (Firm A) that decided to use commission-based incentives for a sales team selling a new product. Because the product’s potential was not evenly distributed among the sales territories due to geographic and other constraints, Firm A decided, in the interest of fairness and motivation, to set three commission rates. Salespeople in the most difficult territories would receive the highest rate and those in the easiest territories would receive the lowest. When determining which rate each territory should receive, the firm asked for input from its first-line sales managers. However, those managers also earned commissions for sales in their districts, so they stood to gain from maximizing their salespeople’s incentives. The result was a lengthy internal negotiation process with every manager arguing for the difficulty of her^1^ territory and Firm A trying to distinguish truth from misrepresentation.

More recently, I spoke with a manager from Firm B, which uses quota-based sales incentives. He described the firm’s top-down quota-setting process, in which a global sales target is allocated to individual countries and then to progressively lower levels (e.g., from nation to regions to districts to territories). Sales managers provide input into allocations at the level below their own. For example, a district manager participates in allocating the target for her district to the territories she manages. The manager I spoke with complained of extensive “lobbying” by salespeople and managers at each level to convince their supervisors to assign them lower quotas.

Many firms preempt such lobbying by implementing a formal process through which individuals provide input into quotas. For example, some use a bottom-up process in which salespeople and/or managers provide estimates of sales potential at the territory or customer level, which are then aggregated at higher levels. In fact, a combined top-down/bottom-up approach is commonly discussed by practitioners and incentive design specialists as a best practice (e.g., Eddleman 2012; SalesGlobe n.d.) and has been built into popular enterprise software, such as Oracle Sales Planning Cloud (Oracle 2018). Top-down and bottom-up approaches used alone have critical flaws, as summarized by Capon and Go (2017): “Bottom-up forecasts embrace the granularity (and reality) of sales by customer, absent from top-down forecasts…[but] a serious downside of bottom-up forecasting is salespeople lowballing estimates when forecasts drive sales quotas.” However, reconciling top-down and bottom-up systems typically requires time-consuming (and often tense) negotiations and does not necessarily ensure accurate or fair results.

The first objective of this article is to propose an approach that builds and improves on common practices to achieve the benefits of bottom-up incentive design (incorporating local-level information) while avoiding the primary downside (“lowballing” by salespeople and sales managers). The proposed approach is simpler than top-down/bottom-up negotiations and much more objective and reliable than informal processes such as behind-the-scenes lobbying and ad hoc adjustments. The second objective is to show how the proposed approach can be optimally implemented and to identify when this optimal implementation outperforms the standard theoretical solution of a menu of contracts presented by the firm to its salespeople. Thus, this article shows how to efficiently involve a sales manager in sales force compensation design and when it is optimal to do so.

These objectives are achieved by extending the principal–agent model to include a sales manager. The resulting model captures two common elements of sales force compensation design reflected in the previous examples that are not captured by standard models. The first is information asymmetry between the firm, sales managers, and salespeople. One naturally expects salespeople to be well-informed about themselves and their territories. However, first-line sales managers also spend an average of 48% of their time “in the trenches” with customers and/or salespeople (Fritz 2008). Thus, though the manager in this model is not quite as well-informed as the salesperson, she has better local information than others in the firm who are further removed from territory-level dynamics. In the model, the local information relates to the difficulty of a salesperson’s territory, but the results apply to any case in which the manager has better information than the firm about something relevant to a salesperson’s compensation plan (e.g., his selling ability).

This information gap between firm and manager is irrelevant if the manager always shares her local information openly with the firm. However, she may choose not to do so if her interests and the firm’s interests are not aligned, which is the second new element reflected in my model. Though the firm is primarily interested in profits, a recent survey found that 89.5% of companies base their incentives for first-line sales managers on revenue (Alexander Group 2018). As in the previous examples, this can result in managers misrepresenting their information (e.g., arguing for unnecessarily high commissions or low quotas) to maximize their own expected payouts. In line with the data, the manager in my model has a revenue-based incentive plan.^2^ However, this issue arises (and the model applies) under any type of managerial compensation plan, including non-revenue-based plans, as long as the manager’s expected payout increases with the salesperson’s selling effort. Furthermore, the model is not limited to a particular plan type (e.g., commission-based, quota-based) and instead captures the general case in which a sales manager has an opportunity to provide input into the incentive plan of a salesperson she manages.^3^ The manager in the model most closely represents a first-line sales manager in most firms but can be any individual who possesses local information and earns incentives that increase with salesperson effort.

Under my proposed mechanism, the firm delegates the salesperson’s incentive parameters to the manager subject to tight constraints, such as limits on the salesperson’s salary or incentive pay. The manager can submit a request to relax those constraints by meeting requirements imposed by the firm. Relaxed constraints always benefit the manager because they allow her to incentivize the salesperson to work harder, thereby increasing her own expected payout. However, the firm can always set the requirements for such requests such that the manager will make a request only when doing so is best for the firm given her information. Thus, the firm can replace internal negotiations and lobbying with an efficient, reliable process that reveals the manager’s true information.

To illustrate what this might look like in practice, consider the example of Firm A discussed earlier. To assign commission rates to salespeople, the firm asked its sales managers to identify the degree of difficulty of each territory but found it difficult to distinguish unbiased input from misrepresentations. Under my proposed mechanism, Firm A would have begun by assigning all territories the lowest commission rate and establishing clear requirements that sales managers had to fulfill to request the medium rate and the high rate. A request for the medium rate could require a manager to complete onerous paperwork, attend a series of meetings with an immediate supervisor, and/or submit a business case outlining the factors that make the territory difficult. A subsequent request for the high rate might require her to submit a detailed territory analysis, provide customer-level projections, and give a presentation to senior management. When the firm’s requirements are set optimally, managers will prefer to submit requests only when the resulting changes in the commission rate benefit the firm, so every completed request should be granted.

The model gives direction regarding the amount of effort each request should require but is indifferent to the nature of the requirements, which can vary widely depending on context. Notably, the model does not require the cost of the manager’s effort to depend on her actual information (i.e., it need not be more costly for the manager to make a false claim than a truthful one). This allows the firm to introduce *any* requirements that impose a cost on the manager, not just ones related to her information (such as a territory analysis or business case). Indeed, even common practices such as top-down/bottom-up negotiations can be viewed as a way to impose a cost on sales managers seeking relaxed incentive constraints. In that sense, firms can use the proposed mechanism to increase the reliability and transparency of such practices.

When a firm is not fully informed about a salesperson or his territory, the well-accepted theoretical solution has been to allow the salesperson to choose from a menu of contracts (e.g., Lal and Staelin 1986; Rao 1990). Suppose, for example, that territories are either “easy” or “hard,” but the firm does not know which are which, as in the case of Firm A. Existing theory tells us that the firm can design a menu such that a salesperson will choose the contract intended for his territory type, effectively revealing his private information. Critically, however, optimizing this menu requires the firm to know the likelihood that a given territory is easy or hard. Furthermore, determining that likelihood depends on precisely the kind of local information that the firm lacks relative to sales managers and salespeople. Consequently, even the standard solution requires information that the firm does not necessarily have.

The proposed request mechanism adds value by revealing the sales manager’s superior local information, and it outperforms a menu of contracts under broad conditions. Specifically, I show that involving sales managers in designing incentives for their salespeople is optimal when the managers’ sales incentives are not too large or when the optimal menu of contracts results in a salesperson exiting from some types of territories.

This research offers managerial insights for firms that wish to leverage sales managers’ information to design sales force incentives. The proposed mechanism mitigates a manager’s preference for inflating her salespeople’s incentives, allowing the firm to reliably obtain her information using a transparent and efficient process.

In the following sections, I summarize this article’s contributions to the literature, introduce the main model, and discuss the analysis and results. I then present two extensions of the model that demonstrate its robustness and provide additional insights. Finally, I review the key findings and discuss related research opportunities.

## Literature Review

This research is most closely related to the literature on sales force incentive design (e.g., Basu et al. 1985; Hauser, Simester, and Wernerfelt 1994; Joseph and Thevaranjan 1998), joining studies that explore the design of incentives for a salesperson whose type is unobserved by his firm (e.g., Chen, Xu, and Liu 2013; Gonik 1978; Lal and Staelin 1986; Mantrala and Raman 1990; Rao 1990). A core element of these studies is the firm offering a menu of contracts, the optimal design of which requires full information about the probability distribution of the agent’s type. To my knowledge, this article offers the first analysis of a model in which an uninformed firm can delegate part of the incentive design process to an intermediary (a sales manager) who has useful information but also has divergent interests. I propose a mechanism that can be used as an alternative or a complement to the menu of contracts. Furthermore, Krafft et al. (2012, p.107) mention the need for greater attention to “the interplay between superiors and subordinates across sales force management layers in the context of compensation/control.” My article makes a meaningful contribution to the literature on sales management by exploring a game-theoretic model of this interplay.

This study relates to two areas of research in economics: “influence activities” and delegation in organizations. In the proposed mechanism, the firm requires the manager to engage in influence activities, defined as effort exerted by an individual to affect an organization’s decisions, to relax constraints on a salesperson’s compensation design. Most of the literature in this area (e.g., Milgrom 1988; Wulf 2009) focuses on limiting influence activities because they are viewed as wasteful and inefficient. This article joins a small subset showing that such activities can benefit a firm by revealing private information held by the influencing individual. My three-player model (firm–manager–salesperson) is a significant extension of the two-player models in Laux (2008) (firm–manager) and Simester and Zhang (2014) (firm–salesperson). Unlike Laux (2008), I assume that the manager acquires information without cost (with an extension considering costly supplementation), and I focus on truthful revelation. Furthermore, the manager’s tendency to overinvest arises endogenously from incentives in my model, whereas Laux (2008) assumes a taste for “empire building.”

In Simester and Zhang (2014), the salesperson is perfectly informed about a customer’s demand and can lobby for permission to charge a lower price. The findings rely on an assumption that lobbying is more costly to the salesperson when it is not supported by his information. I show that a similar mechanism is feasible in a three-player incentive design model even when the lobbying individual (the manager) is only partially informed. Furthermore, my use of influence activities as a separating mechanism does not rely on an assumed difference in the cost of lobbying; rather, it is based on a derived difference in the benefit to the manager of exerting influence “truthfully” versus “untruthfully.” This significantly expands both the applicability of the mechanism and the range of lobbying requirements the firm can impose. In fact, the firm can impose virtually any requirement as long as it is costly to the manager.

Lastly, my model fits within the “general formulation of the decentralization problem” (Holmström 1984), focusing on delegation within a three-level hierarchy. The economic literature on hierarchies focuses on the threat of collusion (e.g., Imbeau 2007; Laffont 1990; Tirole 1986) and on inefficiencies introduced by delegation (e.g., Faure-Grimaud and Martimort 2001; McAfee and McMillan 1995). One exception is Mookherjee and Reichelstein (1997), which shows that delegation can be as efficient as centralization when production is deterministic and managers have no private knowledge about their subordinates. This is achieved by allowing each individual to offer a menu of incentive schemes to their direct subordinates. In contrast, I study a non-menu-based mechanism in an environment with stochastic production (sales) and information asymmetry between the layers of the hierarchy. The marketing literature also contains studies of incentive design in settings with more than two players (principal and agent) but these typically focus on collusion in nonhierarchical structures (e.g., Hauser, Simester, and Wernerfelt 1996, 1997). In the context of sales management, analytical research on delegation has focused on the conditions under which delegation of pricing to the sales force is optimal (e.g., Bhardwaj 2001; Lal 1986; Mishra and Prasad 2004, 2005).

## Model

I analyze a three-player model of a firm, a sales manager, and a salesperson. The model is defined as follows.

### Salesperson

The firm employs a salesperson to sell a product. For simplicity, product sales take one of two values: $x\in \{x_{L},x_{H}\}$ with $\text{Δ}x\equiv x_{H}-x_{L}>0$ .

The salesperson exerts effort to sell the product, but his effort is costly and is not observed by the firm. Again, for simplicity, effort takes one of two values: $e\in \{e_{L},e_{H}\}$ with $\text{Δ}e\equiv e_{H}-e_{L}>0$ and $e_{L}>0$ . If the salesperson exits the firm, e=0 but sales of $x_{L}$ or $x_{H}$ still occur.^4^

The salesperson’s effort drives the firm’s expected sales by increasing the probability of the high sales outcome, p(e) . However, there is always a degree of uncertainty, so the firm cannot infer his effort from the observed outcome. Therefore, p(e) takes the value $p_{0}\equiv \text{Pr}(x=x_{H}|e=0)$ , $p_{L}\equiv \text{Pr}(x=x_{H}|e=e_{L})$ , or $p_{H}\equiv \text{Pr}(x=x_{H}|e=e_{H})$ with $0\leq p_{0}<p_{L}<p_{H}<1$ .

Because the firm does not observe the salesperson’s effort, it offers him incentives based on sales. Sales outcomes are binary, so the salesperson’s compensation plan is represented by a pair of payout values. He receives $s(x)=\left\{\begin{array}{c} \begin{array}{ll} s_{L} & \text{if }x=x_{L}\\ s_{H} & \text{if }x=x_{H} \end{array} \end{array}\right.$ , where $s_{H}\geq s_{L}\geq 0$ . This can be thought of as a fixed salary $s_{L}$ plus an outcome-dependent bonus $\text{Δ}s=s_{H}-s_{L}$ .

The salesperson receives increasing positive utility u(s)=s from income. This linear function implies that the salesperson is risk neutral but his contract includes a limited liability condition. As discussed in Dai and Jerath (2013), limited liability ensures that compensation is never negative, as is common in sales force contracts and in the literature.

The salesperson incurs increasing positive disutility v(e) from exerting effort. Given the binary levels of effort, a simple linear cost function is sufficient: v(e)=βe .

The salesperson’s territory takes one of two types: low and high difficulty (or “easy” and “hard”), represented by $\beta \in \{\beta_{L},\beta_{H}\}$ with $0<\beta_{L}<\beta_{H}$ . In other words, territory type is reflected in the salesperson’s cost of effort, with an easy territory imposing less cost than a hard one for the same effect on sales outcomes.^5^ The salesperson’s utility from income and disutility from effort are assumed to be additively separable, so his total utility is $U_{s}=u(s(x))-v(e)=s(x)-\beta e$ .

If the salesperson exits the firm, his best alternative option provides utility $\bar{U}\geq 0$ . If his expected utility from a contract is less than $\bar{U}$ , he rejects it, exits, and receives no compensation. I assume that the firm is unable to hire and train a replacement for him within the time frame of the model. For simplicity, I also assume that $\bar{U}\geq \beta_{H}(\frac{p_{L}e_{H}-p_{H}e_{L}}{p_{H}-p_{L}})\geq 0$ , which ensures that the limited liability constraint never prevents the firm from achieving its best possible result. This can be thought of as restricting attention to cases in which the salesperson receives a nonzero salary.

### Sales Manager

The sales manager in the model can represent a manager at any level but ideally one with the best information about the salesperson’s territory aside from the salesperson. The manager’s compensation has the same structure as the salesperson’s: $m(x)=\left\{\begin{array}{cc} m_{L} & \text{if }x=x_{L}\\ m_{H} & \text{if }x=x_{H} \end{array}\right.$ , with $\text{Δ}m\equiv m_{H}-m_{L}>0$ . Once again, this can be thought of as a fixed salary $m_{L}$ plus an outcome-dependent bonus $\text{Δ}m=m_{H}-m_{L}$ . Given the binary sales outcomes in the model, the salesperson’s and manager’s plans can be nearly any common type of sales incentive (quota bonus, commission, etc.). The manager’s incentive pay can also be a scaling of the salesperson’s incentive pay (common in practice). Side payments between the manager and salesperson are assumed to be prevented by ethical considerations and/or firm regulations (enforced without cost).

In practice, sales managers have a broad range of responsibilities, many of which can affect sales directly or indirectly. Consequently, most managers have sales-based incentives, reflected by Δm>0 in my model. To focus on the manager’s role in designing salesperson compensation, I omit her effort on other tasks from the main analysis.^6^ Instead, I assume that Δm is set exogenously by the firm and is sufficient to ensure that the manager exerts the desired effort on those tasks. However, I include a model extension in which the manager’s compensation (including Δm ) is fully optimized. Details of that model are provided in the Model Extensions section.

Like the salesperson, the manager is risk neutral and obtains utility from income u(m)=m but her contract includes a limited liability condition ( $m_{L}\geq 0$ ). The manager can exert effort $e_{m}^{r}\geq 0$ to request relaxed constraints on the salesperson’s compensation plan (further discussed subsequently). Her disutility from that effort is given by $v(e_{m}^{r})=e_{m}^{r}$ .

The components of her utility are assumed to be additively separable. Therefore, the manager’s utility is $U_{m}=u(m(x))-v(e_{m}^{r})=m(x)-e_{m}^{r}$ , where $e_{m}^{r}=0$ when the manager does not make a request. The manager’s objective is to maximize her expected utility. When she is indifferent between actions, I assume that she chooses the one that is best for the firm.

The manager’s best alternative option provides utility ${\bar{U}}_{m}>0$ . If her expected utility from a contract is less than ${\bar{U}}_{m}$ , she rejects it and exits the firm. In the main model, I assume that this is never optimal for the firm, even if the salesperson exits. This assumption is relaxed in Model Extension 1, in which the manager’s other responsibilities are captured more explicitly. I also assume that ${\bar{U}}_{m}\geq p_{H}\text{Δ}m$ , so the limited liability constraint never prevents the firm from achieving its best possible result.

### Firm

The firm maximizes expected profit with its marginal production cost normalized to 0.

$$E[\pi ]=\left\{\begin{array}{l} p_{i}(x_{H}-m_{H}-s_{H})+(1-p_{i})(x_{L}-m_{L}-s_{L})\\ \text{ if salesperson exerts effort }e_{i}\\ p_{0}(x_{H}-m_{H})+(1-p_{0})(x_{L}-m_{L})\\ \text{ if salesperson exits} \end{array}\right.$$

When the firm delegates the salesperson’s compensation design to the manager, it imposes constraints on her choices. Those constraints can take the form of hard caps on any subset of the salesperson’s low payout (i.e., a salary cap $\hat{s_{L}}$ ), high payout (i.e., a total payout cap $\hat{s_{H}}$ ), and/or the difference between the two (i.e., a bonus cap $\hat{\text{Δ}s}$ ).

### Information

As previously noted, the salesperson’s territory can be easy or hard, denoted by $\beta \in \{\beta_{L},\beta_{H}\}$ . Despite firm efforts to design balanced territories, selling difficulty can vary for many reasons, including geographic constraints and differences in customer composition. Territory boundaries and selling conditions can also change frequently (Zoltners and Sinha 1983), making it difficult for firms to maintain up-to-date information or to use a territory’s historical performance to infer its current degree of difficulty. Only the salesperson is assumed to know the true value of β with certainty. However, a sales manager acquires information about the territory in the course of her regular duties, providing her with greater information than the firm at no cost. Therefore, the salesperson, manager, and firm have different degrees of information about a territory’s type.

In the model, the firm assigns a probability of 50% to each territory type, which is represented by $\gamma_{L}^{F}=Pr(\beta =\beta_{L})=.5$ . The firm correctly believes that half of all territories are hard but has no indication about an individual territory’s type. Thus, this can be thought of as an accurate but uninformed prior.

The manager begins with the same uninformed prior but receives a signal, $\beta_{L}$ or $\beta_{H}$ , with signal quality Q∼U(.5,1) . In other words, the signal matches the true territory type with probability Q . The range of Q indicates that the signal contains some information but is imperfect. Both the signal and its quality are known only to the manager (though its distribution is common knowledge) so she not only acquires information about the territory but is uniquely positioned to assess the quality of that information. I denote the manager’s postsignal belief about the territory type as $\gamma_{L}=Pr(\beta =\beta_{L})$ and restrict attention to cases in which the firm prefers to employ the salesperson (rather than letting him exit) even when the territory is known to be hard. In Model Extension 2, the manager can incur an additional cost to learn the territory type with certainty (i.e., to receive a new signal of quality Q=100% ).

### Sequence of Events

0. Salesperson learns territory type ( $\beta_{L}$ or $\beta_{H}$ ), manager receives signal, firm determines manager’s incentive pay (Δm) .
1. Firm announces manager’s contract $\left(m_{L},m_{H}\right)$ .
2. Manager decides whether to accept the contract.
3. Firm announces baseline constraints on salesperson’s compensation $\left(\hat{s_{L}},\hat{s_{H}},\hat{\text{Δ}s}\right)$ , requirements for manager to relax those constraints $\left(e_{m}^{r}\right)$ , and resulting relaxed constraints (sL^ ',sH^',Δs^') . (This can include a second set of requirements and relaxed constraints in some cases.)
4. Manager chooses request effort $\left(e_{m}^{r}\right)$ , firm sets final constraints accordingly.
5. Manager announces salesperson’s contract $\left(s_{L},s_{H}\right)$ .
6. Salesperson decides whether to accept contract and, if so, chooses effort level.
7. Sales are realized and all players receive payouts.

The events in Step 0 are exogenous to the model. Steps 2 and 3 can be reversed with no effect on the results.^7^ Accepted contracts are assumed to be binding—the key findings do not apply if the firm can renege on the manager’s contract after observing her choices in subsequent steps. If the manager plays a role in designing the salesperson’s territory, that process is assumed to be completed before the model sequence begins (i.e., the salesperson’s territory must be finalized before his compensation terms are set).

The analysis includes a comparison of the main model to a menu of contracts model in which Steps 3 and 4 are omitted, the firm announces the menu of contracts in Step 5, and the salesperson chooses from that menu (or declines all contracts) in Step 6. The notation introduced here and throughout the article is summarized in Table 1.

**Table 1.** Summary of Notation.

**Notation**	**Definition**
$x_{L}$ , $x_{H}$	Possible sales outcomes
Δx	Difference between possible sales outcomes $\left(x_{H}-x_{L}\right)$
$e_{L}$ , $e_{H}$	Possible effort levels exerted by salesperson
Δe	Difference between salesperson’s possible effort levels $\left(e_{H}-e_{L}\right)$
$e_{m}^{r}$	Request effort exerted by manager
p(e) , $p_{0}$ , $p_{L}$ , $p_{H}$	Probability of high sales outcome under efforts e , 0, $e_{L}$ , $e_{H}$ , respectively
$s_{L}$ , $s_{H}$	Salesperson’s compensation payouts corresponding to $x_{L}$ , $x_{H}$ , respectively
Δs	Difference between salesperson’s compensation payouts $\left(s_{H}-s_{L}\right)$
$\hat{s_{L}}$ , $\hat{s_{H}}$ , $\hat{\text{Δ}s}$	Constraints (caps) imposed by firm on $s_{L}$ , $s_{H}$ , Δs , respectively
$m_{L}$ , $m_{H}$	Manager’s compensation payouts corresponding to $x_{L}$ , $x_{H}$ , respectively
Δm	Difference between manager’s compensation payouts $\left(m_{H}-m_{L}\right)$
$U_{s}\;(U_{m})$	Salesperson’s (manager’s) total utility
$\bar{U}\;({\bar{U}}_{m})$	Salesperson’s (manager’s) utility from outside option
u(⋅)	Salesperson’s and manager’s utility from income
v(⋅)	Salesperson’s and manager’s disutility from effort (i.e., effort cost)
$\beta \in \{\beta_{L},\beta_{H}\}$	Difficulty of selling in salesperson’s territory (i.e., territory type)
Q	Quality of manager’s signal about territory type
$\gamma_{L}$	Manager’s belief about probability that territory is easy (i.e., $Pr(\beta =\beta_{L})$ )
$f(\gamma_{L})$	Ex ante probability density function of manager’s belief
$\gamma_{L}^{F}$	Firm’s belief about probability that territory is easy
$e^{\beta_{L}},e^{\beta_{H}}$	Salesperson’s optimal effort if $\beta =\beta_{L}$ , $\beta_{H}$ , respectively
E[π]	Firm’s expected profit
$e_{m}^{s}\in \{0,e_{m}^{s*}\}$	Sales support effort exerted by manager (in Extension 1)
$p_{m}(e_{m}^{s})$	Increase in probability of high sales due to manager’s support effort (in Extension 1)
C	Manager’s cost of additional information (in Extension 2)

## Analysis and Results

The model is solved using backward induction.

### Salesperson’s Contract Acceptance and Effort Choice (Step 6)

Given a contract $\left(s_{L},s_{H}\right)$ , the salesperson either exits or chooses effort $e_{i}$ , i∈{L,H} , to satisfy:

$$\begin{array}{l} s_{L}+p(e_{i})\text{Δ}s-\beta e_{i}\geq \bar{U}\;({\text{IR}}_{i})\\ s_{L}+p(e_{i})\text{Δ}s-\beta e_{i}\geq s_{L}+p(e_{j})\text{Δ}s-\beta e_{j}\;\forall j\in \{L,H\}\;({\text{IC}}_{i}). \end{array}$$

The individual rationality condition $\left({\text{IR}}_{i}\right)$ ensures that the salesperson’s expected utility does not fall below his outside option utility $\bar{U}$ . If $\left({\text{IR}}_{i}\right)$ is not satisfied for either i , he exits the firm. Otherwise, he accepts the contract and chooses the effort that satisfies the incentive compatibility condition $\left({\text{IC}}_{i}\right)$ , which ensures that effort $e_{i}$ provides the greatest possible expected utility. This can be restated as follows. The salesperson prefers $\left\{\begin{array}{cc} e_{L} & \text{if }\beta \text{Δ}e\geq (p_{H}-p_{L})\text{Δ}s\\ e_{H} & \text{if }\beta \text{Δ}e\leq (p_{H}-p_{L})\text{Δ}s \end{array}\right.$ .

Intuitively, this indicates that the salesperson will increase his effort when the corresponding increase in expected income outweighs the cost of the incremental effort. As β increases, this is less likely to be true and $\left({\text{IR}}_{i}\right)$ is less likely to hold for either i . Thus, for a given contract, the salesperson’s effort (weakly) decreases with his territory’s difficulty.

Throughout the remainder of this article, I use the following terminology and notation. A contract induces the effort combination $\left(e^{\beta_{L}},e^{\beta_{H}}\right)$ if the salesperson’s optimal response is to exert $e^{\beta_{L}}$ when the territory is easy and $e^{\beta_{H}}$ when it is hard, with $e^{\beta_{i}}=0$ if he exits.

### Manager’s Design of Salesperson’s Contract (Step 5)

Having received a signal about the territory’s difficulty and observed the signal quality, the manager uses Bayesian updating to generate the following belief about the salesperson’s territory type:

$$\gamma_{L}\equiv P(\beta =\beta_{L})=\left\{\begin{array}{cc} Q & \text{if signal}=\beta_{L}\\ 1-Q & \text{if signal}=\beta_{H} \end{array}\right.$$

Subject to the constraints imposed by the firm, the manager chooses the salesperson’s compensation plan $\left(s_{L},s_{H}\right)$ to maximize her expected utility: $E[U_{m}]=m_{L}+\left[\gamma_{L}p(e^{\beta_{L}})+(1-\gamma_{L})p(e^{\beta_{H}})\right]\text{Δ}m-e_{m}^{r}$ . At this point, the manager’s effort $e_{m}^{r}$ is a sunk cost, so this objective function simplifies to

1 $$\underset{\left(s_{L},s_{H}\right)}{\text{max}}\gamma_{L}p(e^{\beta_{L}})+(1-\gamma_{L})p(e^{\beta_{H}})$$

In other words, the manager maximizes her expected utility by maximizing the likelihood of high sales. That likelihood depends on the salesperson’s effort which, in turn, depends on his territory type. Thus, in assessing a contract for the salesperson, the manager considers her belief about the territory $\left(\gamma_{L}\right)$ and the level of effort that the contract will induce in each territory type $\left(e^{\beta_{L}},e^{\beta_{H}}\right)$ .

**Lemma 1:** In the absence of constraints, there exists a contract that induces any combination of effort levels $\left(e^{\beta_{L}},e^{\beta_{H}}\right)$ with $e^{\beta_{L}}\geq e^{\beta_{H}}$ .

The manager is indifferent between contracts that induce a given combination of effort levels $\left(e^{\beta_{L}},e^{\beta_{H}}\right)$ , so Lemma 1 (proof in Web Appendix A) implies that designing the salesperson’s contract can be thought of as simply choosing an effort combination from among the combinations that her constraints allow. From Equation 1, the manager always prefers the highest effort in each territory type, so most of her preferences among these options are clear:


$$(0,0)≺(e_{L},0)≺\begin{array}{l} \left(e_{L},e_{L}\right)\\ \;(e_{H},0) \end{array}≺(e_{H},e_{L})≺(e_{H},e_{H}),$$

where a≺b denotes a strict preference for b over a .

Her preference between the two options in the middle, $\left(e_{L},e_{L}\right)$ and $\left(e_{H},0\right)$ , depends on her belief, $\gamma_{L}$ . If the territory is likely to be easy, she prefers $\left(e^{\beta_{L}},e^{\beta_{H}})=(e_{H},0\right)$ to ensure high effort in the most likely case. If the territory is likely to be hard, she prefers $\left(e_{L},e_{L}\right)$ , sacrificing some effort should the territory turn out to be easy in order to ensure that the salesperson does not exit if it is hard.

Left unconstrained, the manager always prefers $\left(e_{H},e_{H}\right)$ , which maximizes expected sales with high effort regardless of territory type. However, that is not always optimal for the firm,^8^ so the firm imposes constraints on the salesperson’s contract when delegating its design to the manager.

### Manager’s Choice of Request Effort (Step 4)

Given a set of constraints $\left(\hat{s_{L}},\hat{s_{H}},\hat{\text{Δ}s}\right)$ , the manager will offer the salesperson a contract that results in her most preferred effort combination based on her preferences in Step 5. Suppose the firm imposes baseline constraints that allow her to induce $\left(e^{\beta_{L}},e^{\beta_{H}}\right)$ and offers her the opportunity to request relaxed constraints that allow her to induce (eβL',eβH') . If making that request requires the manager to exert effort $e_{m}^{r*}$ then she prefers to make it if and only if she prefers (eβL',eβH') over $\left(e^{\beta_{L}},e^{\beta_{H}}\right)$ and


mL+γLp(eβL')+(1−γL)p(eβH')Δm−emr*>mL  +γLp(eβL)+(1−γL)p(eβH)Δm

⇔γLp(eβH')−p(eβH)−p(eβL')−p(eβL)Δm  <p(eβH')−p(eβH)Δm−emr*.

As shown in the next step, the case in which eβL=eβL' is of particular interest. In that case, the manager requests relaxed constraints i


2 γL<p(eβH')−p(eβH)Δm−emr*p(eβH')−p(eβH)Δm.

Thus, the manager requests relaxed constraints when $\gamma_{L}$ is sufficiently low. This is intuitive because eβL=eβL' implies that the manager benefits from a request only when the territory is hard. Therefore, she is willing to exert effort only if a hard territory is sufficiently likely. As expected, the manager is more likely to make a request (because the threshold in Equation 2 is higher) when the required effort to make it $\left(e_{m}^{r*}\right)$ is lower.

### Firm’s Choice of Constraints and Request Requirements (Step 3)

Like the manager, the firm can anticipate the salesperson’s level of effort for each territory type under a given contract. It seeks an efficient, implementable contract to induce a given effort combination $\left(e^{\beta_{L}},e^{\beta_{H}}\right)$ . A contract is “efficient” if it minimizes the firm’s expected incentive cost given the desired effort combination and is “implementable” if the firm can set constraints such that the manager will choose to offer it.

**Lemma 2:** A hard cap on the salesperson’s total payout ( $\hat{s_{H}}$ ) along with a hard cap on either his salary ( $\hat{s_{L}}$ ) or his bonus ( $\hat{\text{Δ}s}$ ) is sufficient to implement an efficient contract for any effort combination $\left(e^{\beta_{L}},e^{\beta_{H}}\right)$ with $e^{\beta_{L}}\geq e^{\beta_{H}}$ .

See Appendix A for sufficient constraints to implement each effort combination and Web Appendix B for a complete derivation. Lemma 2 implies that, in this stylized model, hard caps are sufficient for the firm to determine the salesperson’s effort in each territory type. In practice, sales outcomes and compensation plans are complex and may require more complex constraints. For example, a firm might choose a quota-based plan and set the payout for each level of quota attainment, then allow the manager to set the salesperson’s quota, perhaps within certain bounds.

Under incomplete information, the efficiency of a contract can be characterized by the expected surplus it provides the salesperson in each territory type relative to his outside option.

**Lemma 3:** An efficient contract to induce efforts $\left(e^{\beta_{L}},e^{\beta_{H}}\right)$ has the following properties:
(a) When the territory is hard, the salesperson receives no surplus.
(b) When the territory is easy, the salesperson receives an expected utility surplus of $\text{Δ}\beta e^{\beta_{H}}$ .

Lemma 3 (proof in Web Appendix C) indicates that the firm must pay the salesperson “information rent” when the territory is easy. Furthermore, that rent increases with the salesperson’s effort when the territory is hard $\left(e^{\beta_{H}}\right)$ . Thus, the firm faces a trade-off between the effort it desires in a hard territory and the rent it pays in an easy one. This trade-off is weighted by the probability of each territory type. For example, suppose a fully informed firm would choose to induce high effort in all territories. If a firm with imperfect information believes that the territory is likely to be easy, it might prefer to offer a contract that induces less effort (or even exit) when the territory is hard to reduce the rent it must pay to the salesperson in the (more likely) event that it is easy.

Lemma 3 also indicates that the salesperson’s contract is always efficient when the territory is hard. Thus, the effort expected in an easy territory has no effect on the firm’s profitability if the territory is hard. In other words, the trade-off only works in one direction, so the firm’s preferred effort in an easy territory is independent of its belief about the territory type.

**Proposition 1:** The firm’s best possible contract has the following properties:

 (a) The effort it induces in a hard territory (weakly) decreases with $\gamma_{L}$ .
 (b) The effort it induces in an easy territory is independent of $\gamma_{L}$ . In this setting, that effort is always $e_{H}$ .

See Appendix B for a detailed proof of Proposition 1. Part (b) implies that the firm can narrow its consideration to constraints that result in the “correct” effort $\left(e_{H}\right)$ when the territory is easy. Thus, the firm’s constraints must result in an efficient contract for $\left(e^{\beta_{L}},e^{\beta_{H}})=(e_{H},0\right)$ , $\left(e_{H},e_{L}\right)$ , or $\left(e_{H},e_{H}\right)$ . That $e^{\beta_{L}}$ is always $e_{H}$ is due to the simplifying assumptions ensuring that the limited liability condition does not prevent the best possible solution and that the firm prefers to employ the salesperson even when his territory is known to be hard. That result simplifies the remaining analysis but is not necessary to obtain the main results.

Part (a) reflects the trade-off mentioned previously, with the firm preferring to induce less effort in a hard territory when that type is less likely. This implies that the firm’s ability to choose the best contract depends on the accuracy of its belief about the territory type. Thus, the firm would prefer to rely on the manager’s belief, rather than its own less-informed belief, to determine $e^{\beta_{H}}$ . However, the firm also knows that the manager wants to induce the greatest possible effort. Thus, the manager has an incentive to report that the territory is likely to be hard regardless of her true belief. This is consistent with the introductory examples in which sales managers negotiated for higher commissions or lower quotas by claiming that their salespeople’s territories were particularly difficult. The request mechanism proposed in this model is intended to reduce managers’ tendency to overstate territory difficulty.

**Proposition 2:** The firm can always define baseline and relaxed constraints and corresponding request requirements such that the manager will choose to request relaxation of the constraints only when doing so is best for the firm, given her belief about the territory type. Therefore, the request mechanism is always feasible.

The constraints and request requirements chosen by the firm are summarized in Table 2. See Appendix C for derivation of these results and the proof of Proposition 2.

**Table 2.** Summary of Constraints and Request Requirements.

**Case^a^**	**Pre-request** $(e^{\beta_{L}},e^{\beta_{H}})$	**Post-request** (eβL',eβH')	**Request Effort** $(e_{m}^{r})$ **^a^**
$(p_{L}-p_{0})(\text{Δ}x-\text{Δ}m)<\bar{U}+\beta_{H}e_{L}$ OR $\gamma_{L}^{0}<\gamma_{L}^{2}$	$\left(e_{H},0\right)$	$\left(e_{H},e_{H}\right)$	$(1-\gamma_{L}^{1})(p_{H}-p_{0})\text{Δ}m$
Otherwise	$\left(e_{H},0\right)$	$\left(e_{H},e_{L}\right)$	$(1-\gamma_{L}^{0})(p_{L}-p_{0})\text{Δ}m$
	$\left(e_{H},e_{L}\right)$	$\left(e_{H},e_{H}\right)$	$(1-\gamma_{L}^{2})(p_{H}-p_{L})\text{Δ}m$

^a^From the proof of Proposition 2 in Web Appendix C:

$\gamma_{L}^{0}=\frac{\left(p_{L}-p_{0})(\text{Δ}x-\text{Δ}m)-(\bar{U}+\beta_{H}e_{L}\right)}{\left(p_{L}-p_{0})(\text{Δ}x-\text{Δ}m)-(\bar{U}+\beta_{L}e_{L}\right)}$

$\gamma_{L}^{1}=\frac{\left(p_{H}-p_{0})(\text{Δ}x-\text{Δ}m)-(\bar{U}+\beta_{H}e_{H}\right)}{\left(p_{H}-p_{0})(\text{Δ}x-\text{Δ}m)-(\bar{U}+\beta_{L}e_{H}\right)}$

$\gamma_{L}^{2}=\frac{(p_{H}-p_{L})(\text{Δ}x-\text{Δ}m)-\beta_{H}\text{Δ}e}{(p_{H}-p_{L})(\text{Δ}x-\text{Δ}m)-\beta_{L}\text{Δ}e}$

As shown in Table 2, the firm’s choices are divided into two distinct cases. The first occurs when the firm does not prefer $\left(e^{\beta_{L}},e^{\beta_{H}})=(e_{H},e_{L}\right)$ under any belief, narrowing its options to $\left(e_{H},0\right)$ or $\left(e_{H},e_{H}\right)$ . By Lemma 2, then, the firm can set baseline constraints such that the manager chooses an efficient contract to induce $\left(e_{H},0\right)$ , relaxed constraints that allow her to efficiently induce $\left(e_{H},e_{H}\right)$ , and required effort $e_{m}^{r}=e_{m}^{r*}$ to request relaxation of the constraints.

By Proposition 1, the firm prefers $\left(e_{H},e_{H}\right)$ only when the manager’s belief, $\gamma_{L}$ , is below some decision threshold $\gamma_{L}^{1}$ (i.e., when the likelihood of the territory being hard is sufficiently high). (From the proof in Appendix B, $\gamma_{L}^{1}=\frac{\left(p_{H}-p_{0})(\text{Δ}x-\text{Δ}m)-(\bar{U}+\beta_{H}e_{H}\right)}{\left(p_{H}-p_{0})(\text{Δ}x-\text{Δ}m)-(\bar{U}+\beta_{L}e_{H}\right)}$ .) Thus, Proposition 2 implies that $e_{m}^{r*}$ can be chosen such that the manager only makes a request when $\gamma_{L}<\gamma_{L}^{1}$ . Indeed, Equation 2 indicates that the manager makes a request if and only if $\gamma_{L}<\frac{\left(p_{H}-p_{0}\right)\text{Δ}m-e_{m}^{r*}}{\left(p_{H}-p_{0}\right)\text{Δ}m}$ . Therefore, if the firm sets $e_{m}^{r*}=(1-\gamma_{L}^{1})(p_{H}-p_{0})\text{Δ}m$ , then $\frac{(p_{H}-p_{0})\text{Δ}m-e_{m}^{r*}}{(p_{H}-p_{0})\text{Δ}m}=\gamma_{L}^{1}$ and the manager makes the request if and only if $\gamma_{L}<\gamma_{L}^{1}$ , as desired.

In the second case, $\left(e_{H},0),\;(e_{H},e_{L}\right)$ , or $\left(e_{H},e_{H}\right)$ can be best for the firm depending on $\gamma_{L}$ . Thus, the firm allows multiple requests resulting in different levels of constraints. The baseline constraints allow the manager to induce $\left(e_{H},0\right)$ . If she makes the first request, the resulting relaxed constraints allow $\left(e_{H},e_{L}\right)$ . She can then make a second request, leading to constraints that allow $\left(e_{H},e_{H}\right)$ . Here, Proposition 2 indicates that the firm can set $e_{m}^{rL}$ and $e_{m}^{rH}$ for the first and second requests, respectively, such that the manager will make each request only when it is best for the firm.

### Manager’s Contract Acceptance (Step 2)

When deciding whether to accept the firm’s contract offer, the manager can anticipate how the firm will implement the request mechanism. She can also anticipate her own request effort on the basis of the signal she receives about the territory. Thus, she accepts the contract if her expected utility exceeds her outside option.

When $\left(e^{\beta_{L}},e^{\beta_{H}})=(e_{H},e_{L}\right)$ can be ruled out (under the conditions shown in Table 2), the firm constructs the request mechanism such that the manager will make a request for relaxed constraints allowing $\left(e_{H},e_{H}\right)$ if and only if $\gamma_{L}<\gamma_{L}^{1}$ . When she makes that request, her expected utility is


$$E[U_{m}|\gamma_{L}<\gamma_{L}^{1}]=m_{L}+p_{H}\text{Δ}m-e_{m}^{r*}$$

$$=m_{L}+p_{H}\text{Δ}m-(1-\gamma_{L}^{1})(p_{H}-p_{0})\text{Δ}m$$

$$∴E[U_{m}|\gamma_{L}<\gamma_{L}^{1}]=m_{L}+\left(p_{0}+\gamma_{L}^{1}[p_{H}-p_{0}]\right)\text{Δ}m.$$

When she does not make a request, the baseline constraints allow her to induce $\left(e_{H},0\right)$ , so


$$E[U_{m}|\gamma_{L}>\gamma_{L}^{1}]=m_{L}+\left(p_{0}+\gamma_{L}[p_{H}-p_{0}]\right)\text{Δ}m.$$

Therefore, the manager’s expected utility is weakly increasing with her belief, $\gamma_{L}$ . A similar analysis shows that the same is true for the remaining case in which $\left(e_{H},e_{L}\right)$ is a viable option. Intuitively, this implies that the manager is always (weakly) better off when there is a greater likelihood that the territory is easy. Thus, if she accepts a given contract when $\gamma_{L}\to 0$ , she will accept it for any $\gamma_{L}$ .

### Firm’s Design of Manager’s Contract (Step 1)

When designing the manager’s contract, the firm cannot observe her belief about the territory type. The firm values the manager’s contributions (both modeled and unmodeled) enough that it seeks to ensure that she will accept her contract for any value of $\gamma_{L}$ . From Step 2, it is sufficient to ensure that she will accept it for $\gamma_{L}\to 0$ , so the firm maximizes its expected profit subject to that condition. The firm’s expected profit is based on the ex ante probability density function of the manager’s belief, which is denoted by $f(\gamma_{L})$ .

Suppose that, after the manager accepts her contract, the firm will set baseline constraints that allow $\left(e^{\beta_{L}},e^{\beta_{H}}\right)$ , relaxed constraints that allow (eβL',eβH') , and effort requirement $e_{m}^{r}$ such that the manager makes a request if and only if $\gamma_{L}$ is below some threshold $\gamma_{L}^{0}$ . The firm chooses the manager’s contract by solving the following:


maxmLE[π]=∫0γL0E[π(eβL',eβH')]f(γL)dγL+∫γL01E[π(eβL,eβH)]f(γL)dγLs.t. E[Um|γL→0]≥U¯m                                     (IRm)mL≥0                                                          (LLm),

where $\left({\text{LL}}_{m}\right)$ is the limited liability condition on the manager’s contract. For any $\left(e^{\beta_{L}},e^{\beta_{H}}\right)$ , Lemma 3 implies that


3 $$\begin{array}{l} E[\pi (e^{\beta_{L}},e^{\beta_{H}})]=x_{L}-m_{L}+\gamma_{L}[p(e^{\beta_{L}})(\text{Δ}x-\text{Δ}m)-(\bar{U}+\beta_{L}e^{\beta_{L}}+\text{Δ}\beta e^{\beta_{H}})]+(1-\gamma_{L})[p(e^{\beta_{H}})(\text{Δ}x-\text{Δ}m)-1_{e^{\beta_{H}}>0}(\bar{U}+\beta_{H}e^{\beta_{H}})],\; \end{array}$$

where the indicator function $1_{e^{\beta_{H}}>0}$ equals 0 if the salesperson exits and 1 if he stays.

I begin by solving an adapted version of the firm’s problem, without $\left({\text{LL}}_{m}\right)$ . In this case, it is straightforward to show that $\left({\text{IR}}_{m}\right)$ must bind; otherwise, the firm can increase its expected profit by lowering $m_{L}$ . Therefore, the firm sets $m_{L}$ as summarized in Table 3. Because ${\bar{U}}_{m}\geq p_{H}\text{Δ}m$ (by assumption), it is clear that $m_{L}>0$ in each case, so this is also a solution to the firm’s fully constrained optimization problem with $\left({\text{LL}}_{m}\right)$ included.

**Table 3. table3-0022243720969174:** Summary of Manager’s Contract.

**Case^a^**	**Manager’s Salary** $(m_{L})$ **^a^**
$(p_{L}-p_{0})(\text{Δ}x-\text{Δ}m)<\bar{U}+\beta_{H}e_{L}$ OR $\gamma_{L}^{0}<\gamma_{L}^{2}$	${\bar{U}}_{m}-\left(p_{0}+\gamma_{L}^{1}[p_{H}-p_{0}]\right)\text{Δ}m$
Otherwise	${\bar{U}}_{m}-\left(p_{0}+\gamma_{L}^{0}[p_{L}-p_{0}]+\gamma_{L}^{2}[p_{H}-p_{L}]\right)\text{Δ}m$

^a^From the proof of Proposition 2 in Web Appendix C:

$\gamma_{L}^{0}=\frac{\left(p_{L}-p_{0})(\text{Δ}x-\text{Δ}m)-(\bar{U}+\beta_{H}e_{L}\right)}{\left(p_{L}-p_{0})(\text{Δ}x-\text{Δ}m)-(\bar{U}+\beta_{L}e_{L}\right)}$

$\gamma_{L}^{1}=\frac{\left(p_{H}-p_{0})(\text{Δ}x-\text{Δ}m)-(\bar{U}+\beta_{H}e_{H}\right)}{\left(p_{H}-p_{0})(\text{Δ}x-\text{Δ}m)-(\bar{U}+\beta_{L}e_{H}\right)}$

$\gamma_{L}^{2}=\frac{(p_{H}-p_{L})(\text{Δ}x-\text{Δ}m)-\beta_{H}\text{Δ}e}{(p_{H}-p_{L})(\text{Δ}x-\text{Δ}m)-\beta_{L}\text{Δ}e}$

$\left({\text{IR}}_{m}\right)$ binding implies that the manager’s expected surplus is zero when $\gamma_{L}\to 0$ . From Step 2, the manager’s expected utility (weakly) increases with $\gamma_{L}$ . In other words, she earns information rent that (weakly) increases with $\gamma_{L}$ .

In summary, the proposed request mechanism provides a way for the firm to leverage a sales manager’s local information. The mechanism is always feasible, so it can be used to reveal this information under any conditions, thus preventing the manager from misrepresenting it for personal gain. However, implementing this mechanism is not costless. It requires the firm to pay information rent to the salesperson when his territory is easy and information rent to the manager that increases with the likelihood that the territory is easy $\left(\gamma_{L}\right)$ . Thus, it is worth comparing this request mechanism with a well-accepted solution from the literature.

### Comparison with Menu of Contracts

In the principal–agent literature, the standard approach to incentive design for an agent of unknown type is to offer a menu of contracts designed such that the agent will select the contract intended for his type. Notably, existing models assume that this menu is designed by a firm that has an informed belief about the agent’s type. In the context of my model, this would imply that the firm has costless access to the manager’s local information. In this section, I compare the proposed request mechanism to a menu of contracts designed by the firm without input from the manager. Because the menu of contracts is a well-studied approach, details of its solution are left to the appendices, and the focus here is on comparing the two mechanisms.

**Lemma 4:** Lemma 3 and Proposition 1 hold when “contract” is replaced with “menu of contracts.”

Lemma 4 (proof in Web Appendix E) indicates that, in this model setting, the efficient menu of contracts that will induce a given $\left(e^{\beta_{L}},e^{\beta_{H}}\right)$ is effectively equivalent to the efficient single contract (further discussed subsequently). Therefore, the firm faces a similar trade-off in both approaches, between effort induced if the territory is hard and information rent paid to the salesperson if it is easy. Thus, though the menu of contracts is designed to reveal a territory’s true type, the optimal menu design depends on the firm’s ex ante belief about that type. One implication of this trade-off, as noted in Lal and Staelin (1986), is that the firm might prefer $e^{\beta_{H}}=0$ even under a menu of contracts. In other words, the firm’s optimal menu does not necessarily contain a contract that the salesperson will accept if his territory is hard. In particular, this occurs when the likelihood of a hard territory (the proportion of “high performance salespeople” in Lal and Staelin [1986]) exceeds some threshold. In fact, all of the decision thresholds in my model (the values of $\gamma_{L}$ at which the firm’s preference switches from one effort combination $\left(e^{\beta_{L}},e^{\beta_{H}}\right)$ to another) are the same under the menu of contracts and the request mechanism.

The comparison of expected profits under the two mechanisms is summarized in Proposition 3.

**Proposition 3:** The request mechanism outperforms the menu of contracts when:

 (a) the firm’s best menu of contracts induces the salesperson to exit when his territory is hard
     OR
 (b) the manager’s incentive pay (Δm) is not too large.

The relative performance of the request mechanism increases as the firm’s decision threshold(s) approach its belief $\left(\gamma_{L}^{F}\right)$ about the territory type.

See Appendix D for a sketch proof and Web Appendix F for a detailed proof. The conditions under which the request mechanism outperforms the menu of contracts are summarized in Table 4.

**Table 4.** Conditions for Request Mechanism Outperforming Menu of Contracts.

**Case^a^**	**Request Mechanism Preferred When^a^:**
$(p_{L}-p_{0})(\text{Δ}x-\text{Δ}m)<\bar{U}+\beta_{H}e_{L}$ OR $\gamma_{L}^{0}<\gamma_{L}^{2}$	$\gamma_{L}^{1}<\gamma_{L}^{F}=\frac{1}{2}$ OR $\text{Δ}m<\frac{\text{Δ}\beta e_{H}}{2(p_{H}-p_{0})}$
Otherwise	$\gamma_{L}^{0}<\gamma_{L}^{F}=\frac{1}{2}$ OR $\left(\gamma_{L}^{2}<\gamma_{L}^{F}=\frac{1}{2}AND\text{Δ}m<\frac{(p_{H}-p_{L})\text{Δ}x-\beta_{H}\text{Δ}e+(1-\gamma_{L}^{0})\text{Δ}\beta e_{L}-\gamma_{L}^{2}\text{Δ}\beta \text{Δ}e}{2[(1-\gamma_{L}^{0})(p_{L}-p_{0})+(\frac{1}{2}-\gamma_{L}^{2})(p_{H}-p_{L})]}\right)$ OR $\text{Δ}m<\frac{(1-\gamma_{L}^{0})\text{Δ}\beta e_{L}+(1-\gamma_{L}^{2})\text{Δ}\beta \text{Δ}e}{2[(1-\gamma_{L}^{0})(p_{L}-p_{0})+(1-\gamma_{L}^{2})(p_{H}-p_{L})]}$

^a^From the proof of Proposition 2 in Web Appendix C:

$\gamma_{L}^{0}=\frac{\left(p_{L}-p_{0})(\text{Δ}x-\text{Δ}m)-(\bar{U}+\beta_{H}e_{L}\right)}{\left(p_{L}-p_{0})(\text{Δ}x-\text{Δ}m)-(\bar{U}+\beta_{L}e_{L}\right)}$

$\gamma_{L}^{1}=\frac{\left(p_{H}-p_{0})(\text{Δ}x-\text{Δ}m)-(\bar{U}+\beta_{H}e_{H}\right)}{\left(p_{H}-p_{0})(\text{Δ}x-\text{Δ}m)-(\bar{U}+\beta_{L}e_{H}\right)}$

$\gamma_{L}^{2}=\frac{(p_{H}-p_{L})(\text{Δ}x-\text{Δ}m)-\beta_{H}\text{Δ}e}{(p_{H}-p_{L})(\text{Δ}x-\text{Δ}m)-\beta_{L}\text{Δ}e}$

The results of this comparison reflect the balance between two factors under each mechanism. The first is the optimality of the salesperson’s effort. The effort combination $\left(e^{\beta_{L}},e^{\beta_{H}}\right)$ induced under the request mechanism is determined by the manager’s belief $\left(\gamma_{L}\right)$ about the territory type. The combination induced under the menu of contracts is based on the firm’s belief $\left(\gamma_{L}^{F}\right)$ without the manager’s information. Because the manager’s belief is more informed than the firm’s, the request mechanism results in a better choice of $\left(e^{\beta_{L}},e^{\beta_{H}}\right)$ than the menu. In other words, the request mechanism always offers an informational advantage in optimizing the salesperson’s effort. Consider the case in which the firm can narrow its decision to two options: $\left(e^{\beta_{L}},e^{\beta_{H}})=(e_{H},0\right)$ or $\left(e_{H},e_{H}\right)$ . From Proposition 1 and Lemma 4, the firm prefers the latter under either mechanism only if the likelihood that the territory is easy falls below the decision threshold $\gamma_{L}^{1}$ . Now suppose $\gamma_{L}^{1}=40\%$ . By the firm’s uninformed belief, $\gamma_{L}^{F}=50\%$ , which exceeds the 40% threshold. Therefore, the firm chooses a menu of contracts that induces $\left(e_{H},0\right)$ . If the manager’s belief is below the threshold ( $\gamma_{L}<40\%$ ), the request mechanism results in $\left(e_{H},e_{H}\right)$ , which is of greater benefit to the firm than $\left(e_{H},0\right)$ . It is clear that the likelihood of such a reversal and, therefore, the expected benefit of the request mechanism increases as the decision threshold approaches the firm’s belief (as $\gamma_{L}^{1}\to \gamma_{L}^{F}$ in this example).

The second factor is the efficiency of the manager’s contract, which can favor either approach. Suppose the optimal menu of contracts induces the salesperson to exit when his territory is hard (i.e., $\left(e^{\beta_{L}},e^{\beta_{H}})=(e_{H},0\right)$ ). Under that mechanism, the firm must pay the manager a large enough salary to ensure that she will accept her own contract when the probability of earning her bonus (Δm) is only $p_{0}$ . Under the request mechanism, on the other hand, the manager will request relaxed constraints if she believes the territory is likely to be hard, allowing her to offer a contract that the salesperson will accept regardless of territory type. This increases the manager’s likelihood of earning her bonus and allows the firm to offer her a more efficient contract. That increased efficiency adds to the informational advantage of the request mechanism, so it clearly outperforms the menu of contracts when the optimal menu induces the salesperson to exit a hard territory. However, if the firm’s optimal menu induces $\left(e_{H},e_{H}\right)$ , the reverse is true. The salesperson will always exert high effort, so the firm can offer the manager a more efficient contract under that menu than it can under the request mechanism. Furthermore, that efficiency gain increases with the size of the manager’s bonus (Δm) . This offsets the request mechanism’s informational advantage, so the request mechanism is preferred only as long as Δm is not too large.

**Figure 1.**
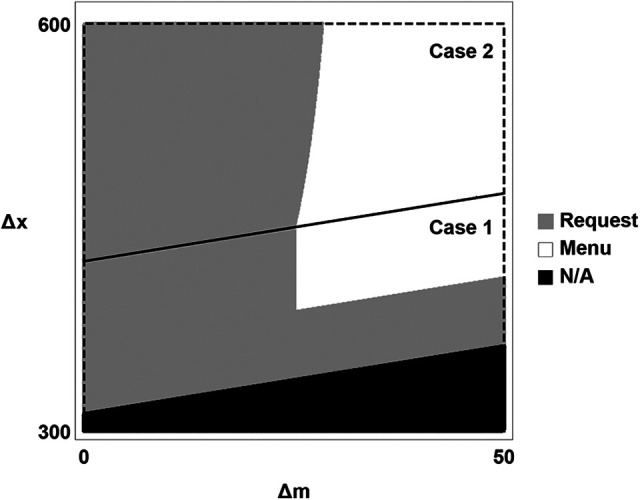
Illustration of preferred mechanisms. Note: $p_{0}=20\%$ , $p_{L}=50\%$ , $p_{H}=60\%$ , $e_{L}=10$ , $e_{H}=20$ , $\beta_{L}=1$ , $\beta_{H}=2$ , $\bar{U}=85$ , ${\bar{U}}_{m}=90$

Figure 1 illustrates the conditions under which the firm prefers the request mechanism to the menu of contracts for a given set of parameters. In the gray region, the firm prefers the request mechanism. The gray section on the left corresponds to part (b) of Proposition 3 ( Δm not too large) and the gray section along the bottom corresponds to part (a) (the best menu induces exit from a hard territory). The black region lies outside the parameter space of the model since the firm would prefer not to employ the salesperson if it knew his territory to be hard. In that case, no information revelation mechanism is necessary—the firm simply offers its optimal contract for an easy territory and the salesperson exits if his territory is hard. In general, the shapes of these regions are roughly similar for any set of parameters that meets the model criteria, so the menu of contracts is preferred only when both the manager’s incentive pay (Δm) and the firm’s incremental profit from high sales net of that incentive pay (Δx−Δm) are sufficiently high.

It is worth highlighting the extent to which the preceding results rely on certain assumptions. First, as previously noted, Lemma 4 implies that a menu of contracts is no more efficient than a single contract to induce a given effort combination $\left(e^{\beta_{L}},e^{\beta_{H}}\right)$ . This, in turn, suggests that the request mechanism can fully replace and improve on a menu of contracts under the conditions in Proposition 3. This finding relies, in part, on the assumption that the salesperson is risk neutral. If he is risk averse, a single contract can no longer match the efficiency of a menu. Thus, the conditions under which the request mechanism outperforms a menu of contracts are similar but more restrictive for a risk averse salesperson. However, the conditions for a risk neutral salesperson (as given in Proposition 3) do apply for a risk averse salesperson if the request mechanism is combined with a menu of contracts. To do this, the firm delegates the salesperson’s compensation design to the manager but allows her to offer a menu of contracts instead of a single contract. The request mechanism remains essentially the same: The firm imposes constraints to ensure that the manager offers the salesperson an efficient menu to induce some $\left(e^{\beta_{L}},e^{\beta_{H}}\right)$ and allows her to make a costly request to relax those constraints. This combined approach offers the benefits of both methods, allowing the firm to leverage both the manager’s more complete information and the greater efficiency of the menu (relative to a single contract) when the salesperson is risk averse.

Second, the comparison of the request and menu mechanisms is based on a sequence in which the firm commits to the manager’s contract before observing the salesperson’s choice from the menu of contracts (i.e., before he reveals his territory type). Though this is the most likely sequence, the reverse is also possible. If the firm observes the salesperson’s choice first, it can avoid paying information rent to the manager. Thus, her contract would be more efficient under a menu than under the request mechanism. In that case, part (a) of Proposition 3 does not always hold. Part (b) continues to hold in that the request mechanism still outperforms the menu when Δm is not too large, although the threshold in this case is lower than the one in the proposed model. The final part of the proposition (the benefit of the request mechanism increasing as the decision thresholds approach $\gamma_{L}^{F}$ ) continues to hold for much of the parameter space but not all of it.^9^

### Numerical Example

The following numerical example illustrates the request mechanism and how its results compare with those of a menu of contracts. Consider a firm employing a salesperson supervised by a sales manager. Sales in the salesperson’s territory are either low $\left(x_{L}=100\right)$ or high $\left(x_{H}=400\right)$ . If the salesperson exerts low effort $\left(e_{L}=15\right)$ , the probability of high sales is $p_{L}=45\%$ . If he exerts high effort $\left(e_{H}=20\right)$ , that probability increases to $p_{H}=60\%$ . If the salesperson exits, high sales occur with probability $p_{0}=20\%$ . If the territory is easy, the salesperson’s effort cost is equal to his effort ( $e_{L}$ or $e_{H}$ ). If the territory is hard, his effort cost doubles ( $\beta_{L}=1,\beta_{H}=2$ ). If the salesperson (manager) exits the firm, he (she) receives outside option utility $\bar{U}=40$ ( ${\bar{U}}_{m}=50$ ). The manager’s incentive pay is set exogenously by the firm at Δm=60 .


xL=100   p0=.2   βL=1xH=400 pL=.45 βH=2eL=15 pH=.6 U¯=40eH=20 Δm=60 U¯m=50

For the firm, the incremental value of inducing high versus low effort is $(p_{H}-p_{L})(\text{Δ}x-\text{Δ}m)=36$ , which exceeds the salesperson’s cost of that incremental effort in an easy territory $\left(\beta_{L}\text{Δ}e=5\right)$ . Thus, as in Proposition 1, the firm always prefers to induce high effort in an easy territory. In a hard territory, the incremental value of inducing low effort versus letting the salesperson exit is $(p_{L}-p_{0})(\text{Δ}x-\text{Δ}m)=60$ , which is less than the total cost of employing him to exert that effort, $\bar{U}+\beta_{H}e_{L}=70$ . Furthermore, letting him exit a hard territory allows the firm (or manager) to optimize his compensation when the territory is easy. Thus, the firm never prefers to induce low effort in a hard territory, so the menu of contracts and the request mechanism result in either high effort or exit $\left(e^{\beta_{H}}\in \{0,20\}\right)$ . This case corresponds to the first row of Tables 2 [Table table3-0022243720969174]–4.

First, consider the menu of contracts approach. As noted following Proposition 2, the firm prefers to induce $e^{\beta_{H}}=20$ if and only if its belief about the territory type is below the threshold $\gamma_{L}^{1}=\frac{\left(p_{H}-p_{0})(\text{Δ}x-\text{Δ}m)-(\bar{U}+\beta_{H}e_{H}\right)}{\left(p_{H}-p_{0})(\text{Δ}x-\text{Δ}m)-(\bar{U}+\beta_{L}e_{H}\right)}=.44$ . Because the firm’s uninformed belief is $\gamma_{L}^{F}=.5$ , it chooses instead to let the salesperson exit a hard territory by offering a “menu” consisting solely of the optimal contract that induces high effort when the territory is easy, $\left(s_{L},s_{H})=(40,73.3\right)$ (see Web Appendix E). If the territory is hard, the salesperson will reject that contract and exit. To ensure that the manager will accept her contract even when she believes that the territory is likely to be hard, the firm offers her a salary of $m_{L}={\bar{U}}_{m}-p_{0}\text{Δ}m=38$ (see Web Appendix F). Thus, the firm’s expected profit is $\text{E[}\pi \text{|menu]}=\gamma_{L}^{F}(x_{L}-s_{L}-m_{L}+p_{H}[\text{Δ}x-\text{Δ}s-\text{Δ}m])+(1-\gamma_{L}^{F})(x_{L}-m_{L}+p_{0}[\text{Δ}x-\text{Δ}m])=128$ .

Next, consider the request mechanism. From Step 5, when the firm imposes no constraints, the manager will choose to induce high effort in both territory types, which she can do efficiently by offering $\left(s_{L},s_{H})=(40,106.7\right)$ (see Web Appendix B). The firm can ensure that she induces $\left(e_{H},0\right)$ instead by imposing a total payout cap of $\hat{s_{H}}=73.3$ and a salary cap of $\hat{s_{L}}=40$ (see Appendix A). From Step 3, the firm imposes these constraints but allows the manager to exert effort $e_{m}^{r*}=(1-\gamma_{L}^{1})(p_{H}-p_{0})\text{Δ}m=13.3$ to have them removed. From Table 3, the firm offers the manager a salary of $m_{L}={\bar{U}}_{m}-(p_{0}+\gamma_{L}^{1}[p_{H}-p_{0}])\text{Δ}m=27.3$ .

If the manager makes a request, her expected utility is $E[U^{m}|request]=m_{L}+p_{H}\text{Δ}m-e_{m}^{r*}=50$ . If she does not make a request, her expected utility is $E[U^{m}|norequest]=m_{L}+\left(p_{0}+\gamma_{L}[p_{H}-p_{0}]\right)\text{Δ}m=39.3+24\gamma_{L}$ . Therefore, she makes a request if and only if her belief $\gamma_{L}<\frac{50-39.3}{24}=0.44=\gamma_{L}^{1}$ . That is, she chooses to induce the salesperson to exert high effort in a hard territory precisely when the firm prefers it. From Appendix D, the firm’s expected profit is $\text{E[}\pi \text{|req]}=x_{L}+p_{0}\text{Δ}x-{\bar{U}}_{m}+\gamma_{L}^{1}\left[\left(p_{H}-p_{0})\text{Δ}x-(\bar{U}+\beta_{H}e_{H}\right)\right]+(1-\gamma_{L}^{1})\left(\frac{1+\gamma_{L}^{1}}{2}\left[\left(p_{H}-p_{0})(\text{Δ}x-\text{Δ}m)-(\bar{U}+\beta_{L}e_{H}\right)\right]\right)=142.2$ .

In this case, then, the firm is better off using the request mechanism than a menu of contracts. It benefits from the manager’s ability to induce the salesperson to stay and exert high effort when her (more informed) belief indicates that the territory is sufficiently likely to be hard. Furthermore, the request mechanism allows the firm to reduce the manager’s salary because she also benefits when the salesperson stays. In Figure 1, this case would fall in the gray band along the bottom.

For comparison, now suppose that the manager’s incentive pay is Δm=40 instead of 60 and that all other parameters are unchanged. This reduction increases the firm’s net benefit from high effort (Δx−Δm) such that its preferred menu of contracts now induces high effort even when the territory is hard because $\gamma_{L}^{1}=.55>\gamma_{L}^{F}$ . The firm offers the manager a salary of 26 and earns an expected profit of 150. Under the request mechanism, the manager’s salary is 33.3, the required request effort is 7.3, and the firm’s expected profit is 147.3. The request mechanism still allows the firm to benefit from the manager’s information by inducing the salesperson to exit when his exit is best for the firm, but it must pay the manager a higher salary to make up for her loss in expected incentive pay when that happens. In this case, the salary increase outweighs the value of her information, so the firm prefers the menu of contracts. The decrease in Δm increases Δx−Δm enough to move this case out of the gray band in Figure 1 and into the white area.

Finally, suppose that the manager’s incentive pay is even smaller, at Δm=20 . Again, the firm’s preferred menu of contracts always induces high effort, so its expected profit remains at 150. The manager’s salary increases to 38 to balance her lower incentive pay. Under the request mechanism, the manager’s salary is 41.1, her required request effort is 3.1, and the firm’s expected profit is 150.8. Thus, the value of the manager’s information outweighs the cost of her higher salary and the firm prefers the request mechanism. This case falls in the gray area on the left side of Figure 1.

## Model Extensions

I consider two extensions of the main model. In the first, I incorporate a broader range of sales manager responsibilities, which allows me to fully endogenize her compensation plan. This analysis shows how the firm can optimize her contract while including her in designing the salesperson’s compensation. In the second extension, the manager can acquire better information about the salesperson’s territory (at a cost) prior to being involved in designing the salesperson’s contract.

### Extension 1: Full Optimization of the Manager’s Contract

In the main model, I focus solely on the manager’s involvement in designing the salesperson’s compensation plan. As a result, her incentive pay (Δm) has to be exogenous since, in the absence of other activities (particularly ones that affect sales), the firm would optimally set Δm=0 , thereby eliminating the conflict of interest that discourages the manager from sharing her information freely and negating the need for the request mechanism.

In practice, of course, sales managers perform a range of tasks that have direct or indirect impacts on sales, and firms typically use sales-based incentives to motivate managers’ efforts. Furthermore, Proposition 3 indicates that the magnitude of the incentives plays an important role in determining whether the firm should involve the manager in designing the salesperson’s compensation. Thus, I endogenize and optimize the manager’s incentive pay in this extension, which requires extending the model to capture her effort on tasks that affect sales. For example, a sales manager can impact sales directly by joining the salesperson in discussions with important customers and indirectly by improving the salesperson’s effectiveness through coaching and training. There is little research on this type of sales support effort and its interaction with salespeople’s effort. Because this is not the main focus of this research, I employ a simple model formulation that emphasizes parsimony and tractability. More extensive modeling of sales managers’ activities and their impacts is left to future research.

The manager’s support effort takes one of two values, $e_{m}^{s}\in \{0,e_{m}^{s*}\}$ , where $e_{m}^{s*}>0$ . This effort has a simple additive effect of $p_{m}(e_{m}^{s})$ on the probability of the high sales outcome; therefore, if the salesperson exerts effort e , then $\text{Pr}(x=x_{H})=p(e)+p_{m}(e_{m}^{s})$ . If the manager exerts no support effort, the outcome is unaffected (i.e., $p_{m}(0)=0$ ), and there remains uncertainty in the outcome regardless of her effort, so $p_{H}+p_{m}(e_{m}^{s*})<1$ . The manager’s disutility from effort is additive, so her total effort cost is $v(e_{m}^{r},e_{m}^{s})=e_{m}^{r}+e_{m}^{s}$ .

To make the cleanest comparison between mechanisms, I restrict attention to the case in which the firm always prefers to employ the sales manager and incentivize her to exert support effort regardless of whether the firm uses her information to design the salesperson’s contract. Specifically, $p_{m}(e_{m}^{s*})\text{Δ}x>e_{m}^{s*}+{\bar{U}}_{m}+(p_{H}-p_{0})\frac{e_{m}^{s*}}{p_{m}(e_{m}^{s*})}$ . I assume that the salesperson observes the manager’s support effort, although the results are unchanged if he does not (as he can infer it by observing her contract or from the fact that the firm always prefers to induce $e_{m}^{s*}$ ).

The remaining assumptions are the same as those in the main model. The conditions ensuring that the limited liability constraints do not drive the outcome are $\bar{U}\geq \frac{\beta_{H}}{p_{H}-p_{L}}(p_{L}e_{H}-p_{H}e_{L}+p_{m}(e_{m}^{s*})\text{Δ}e)\geq 0$ and ${\bar{U}}_{m}\geq p_{H}\frac{e_{m}^{s*}}{p_{m}(e_{m}^{s*})}$ . The sequence of events is unchanged with two exceptions: (1) Rather than determining Δm in Step 0, the firm sets both $m_{L}$ and Δm in Step 1, and (2) in Step 5, the manager chooses her support effort $\left(e_{m}^{s}\right)$ in addition to announcing the salesperson’s contract.

Analysis of this extension supports the robustness of the findings from the main model. The request mechanism remains feasible and outperforms a menu of contracts under the same conditions.

**Proposition 4:** Lemmas 1–4 and Propositions 1–3 continue to hold when the manager’s incentive pay is endogenized. The results in Tables 2 [Table table3-0022243720969174]–4 remain the same with $\text{Δ}m=\text{Δ}m^{*}\equiv \frac{e_{m}^{s*}}{p_{m}(e_{m}^{s*})}$ .

The analysis and intuition closely follow those from the main model (see complete details in Web Appendix G). As with the salesperson’s incentive pay, the optimal incentive pay for the manager $\left(\text{Δ}m^{*}\right)$ is determined by the ratio of the cost of her support effort to the impact of that effort on sales. By extension of Proposition 3, the firm prefers to involve the manager in the salesperson’s compensation design (through the request mechanism) when that ratio is sufficiently low or when the firm would otherwise induce the salesperson in a hard territory to exit. Thus, the general shape of Figure 1 continues to apply, with the horizontal axis representing $\frac{e_{m}^{s*}}{p_{m}(e_{m}^{s*})}$ .

### Extension 2: Manager Can Obtain Additional Information

In the main model, the manager acquires territory information passively in the course of fulfilling her responsibilities (e.g., coaching the salesperson, meeting with customers). As a result, the signal she receives is effectively costless but is imperfect. In this extension, the manager can incur a cost to improve her information before deciding whether to make a request for relaxed constraints. For example, a manager can obtain better information by investing additional time (beyond her regular duties) to speak with a salesperson, navigate the salesperson’s territory, visit customers, or assess the sales pipeline. For simplicity, I assume that this investment results in a perfect signal ( Q=1 ).

The manager’s willingness to acquire information is driven by the potential benefit it provides. In particular, she benefits when the acquired information results in a different action than the one she would have taken without it. For example, she initially prefers to request relaxed constraints if her signal indicates that the territory is sufficiently likely to be hard. Additional information would then add value if it indicates that the territory is easy, leading her to change her decision and save the effort cost of the request. Conversely, if she initially prefers not to make a request, then additional information adds value if it indicates that the territory is hard. In this case, she would make a request and thus would benefit from the salesperson’s greater effort. As in Proposition 3, these reversals are most likely to occur when the manager’s belief $\gamma_{L}$ is close to a decision threshold, which leads to the following:

**Proposition 5:** If the cost to acquire additional information is not too high, the manager acquires it when her belief about the territory type is sufficiently close to a decision threshold.

See Web Appendix H for a detailed proof. The conditions under which the manager acquires additional information are summarized in Table 5, with C representing the cost of acquisition. Naturally, when the cost is very high, the manager never chooses to incur it. Conversely, when the cost is very low, she always acquires information (i.e., “sufficiently close” includes all values of $\gamma_{L}$ ). When the cost is moderate, her decision depends on her initial belief.

**Table 5.** Summary of Information Acquisition Conditions.

**Case^a^**	**Manager Acquires Info If^a^:**
( $(p_{L}-p_{0})(\text{Δ}x-\text{Δ}m)<\bar{U}+\beta_{H}e_{L}$ OR $\gamma_{L}^{0}<\gamma_{L}^{2}$ ) AND $\gamma_{L}>\gamma_{L}^{1}$	$\gamma_{L}^{1}(1-\gamma_{L})(p_{H}-p_{0})\text{Δ}m>C$
( $(p_{L}-p_{0})(\text{Δ}x-\text{Δ}m)<\bar{U}+\beta_{H}e_{L}$ OR $\gamma_{L}^{0}<\gamma_{L}^{2}$ ) AND $\gamma_{L}<\gamma_{L}^{1}$	$\gamma_{L}(1-\gamma_{L}^{1})(p_{H}-p_{0})\text{Δ}m>C$
$(p_{L}-p_{0})(\text{Δ}x-\text{Δ}m)>\bar{U}+\beta_{H}e_{L}$ AND $\gamma_{L}>\gamma_{L}^{0}>\gamma_{L}^{2}$	$(1-\gamma_{L})[\gamma_{L}^{0}(p_{L}-p_{0})+\gamma_{L}^{2}(p_{H}-p_{L})]\text{Δ}m>C$
$(p_{L}-p_{0})(\text{Δ}x-\text{Δ}m)>\bar{U}+\beta_{H}e_{L}$ AND $\gamma_{L}^{0}>\gamma_{L}>\gamma_{L}^{2}$	$[\gamma_{L}(1-\gamma_{L}^{0})(p_{L}-p_{0})+\gamma_{L}^{2}(1-\gamma_{L})(p_{H}-p_{L})]\text{Δ}m>C$
$(p_{L}-p_{0})(\text{Δ}x-\text{Δ}m)>\bar{U}+\beta_{H}e_{L}$ AND $\gamma_{L}^{0}>\gamma_{L}^{2}>\gamma_{L}$	$\gamma_{L}[(1-\gamma_{L}^{0})(p_{L}-p_{0})+(1-\gamma_{L}^{2})(p_{H}-p_{L})]\text{Δ}m>C$

^a^From the proof of Proposition 2 in Web Appendix C:

$\gamma_{L}^{0}=\frac{\left(p_{L}-p_{0})(\text{Δ}x-\text{Δ}m)-(\bar{U}+\beta_{H}e_{L}\right)}{\left(p_{L}-p_{0})(\text{Δ}x-\text{Δ}m)-(\bar{U}+\beta_{L}e_{L}\right)}$

$\gamma_{L}^{1}=\frac{\left(p_{H}-p_{0})(\text{Δ}x-\text{Δ}m)-(\bar{U}+\beta_{H}e_{H}\right)}{\left(p_{H}-p_{0})(\text{Δ}x-\text{Δ}m)-(\bar{U}+\beta_{L}e_{H}\right)}$

$\gamma_{L}^{2}=\frac{(p_{H}-p_{L})(\text{Δ}x-\text{Δ}m)-\beta_{H}\text{Δ}e}{(p_{H}-p_{L})(\text{Δ}x-\text{Δ}m)-\beta_{L}\text{Δ}e}$

The firm naturally prefers the manager to always acquire the best possible information. However, the firm benefits from that acquisition only when it changes the manager’s resulting action. In fact, because the request mechanism effectively aligns the manager’s interests with those of the firm, the manager acquires information not only when her own expected benefit is highest but when the firm’s is highest as well.

Proposition 5 is based on a simple model extension in which the manager’s cost of acquiring information is fixed, but the finding is largely robust to relaxing that assumption. In particular, the information acquisition cost might be inversely proportional to the quality ( Q ) of the manager’s initial signal. This would be the case if, for example, the initial signal quality reflects the ease of assessing the territory type or the amount of information already collected. In such cases, the manager still benefits most from acquiring additional information when her belief is close to a decision threshold. However, she is more likely to invest in additional information when her initial belief is stronger (closer to 0 or 1) because the cost of improving a strong signal is low. This response deviates somewhat from the firm’s priorities, which are better aligned with the manager’s decisions when the cost of additional information is fixed.

## Conclusions

Sales managers make critical contributions to their organizations, including hiring, training, and coaching salespeople, contributing to territory and compensation design, and assisting with key customer relationships. A common thread in many of these contributions is the manager’s role as a conduit of information between the firm and the sales force. This role is often overlooked in the literature, with models treating sales managers as interchangeable with their firms or omitting them entirely. In reality, a manager’s information and objectives are distinct from those of the firm, which often leads to challenging internal dynamics that prior studies have not fully captured.

This article examines the role of sales managers in designing sales force compensation plans. Optimal compensation plan design requires the best possible local information, and the most informed individuals after salespeople typically are first-line sales managers. However, managers often have an incentive to misrepresent that information because their compensation is based on the output of their salespeople. As a result, firms use formal and informal processes to access managers’ information and limit their potential to bias results. I propose a mechanism by which a firm can reliably and efficiently induce a sales manager to use her information to benefit the firm. Under this “request mechanism,” the firm delegates the design of sales incentives to the manager subject to tight constraints and offers an opportunity for her to request relaxed constraints by meeting specified requirements.

I find that such constraints and request requirements can always be designed such that the manager chooses the best possible incentive plan for the firm given her private information. Furthermore, when the firm is less informed than the manager, the request mechanism can outperform the well-established menu of contracts approach to incentive design. In particular, the proposed mechanism increases the firm’s expected profit when (1) the firm’s best possible menu of contracts results in some types of salesperson exiting and/or (2) the manager’s incentive pay is not too large. Finally, I show that the request mechanism can entice the manager to invest in acquiring additional information when it most benefits both her and the firm.

This article illustrates the value of involving sales managers in sales force compensation design, particularly when the firm establishes a well-designed process with clearly defined conditions and outcomes. The proposed mechanism is a simple, transparent way to prevent managers from seeking more-relaxed constraints (e.g., larger budgets) than necessary, also known as “sandbagging.” It can not only outperform the standard theoretical solution but it also offers an efficient, objective, and reliable alternative to commonly observed processes such as internal (e.g., top-down/bottom-up) negotiations and behind-the-scenes lobbying to determine commissions and quotas.

Although the stylized model used here refers to a general contract (salary + bonus) and corresponding constraints, the results can be applied quite broadly. For example, the “relaxed constraints” that the manager requests could represent lower quotas or higher commission rates. Furthermore, firms can leverage elements of their existing processes when implementing the proposed approach. For example, top-down and bottom-up processes for identifying territory potential can be used to identify the range of possible “types” of territories (or salespeople). Instead of using subjective negotiations to determine the true type, the firm should clearly specify what is required of a sales manager to specify a territory’s type and the implications of that specification (i.e., the resulting restrictions or parameters for the salesperson’s incentive plan). This article identifies the level of effort the manager should be required to exert; however, firm judgment and likely some trial and error will be required to translate that theoretical value into specific tasks or requirements.

This article is the first of its kind to consider the distinct role of the sales manager in sales force compensation design and points to potential directions for future research. For example, I focus here on a single manager and salesperson. The request mechanism and key findings can easily be extended to a manager overseeing multiple salespeople if the firm imposes constraints (and implements the mechanism) for each one individually. However, future research might explore whether the mechanism can be made even more efficient by applying budget constraints to the manager’s entire span of control, allowing her to pool and apply her information across territories.

Future research could also consider other mechanisms for leveraging a sales manager’s private information, such as tying her budget constraints to her compensation. For example, the firm could offer the manager a menu of contracts in which more-favorable compensation terms come with tighter constraints on the salesperson’s compensation. Alternatively, the manager’s contract could include a bonus for using less than her entire budget.

Finally, this article focuses primarily on the manager’s role in designing sales force compensation, but it is worth considering how this task interacts with her other responsibilities. For example, do her incentive design decisions affect the amount of time and effort she spends coaching particular salespeople (or recruiting and hiring, etc.) or vice versa? Would the proposed request mechanism affect her decisions in other areas such as territory design? In the first model extension, I use a simple additive model to represent the effect of the manager’s “support effort” on sales. Future research could consider alternative models that allow for more complex interactions between the manager’s and salesperson’s efforts.

## Supplemental Material

Supplemental Material, Involving_Sls_Mgrs_in_SF_Incentive_Design_v9.0_JMR_revision_4_(Web_Appendix) - Involving Sales Managers in Sales Force Compensation Design Supplemental Material, Involving_Sls_Mgrs_in_SF_Incentive_Design_v9.0_JMR_revision_4_(Web_Appendix) for Involving Sales Managers in Sales Force Compensation Design by Rob Waiser in Journal of Marketing Research

## Appendix A: Summary of Contract Constraints

No constraints are necessary to induce $\left(e_{H},e_{H}\right)$ . The manager is indifferent between choices of $\left(s_{L},s_{H}\right)$ that induce the same $\left(e^{\beta_{L}},e^{\beta_{H}}\right)$ ), so she chooses an efficient contract, by assumption.

To induce $\left(e_{H},e_{L}\right)$ , the firm can impose ${\hat{s_{H}}}^{\left(e_{H},e_{L}\right)}=\bar{U}+\beta_{H}e_{L}+\frac{(1-p_{L})\beta_{L}\text{Δ}e}{p_{H}-p_{L}}$ .

To induce $\left(e_{H},0\right)$ , it can impose $\left({\hat{s_{L}}}^{\left(e_{H},0\right)},{\hat{s_{H}}}^{\left(e_{H},0\right)})=(\bar{U}+\beta_{L}e_{H}-\frac{p_{H}\beta_{L}\text{Δ}e}{p_{H}-p_{L}},\bar{U}+\beta_{L}e_{H}+\frac{(1-p_{H})\beta_{L}\text{Δ}e}{p_{H}-p_{L}}\right)$ .

To induce $\left(e_{L},e_{L}\right)$ , it can impose $\left({\hat{\text{Δ}s}}^{\left(e_{L},e_{L}\right)},{\hat{s_{H}}}^{\left(e_{L},e_{L}\right)})=(0,\bar{U}+\beta_{H}e_{L}\right)$ .

To induce $\left(e_{L},0\right)$ , it can impose ${\hat{s_{H}}}^{\left(e_{L},0\right)}=\bar{U}+\beta_{L}e_{L}$ .

Trivially, it can induce (0,0) by imposing ${\hat{s_{H}}}^{\left(0,0\right)}=0$ .

## Appendix B: Proof of Proposition 1

By Lemma 3, an efficient contract offers the salesperson an expected surplus of 0 if the territory is hard and $\text{Δ}\beta e^{\beta_{H}}$ if it is easy. So, under an efficient contract that induces positive $\left(e^{\beta_{L}},e^{\beta_{H}}\right)$ ,

$$s_{L}+p(e^{\beta_{H}})\text{Δ}s=\bar{U}+\beta_{H}e^{\beta_{H}}\text{(for }e^{\beta_{H}}>0\text{) and }s_{L}+p(e^{\beta_{L}})\text{Δ}s=\bar{U}+\beta_{L}e^{\beta_{L}}+\text{Δ}\beta e^{\beta_{H}}.$$

Furthermore, when the firm chooses the constraints to impose on the salesperson’s contract (effectively choosing his effort in each territory type), the manager’s contract is already fixed.

Therefore, given belief $\gamma_{L}$ about the probability of an easy territory, the firm’s expected profit under each feasible effort combination $\left(e^{\beta_{L}},e^{\beta_{H}}\right)$ is as follows:

$$E[\pi (e_{L},0)]=x_{L}-m_{L}+(p_{0}+\gamma_{L}[p_{L}-p_{0}])(\text{Δ}x-\text{Δ}m)-\gamma_{L}(\bar{U}+\beta_{L}e_{L})$$

$$E[\pi (e_{L},e_{L})]=x_{L}-m_{L}+p_{L}(\text{Δ}x-\text{Δ}m)-(\bar{U}+\beta_{H}e_{L})$$

$$E[\pi (e_{H},0)]=x_{L}-m_{L}+(p_{0}+\gamma_{L}[p_{H}-p_{0}])(\text{Δ}x-\text{Δ}m)-\gamma_{L}(\bar{U}+\beta_{L}e_{H})$$

$$E[\pi (e_{H},e_{L})]=x_{L}-m_{L}+(p_{L}+\gamma_{L}[p_{H}-p_{L}])(\text{Δ}x-\text{Δ}m)-(\bar{U}+\beta_{H}e_{L}+\gamma_{L}\beta_{L}\text{Δ}e)$$

$$E[\pi (e_{H},e_{H})]=x_{L}-m_{L}+p_{H}(\text{Δ}x-\text{Δ}m)-(\bar{U}+\beta_{H}e_{H})$$

Claim: If the territory is easy, the effort induced is independent of $\gamma_{L}$ .

Proof: Comparing the previous equations gives:

4
$$E[\pi (e_{L},0)]-E[\pi (e_{H},0)]=\gamma_{L}[\beta_{L}\text{Δ}e-(p_{H}-p_{L})(\text{Δ}x-\text{Δ}m)]$$

5
$$E[\pi (e_{L},e_{L})]-E[\pi (e_{H},e_{L})]=\gamma_{L}[\beta_{L}\text{Δ}e-(p_{H}-p_{L})(\text{Δ}x-\text{Δ}m)]$$

6
$$E[\pi (e_{L},e_{L})]-E[\pi (e_{H},e_{H})]=\beta_{H}\text{Δ}e-(p_{H}-p_{L})(\text{Δ}x-\text{Δ}m)$$

It is clear that a contract inducing $e^{\beta_{L}}=e_{L}$ can be best if and only if $(p_{H}-p_{L})(\text{Δ}x-\text{Δ}m)<\beta_{L}\text{Δ}e$ . Otherwise, the firm’s best contract must induce either $\left(e_{H},0\right)$ , $\left(e_{H},e_{L}\right)$ , or $\left(e_{H},e_{H}\right)$ .

Therefore, the effort induced if the territory is easy $\left(e^{\beta_{L}}\right)$ is independent of $\gamma_{L}$ .

Claim: $(p_{H}-p_{L})(\text{Δ}x-\text{Δ}m)>\beta_{H}\text{Δ}e$

Proof: By assumption, the firm prefers to employ the salesperson (over letting him exit) even when the territory is known to be hard. This implies that at least one of the following must hold:

7
$$(p_{L}-p_{0})(\text{Δ}x-\text{Δ}m)>\bar{U}+\beta_{H}e_{L}$$

8
$$(p_{H}-p_{0})(\text{Δ}x-\text{Δ}m)>\bar{U}+\beta_{H}e_{H}$$

By assumption, $\bar{U}\geq \beta_{H}(\frac{p_{L}e_{H}-p_{H}e_{L}}{p_{H}-p_{L}})$ . Substituting this into Equations 7 and 8 gives

$$(p_{L}-p_{0})(\text{Δ}x-\text{Δ}m)>\beta_{H}(\frac{p_{L}e_{H}-p_{H}e_{L}}{p_{H}-p_{L}})+\beta_{H}e_{L}$$

$$∴(p_{L}-p_{0})(\text{Δ}x-\text{Δ}m)>\frac{\beta_{H}p_{L}\text{Δ}e}{p_{H}-p_{L}}$$

$$∴(p_{H}-p_{L})(\text{Δ}x-\text{Δ}m)>\frac{\beta_{H}p_{L}\text{Δ}e}{p_{L}-p_{0}},$$

and similarly, $\;(p_{H}-p_{L})(\text{Δ}x-\text{Δ}m)>\frac{\beta_{H}p_{H}\text{Δ}e}{p_{H}-p_{0}}$

$$∴(p_{H}-p_{L})(\text{Δ}x-\text{Δ}m)>\text{min}\{\frac{\beta_{H}p_{L}\text{Δ}e}{p_{L}-p_{0}},\frac{\beta_{H}p_{H}\text{Δ}e}{p_{H}-p_{0}}\}$$

$$>\beta_{H}\text{Δ}e\cdot \text{min}\{\frac{p_{L}}{p_{L}-p_{0}},\frac{p_{H}}{p_{H}-p_{0}}\}$$

$$∴(p_{H}-p_{L})(\text{Δ}x-\text{Δ}m)>\beta_{H}\text{Δ}e\text{ □}$$

Note: Suppose Inequality 7 holds. Adding it to $(p_{H}-p_{L})(\text{Δ}x-\text{Δ}m)>\beta_{H}\text{Δ}e$ gives $(p_{L}-p_{0})(\text{Δ}x-\text{Δ}m)+(p_{H}-p_{L})(\text{Δ}x-\text{Δ}m)>\bar{U}+\beta_{H}e_{L}+\beta_{H}\text{Δ}e\Leftrightarrow (p_{H}-p_{0})(\text{Δ}x-\text{Δ}m)>\bar{U}+\beta_{H}e_{H}$ . So, Inequality 7 ⇒ Inequality 8. Therefore, since at least one of Inequalities 7 and 8 must hold, Inequality 8 must always hold.

As previously shown, $(p_{H}-p_{L})(\text{Δ}x-\text{Δ}m)>\beta_{H}\text{Δ}e$ implies that the firm’s best contract must induce either $\left(e_{H},0\right)$ , $\left(e_{H},e_{L}\right)$ , or $\left(e_{H},e_{H}\right)$ . Among those three options:

$$E[\pi (e_{H},0)]-E[\pi (e_{H},e_{L})]=\gamma_{L}[(p_{L}-p_{0})(\text{Δ}x-\text{Δ}m)-(\bar{U}+\beta_{L}e_{L})]-[(p_{L}-p_{0})(\text{Δ}x-\text{Δ}m)-(\bar{U}+\beta_{H}e_{L})]$$

$$∴\text{ if }(p_{L}-p_{0})(\text{Δ}x-\text{Δ}m)<\bar{U}+\beta_{H}e_{L},\text{ then }E[\pi (e_{H},0)]-\;E[\pi (e_{H},e_{L})]>0,\text{ so }\left(e_{H},0\right)\text{ is preferred over }\left(e_{H},e_{L}\right)\text{for any }\gamma_{L}.$$

$$\text{Otherwise},\;E[\pi (e_{H},0)]>E[\pi (e_{H},e_{L})]\;\Leftrightarrow \gamma_{L}>\frac{\left(p_{L}-p_{0})(\text{Δ}x-\text{Δ}m)-(\bar{U}+\beta_{H}e_{L}\right)}{\left(p_{L}-p_{0})(\text{Δ}x-\text{Δ}m)-(\bar{U}+\beta_{L}e_{L}\right)}$$

$$E[\pi (e_{H},0)]-E[\pi (e_{H},e_{H})]=\gamma_{L}[(p_{H}-p_{0})(\text{Δ}x-\text{Δ}m)\;-(\bar{U}+\beta_{L}e_{H})]-[(p_{H}-p_{0})(\text{Δ}x-\text{Δ}m)-(\bar{U}+\beta_{H}e_{H})]$$

$$∴E[\pi (e_{H},0)]>E[\pi (e_{H},e_{H})]\;\Leftrightarrow \gamma_{L}>\frac{\left(p_{H}-p_{0})(\text{Δ}x-\text{Δ}m)-(\bar{U}+\beta_{H}e_{H}\right)}{\left(p_{H}-p_{0})(\text{Δ}x-\text{Δ}m)-(\bar{U}+\beta_{L}e_{H}\right)}$$

$$E[\pi (e_{H},e_{L})]-E[\pi (e_{H},e_{H})]=\gamma_{L}[(p_{H}-p_{L})(\text{Δ}x-\text{Δ}m)-\beta_{L}\text{Δ}e]\;-[(p_{H}-p_{L})(\text{Δ}x-\text{Δ}m)-\beta_{H}\text{Δ}e]$$

$$∴E[\pi (e_{H},e_{L})]>E[\pi (e_{H},e_{H})]\;\Leftrightarrow \gamma_{L}>\frac{(p_{H}-p_{L})(\text{Δ}x-\text{Δ}m)-\beta_{H}\text{Δ}e}{(p_{H}-p_{L})(\text{Δ}x-\text{Δ}m)-\beta_{L}\text{Δ}e}$$

∴ In each comparison, the firm’s preferred effort if the territory is hard weakly decreases in $\gamma_{L}$ .□

## Appendix C: Proof of Proposition 2

From Lemma 2, we can focus attention on effort combinations rather than the constraints that induce them. Furthermore, it is reasonable to assume that the firm can set requirements such that the manager must exert effort $e_{m}^{r}$ to fulfill them for any $e_{m}^{r}\geq 0$ . Therefore, we can focus on the effort $e_{m}^{r}$ rather than on particular requirements.

From Proposition 1, the firm can narrow consideration to contracts that induce high effort if the territory is easy. So, it considers constraints resulting in $\left(e^{\beta_{L}},e^{\beta_{H}})=(e_{H},0\right)$ , $\left(e_{H},e_{L}\right)$ , or $\left(e_{H},e_{H}\right)$ .

The manager prefers to induce the highest possible effort if the territory is hard. Therefore, the firm’s baseline constraints (i.e., those imposed if the manager makes no request) result in $e^{\beta_{H}}=0$ . The firm then sets requirements for the manager to request relaxed constraints that allow $e^{\beta_{H}}=e_{L}$ and/or $e_{H}$ .

From the proof of Proposition 1 (in Appendix B), the firm’s preference among these options is defined by comparing its expected profits, as follows:

$$E[\pi (e_{H},0)]>E[\pi (e_{H},e_{L})]\Leftrightarrow \;\left\{\begin{array}{cc} (p_{L}-p_{0})(\text{Δ}x-\text{Δ}m)<\bar{U}+\beta_{H}e_{L} & \text{OR}\\ \gamma_{L}>\frac{\left(p_{L}-p_{0})(\text{Δ}x-\text{Δ}m)-(\bar{U}+\beta_{H}e_{L}\right)}{\left(p_{L}-p_{0})(\text{Δ}x-\text{Δ}m)-(\bar{U}+\beta_{L}e_{L}\right)} & \equiv \gamma_{L}^{0} \end{array}\right.$$

$$E[\pi (e_{H},0)]>E[\pi (e_{H},e_{H})]\;\Leftrightarrow \gamma_{L}>\frac{\left(p_{H}-p_{0})(\text{Δ}x-\text{Δ}m)-(\bar{U}+\beta_{H}e_{H}\right)}{\left(p_{H}-p_{0})(\text{Δ}x-\text{Δ}m)-(\bar{U}+\beta_{L}e_{H}\right)}\equiv \gamma_{L}^{1}$$

$$E[\pi (e_{H},e_{L})]>E[\pi (e_{H},e_{H})]\;\Leftrightarrow \gamma_{L}>\frac{(p_{H}-p_{L})(\text{Δ}x-\text{Δ}m)-\beta_{H}\text{Δ}e}{(p_{H}-p_{L})(\text{Δ}x-\text{Δ}m)-\beta_{L}\text{Δ}e}\equiv \gamma_{L}^{2}$$

From these, it is clear that the firm can rule out $\left(e_{H},e_{L}\right)$ for any value of $\gamma_{L}$ when either (1) $(p_{L}-p_{0})(\text{Δ}x-\text{Δ}m)<\bar{U}+\beta_{H}e_{L}$ (in which case $E[\pi (e_{H},0)]>E[\pi (e_{H},e_{L})]$ ) or (2) $\gamma_{L}^{0}<\gamma_{L}^{2}$ (in which case $E[\pi (e_{H},e_{L})]>E[\pi (e_{H},e_{H})]$ implies $E[\pi (e_{H},0)]>E[\pi (e_{H},e_{L})]$ and $E[\pi (e_{H},e_{L})]>E[\pi (e_{H},0)]$ implies $E[\pi (e_{H},e_{H})]>E[\pi (e_{H},e_{L})]$ ). So, this can be considered as a separate case.

$$\text{Case 1:}\;(p_{L}-p_{0})(\text{Δ}x-\text{Δ}m)<\bar{U}+\beta_{H}e_{L}\text{ OR }\gamma_{L}^{0}<\gamma_{L}^{2}$$

In this case, the firm sets baseline constraints that result in $\left(e^{\beta_{L}},e^{\beta_{H}})=(e_{H},0\right)$ and requirements $e_{m}^{r*}$ for the manager to request constraints allowing $\left(e_{H},e_{H}\right)$ . By the definition of $\gamma_{L}^{1}$ , the firm prefers $\left(e_{H},e_{H}\right)$ if and only if $\gamma_{L}<\gamma_{L}^{1}$ .

From Equation 2, the manager will make the request if and only if $\gamma_{L}<\frac{(p_{H}-p_{0})\text{Δ}m-e_{m}^{r*}}{(p_{H}-p_{0})\text{Δ}m}$ .

Therefore, if the firm sets $e_{m}^{r*}=(1-\gamma_{L}^{1})(p_{H}-p_{0})\text{Δ}m$ , then the manager will make the request if and only if $\gamma_{L}<\gamma_{L}^{1}$ , as desired by the firm.

Thus, the mechanism is feasible when $(p_{L}-p_{0})(\text{Δ}x-\text{Δ}m)<\bar{U}+\beta_{H}e_{L}$ or $\gamma_{L}^{0}<\gamma_{L}^{2}$ .

**Case 2:** Otherwise $\left((p_{L}-p_{0})(\text{Δ}x-\text{Δ}m)>\bar{U}+\beta_{H}e_{L}\right.$ AND $\left.\gamma_{L}^{0}>\gamma_{L}^{2}\right)$

Note that the firm can never rule out $\left(e_{H},e_{H}\right)$ as it can $\left(e_{H},e_{L}\right)$ in Case 1. As shown in the proof of Proposition 1 (Appendix B), Inequality 8 always holds. Therefore, the numerator of $\gamma_{L}^{1}$ is positive and is less than its denominator, so $\gamma_{L}^{1}$ is always between 0 and 1.

The firm can also never preemptively rule out $\left(e_{H},0\right)$ . Intuitively, this is because inducing the salesperson to exit when the territory is hard allows the firm to optimize his compensation (i.e., avoid paying rent) when it is easy. Therefore, it is always possible for the likelihood of an easy territory to be sufficiently high (i.e., there exists sufficiently large $\gamma_{L}$ ) that the firm prefers $\left(e_{H},0\right)$ .

Thus, any of the three options can be preferred in this case, depending on $\gamma_{L}$ . By Proposition 1 and the previously discussed profit comparisons, then, the firm prefers $\left\{\begin{array}{ll} \left(e_{H},e_{H}\right) & \text{if }\gamma_{L}<\gamma_{L}^{2}\\ \left(e_{H},e_{L}\right) & \text{if }\gamma_{L}^{2}<\gamma_{L}<\gamma_{L}^{0}\\ \left(e_{H},0\right) & \text{if }\gamma_{L}^{0}<\gamma_{L} \end{array}\right.$

The firm sets default constraints that result in $\left(e^{\beta_{L}},e^{\beta_{H}})=(e_{H},0\right)$ and it sets sequential requirements $e_{m}^{rL}$ and $e_{m}^{rH}$ for the manager to request constraints allowing $\left(e_{H},e_{L}\right)$ and $\left(e_{H},e_{H}\right)$ , respectively. In other words, the manager must complete the first request before she can make the second one.

From Equation 2, the manager makes the first request if and only if $\gamma_{L}<\frac{(p_{L}-p_{0})\text{Δ}m-e_{m}^{rL}}{(p_{L}-p_{0})\text{Δ}m}$ . If the firm sets $e_{m}^{rL}=(1-\gamma_{L}^{0})(p_{L}-p_{0})\text{Δ}m$ , then the manager makes the request if and only if $\gamma_{L}<\gamma_{L}^{0}$ , as desired. The manager then makes the second request if and only if $\gamma_{L}<\frac{(p_{H}-p_{L})\text{Δ}m-e_{m}^{rH}}{(p_{H}-p_{L})\text{Δ}m}$ . Thus, if $e_{m}^{rH}=(1-\gamma_{L}^{2})(p_{H}-p_{L})\text{Δ}m$ , then the manager makes the request if and only if $\gamma_{L}<\gamma_{L}^{2}$ .

There are three remaining questions to consider: (1) Does the sequential nature of these requests ever prevent the first request from being made when the second is desired (by the firm)? (2) Does the manager ever make a suboptimal first request to gain the opportunity to make the second? (3) Why must the requests be sequential? These are addressed in Web Appendix D.

## Appendix D: Sketch Proof of Proposition 3

First, consider the firm’s expected profits using a menu of contracts. (For the optimal design of the menu, see the proof of Lemma 4 in Web Appendix E.)

When deciding whether to accept her contract, the manager anticipates the menu that the firm will offer the salesperson and the resulting $\left(e^{\beta_{L}},e^{\beta_{H}}\right)$ . Because the firm does not observe the manager’s belief, it must ensure that she will accept for any $\gamma_{L}\in (0,1)$ . Her expected utility weakly increases with $\gamma_{L}$ , so it is necessary and sufficient to ensure that she accepts for $\gamma_{L}\to 0$ . Given that constraint, the firm maximizes its expected profit, so it prefers to minimize $m_{L}$ , resulting in $m_{L}={\bar{U}}_{m}-p(e^{\beta_{H}})\text{Δ}m$ .

Therefore, the firm’s expected profit given an efficient menu of contracts to induce $\left(e^{\beta_{L}},e^{\beta_{H}}\right)$ is

9
$$\begin{array}{l} E[\pi (e^{\beta_{L}},e^{\beta_{H}})]=x_{L}-[{\bar{U}}_{m}-p(e^{\beta_{H}})\text{Δ}m]\\ +\gamma_{L}^{F}\left[p(e^{\beta_{L}})(\text{Δ}x-\text{Δ}m)-(\bar{U}+\beta_{L}e^{\beta_{L}}+\text{Δ}\beta e^{\beta_{H}})\right]\\ +(1-\gamma_{L}^{F})\left[p(e^{\beta_{H}})(\text{Δ}x-\text{Δ}m)-1_{e^{\beta_{H}}>0}(\bar{U}+\beta_{H}e^{\beta_{H}})\right], \end{array}$$

where the indicator function $1_{e^{\beta_{H}}>0}$ equals 0 if the salesperson exits when the territory is hard and 1 otherwise.

By Lemma 4, the relevant cases under a menu are the same as under the request mechanism, as given in the proof of Proposition 2 (Appendix C).

### Comparison of Expected Profits

**Case 1:** $(p_{L}-p_{0})(\text{Δ}x-\text{Δ}m)<\bar{U}+\beta_{H}e_{L}$ OR $\gamma_{L}^{0}<\gamma_{L}^{2}$

The firm prefers to induce $\left(e^{\beta_{L}},e^{\beta_{H}})=(e_{H},e_{H}\right)$ if and only if $\gamma_{L}<\gamma_{L}^{1}=\frac{\left(p_{H}-p_{0})(\text{Δ}x-\text{Δ}m)-(\bar{U}+\beta_{H}e_{H}\right)}{\left(p_{H}-p_{0})(\text{Δ}x-\text{Δ}m)-(\bar{U}+\beta_{L}e_{H}\right)}$ and $\left(e_{H},0\right)$ otherwise.

Under a menu of contracts, the firm chooses on the basis of its belief, $\gamma_{L}^{F}=.5$ . From Equation 9:

$$E[\pi |\text{menu},\gamma_{L}^{1}<.5]=x_{L}+p_{0}\text{Δ}x-{\bar{U}}_{m}\;+\frac{1}{2}\left[\left(p_{H}-p_{0})(\text{Δ}x-\text{Δ}m)-(\bar{U}+\beta_{L}e_{H}\right)\right]$$

$$E[\pi |\text{menu},\gamma_{L}^{1}>.5]=x_{L}+p_{H}\text{Δ}x-({\bar{U}}_{m}+\bar{U}+\beta_{H}e_{H}).$$

Under the request mechanism, the manager chooses on the basis of her own belief, which is unknown to the firm. Therefore, the expected profit is:

$$E[\pi |\text{req}]=\int_{0}^{\gamma_{L}^{1}}E[\pi (e_{H},e_{H})]f(\gamma_{L})\text{d}\gamma_{L}+\int_{\gamma_{L}^{1}}^{1}E[\pi (e_{H},0)]f(\gamma_{L})\text{d}\gamma_{L}$$

The manager’s signal $Q∼U(.5,1)\Rightarrow \gamma_{L}∼U(0,1)$ , so $f(\gamma_{L})=1$ for all values of $\gamma_{L}\in (0,1)$ . Therefore, substituting in Equation 3 and $m_{L}$ from Table 3 gives

$$E[\pi |\text{req}]=x_{L}+p_{0}\text{Δ}x-{\bar{U}}_{m}+\gamma_{L}^{1}\left[\left(p_{H}-p_{0})\text{Δ}x-(\bar{U}+\beta_{H}e_{H}\right)\right]$$

$$+(1-\gamma_{L}^{1})\frac{1+\gamma_{L}^{1}}{2}\left[\left(p_{H}-p_{0})(\text{Δ}x-\text{Δ}m)-(\bar{U}+\beta_{L}e_{H}\right)\right].$$

Now the two approaches can be compared. First, when $\gamma_{L}^{1}<0.5$ ,

$$E[\pi |\text{req}]-E[\pi |\text{menu},\gamma_{L}^{1}<.5]=\gamma_{L}^{1}\left(\frac{\gamma_{L}^{1}}{2}[\left(p_{H}-p_{0})(\text{Δ}x-\text{Δ}m)\;-(\bar{U}+\beta_{L}e_{H}\right),] +(p_{H}-p_{0})\text{Δ}m\right)>0.$$

Therefore, the firm prefers the request mechanism in Case 1 when its best menu of contracts induces the salesperson to exit if his territory is hard.

Next, consider $\gamma_{L}^{1}>.5:$ $E[\pi |\text{req}]-E[\pi |\text{menu},\gamma_{L}^{1}>.5]=(1-\gamma_{L}^{1})\left(\frac{\text{Δ}\beta e_{H}}{2}-(p_{H}-p_{0})\text{Δ}m\right)\text{is positive if and only if}\text{Δ}m<\frac{\text{Δ}\beta e_{H}}{2(p_{H}-p_{0})}$ . Therefore, when the firm’s best menu of contracts does not induce exit in Case 1, the request mechanism is preferred as long as Δm is not too large.

**Case 2:** Otherwise $\left((p_{L}-p_{0})(\text{Δ}x-\text{Δ}m)>\bar{U}+\beta_{H}e_{L}\right.$ AND $\left.\gamma_{L}^{0}>\gamma_{L}^{2}\right)$

The firm considers all three of $\left(e^{\beta_{L}},e^{\beta_{H}})=(e_{H},0\right)$ , (e_H_, e_L_) and $\left(e_{H},e_{H}\right)$ , choosing as follows: $\left\{\begin{array}{ll} \left(e_{H},0\right) & \text{if }\gamma_{L}^{0}<\gamma_{L}\\ \left(e_{H},e_{L}\right) & \text{if }\gamma_{L}\in (\gamma_{L}^{2},\gamma_{L}^{0})\\ \left(e_{H},e_{H}\right) & \text{if }\gamma_{L}<\gamma_{L}^{2} \end{array}\right.$ , where $\gamma_{L}^{0}=\frac{\left(p_{L}-p_{0})(\text{Δ}x-\text{Δ}m)-(\bar{U}+\beta_{H}e_{L}\right)}{\left(p_{L}-p_{0})(\text{Δ}x-\text{Δ}m)-(\bar{U}+\beta_{L}e_{L}\right)}$ , $\gamma_{L}^{1}=\frac{\left(p_{H}-p_{0})(\text{Δ}x-\text{Δ}m)-(\bar{U}+\beta_{H}e_{H}\right)}{\left(p_{H}-p_{0})(\text{Δ}x-\text{Δ}m)-(\bar{U}+\beta_{L}e_{H}\right)}$ , and $\gamma_{L}^{2}=\frac{(p_{H}-p_{L})(\text{Δ}x-\text{Δ}m)-\beta_{H}\text{Δ}e}{(p_{H}-p_{L})(\text{Δ}x-\text{Δ}m)-\beta_{L}\text{Δ}e}$ .

Again, under the menu of contracts, the firm chooses on the basis of its own belief, $\gamma_{L}^{F}=.5$ . From Equation 9, its expected profits are:

$$E[\pi |\text{menu},\gamma_{L}^{0}<.5]=x_{L}+p_{0}\text{Δ}x-{\bar{U}}_{m}\;+\frac{1}{2}\left[\left(p_{H}-p_{0})(\text{Δ}x-\text{Δ}m)-(\bar{U}+\beta_{L}e_{H}\right)\right]$$

$$E[\pi |\text{menu},.5\in (\gamma_{L}^{2},\gamma_{L}^{0})]=x_{L}+p_{L}\text{Δ}x-{\bar{U}}_{m}-(\bar{U}+\beta_{H}e_{L})\;+\frac{1}{2}\left[(p_{H}-p_{L})(\text{Δ}x-\text{Δ}m)-\beta_{L}\text{Δ}e\right]$$

$$E[\pi |\text{menu},\gamma_{L}^{2}>.5]=x_{L}+p_{H}\text{Δ}x-{\bar{U}}_{m}-(\bar{U}+\beta_{H}e_{H}).$$

Under the request mechanism, the firm’s expected profit is

$$E[\pi |\text{req}]=\int_{0}^{\gamma_{L}^{2}}E[\pi (e_{H},e_{H})]f(\gamma_{L})\text{d}\gamma_{L}\;+\int_{\gamma_{L}^{2}}^{\gamma_{L}^{0}}E[\pi (e_{H},e_{L})]f(\gamma_{L})\text{d}\gamma_{L}+\int_{\gamma_{L}^{0}}^{1}E[\pi (e_{H},0)]f(\gamma_{L})\text{d}\gamma_{L}.$$

Substituting in Equation 3 and $m_{L}$ from Table 3 gives

$$E[\pi |\text{req}]=x_{L}+p_{0}\text{Δ}x-{\bar{U}}_{m}+\gamma_{L}^{0}\left[\left(p_{L}-p_{0})\text{Δ}x-(\bar{U}+\beta_{H}e_{L}\right)\right]\;+\gamma_{L}^{2}\left[(p_{H}-p_{L})\text{Δ}x-\beta_{H}\text{Δ}e\right]+(\gamma_{L}^{0}-\gamma_{L}^{2})\frac{\gamma_{L}^{0}+\gamma_{L}^{2}}{2}$$

$$\left[(p_{H}-p_{L})(\text{Δ}x-\text{Δ}m)-\beta_{L}\text{Δ}e\right]+(1-\gamma_{L}^{0})\frac{1+\gamma_{L}^{0}}{2}[\left(p_{H}-p_{0})\;(\text{Δ}x-\text{Δ}m)-(\bar{U}+\beta_{L}e_{H}\right)]$$

Now the two approaches can be compared. First, when $\gamma_{L}^{1}<.5$ ,

$$E[\pi |\text{req}]-E[\pi |\text{menu},\gamma_{L}^{0}<.5]=\left[\gamma_{L}^{0}(p_{L}-p_{0})+\gamma_{L}^{2}(p_{H}-p_{L})\right]$$

$$\text{Δ}m+\frac{{\left(\gamma_{L}^{0}\right)}^{2}}{2}\left[\left(p_{L}-p_{0})(\text{Δ}x-\text{Δ}m)-(\bar{U}+\beta_{L}e_{L}\right)\right]\;+\frac{{\left(\gamma_{L}^{2}\right)}^{2}}{2}\left[(p_{H}-p_{L})(\text{Δ}x-\text{Δ}m)-\beta_{L}\text{Δ}e\right].$$

From the definition of Case 2, $(p_{L}-p_{0})(\text{Δ}x-\text{Δ}m)>\bar{U}+\beta_{H}e_{L}>\bar{U}+\beta_{L}e_{L}$ and from the proof of Proposition 1 (Appendix B), $(p_{H}-p_{L})(\text{Δ}x-\text{Δ}m)>\beta_{H}\text{Δ}e>\beta_{L}\text{Δ}e$ . Therefore, $E[\pi |\text{req}]-E[\pi |\text{menu},\gamma_{L}^{0}<.5]>0$ , so the firm prefers the request mechanism in Case 2 when its best menu of contracts induces the salesperson to exit if his territory is hard.

Next, consider $.5\in (\gamma_{L}^{2},\gamma_{L}^{0})$ :

$$E[\pi |\text{req}]-E[\pi |\text{menu},.5\in (\gamma_{L}^{2},\gamma_{L}^{0})]=\frac{1}{2}\left[(p_{H}-p_{L})\text{Δ}x-\beta_{H}\text{Δ}e\right]+\frac{1-\gamma_{L}^{0}}{2}\text{Δ}\beta e_{L}-\frac{\gamma_{L}^{2}}{2}\text{Δ}\beta \text{Δ}e$$

$$-\left[\left(\frac{1}{2}-\gamma_{L}^{2}\right)(p_{H}-p_{L})+(1-\gamma_{L}^{0})(p_{L}-p_{0})\right]\text{Δ}m$$

∴ the request mechanism is preferred if and only if $\text{Δ}m<\frac{(p_{H}-p_{L})\text{Δ}x-\beta_{H}\text{Δ}e+(1-\gamma_{L}^{0})\text{Δ}\beta e_{L}-\gamma_{L}^{2}\text{Δ}\beta \text{Δ}e}{2\left[\left(\frac{1}{2}-\gamma_{L}^{2}\right)(p_{H}-p_{L})+(1-\gamma_{L}^{0})(p_{L}-p_{0})\right]}$ Therefore, when the firm’s best menu of contracts induces $\left(e_{H},e_{L}\right)$ , the request mechanism is preferred as long as Δm is not too large.

Finally, consider $\gamma_{L}^{2}>.5$ :

$$E[\pi |\text{req}]-E[\pi |\text{menu},\gamma_{L}^{2}>.5]=\frac{1}{2}\left[(1-\gamma_{L}^{0})\text{Δ}\beta e_{L}+(1-\gamma_{L}^{2})\text{Δ}\beta \text{Δ}e\right]-[(1-\gamma_{L}^{0})(p_{L}-p_{0})$$

$$+(1-\gamma_{L}^{2})(p_{H}-p_{L})]\text{Δ}m$$

∴ the request mechanism is preferred if and only if $\text{Δ}m<\frac{(1-\gamma_{L}^{0})\text{Δ}\beta e_{L}+(1-\gamma_{L}^{2})\text{Δ}\beta \text{Δ}e}{2\left[\left(1-\gamma_{L}^{0})(p_{L}-p_{0})+(1-\gamma_{L}^{2})(p_{H}-p_{L}\right)\right]}$

Thus, when the firm’s best menu of contracts induces $\left(e_{H},e_{H}\right)$ , the request mechanism is preferred as long as Δm is not too large. Therefore, the request mechanism is preferred when the firm’s best menu of contracts induces the salesperson to exit or when Δm is not too large.

## Notes

1 For convenience and clarity, I use feminine pronouns (she/her) to refer to managers and masculine pronouns (he/his) to refer to salespeople throughout this article.

2 The simplest theoretical solution to this conflict is to offer the manager a residual claimancy contract. However, I take the wide use of sales-based incentives as evidence of a managerially relevant institutional constraint and reflect that in the model assumptions.

3 The model is based on a B2B, “outside sales” context but can be applied whenever (1) an employee whose effort is unobserved receives incentives on the basis of an outcome other than firm profit, (2) his manager has relevant information that she can choose to share with the firm, and (3) the manager’s incentives are aligned with those of the employee.

4 Positive sales in the absence of a salesperson represent customers arriving due to word of mouth, advertising, or other demand generation activities and/or existing customers making repeat/additional purchases.

5 Territory difficulty could also be modeled through the effectiveness of sales effort, by a multiplier on the probability of high sales, or using both of these. They would be effectively equivalent to the current model.

6 Omitting the manager’s other tasks from the model effectively implies that those tasks are either completed before compensation design or do not affect it.

7 This is because, in equilibrium, the manager correctly anticipates in Step 2 the constraints and requirements that the firm will announce in Step 3.

8 Even when a fully informed firm would prefer $e_{H}$ in both territory types, an uninformed firm might prefer a different $\left(e^{\beta_{L}},e^{\beta_{H}}\right)$ , as shown subsequently.

9 Analysis of this comparison is substantially more complex because the threshold values that determine the firm’s optimal choices now differ between the menu and request mechanisms.
