# Supplementary material for: Involving Sales Managers in Sales Force Compensation Design
Source: J Mark Res. 2020 Dec 14;58(1):182–201. doi: 10.1177/0022243720969174 (PMC13038172; doi:10.1177/0022243720969174)
Supplement: Supplemental Material, Involving_Sls_Mgrs_in_SF_Incentive_Design_v9.0_JMR_revision_4_(Web_Appendix) - Involving Sales Managers in Sales Force Compensation Design [file Involving_Sls_Mgrs_in_SF_Incentive_Design_v9.0_JMR_revision_4_(Web_Appendix).pdf]

WEB APPENDIX A: PROOF OF LEMMA 1

There are six possible combinations of effort $(e^{\beta_L}, e^{\beta_H})$ with $e^{\beta_L} \geq e^{\beta_H}$: $(0,0)$, $(e_L, 0)$, (e_L, e_L), $(e_H, 0)$, (e_H, e_L), and (e_H, e_H).

Trivially, the combination $(e^{\beta_L}, e^{\beta_H}) = (0,0)$ can be induced with an offer of $s_L = s_H = 0$, resulting in the salesperson exiting regardless of his territory type.

Effort e_L can be induced by offering no bonus and a salary at least equal to the salesperson's outside option plus the cost of low effort in that territory.

Thus, $(e^{\beta_L}, e^{\beta_H}) = (e_L, 0)$ can be induced with $s_L = s_H = \bar{U} + \beta_L e_L$, which satisfies (IR_L) if $\beta = \beta_L$ but not if $\beta = \beta_H$.

Similarly, (e_L, e_L) can be induced with $s_L = s_H = \bar{U} + \beta_H e_L$, which satisfies (IR_L) for either value of β.

Effort e_H requires both (IR_H) and $\beta \Delta e \leq (p_H - p_L)\Delta s$.

Suppose the latter binds for $\beta = \beta_L$ (i.e., $\Delta s = \frac{\beta_L \Delta e}{p_H - p_L}$). Then it does not hold for $\beta = \beta_H$, so the salesperson either exits or exerts e_L if the territory is hard. More specifically, he exits if s_L is low, but stays and exerts e_L if s_L is sufficiently high (holding Δs constant).

For example, if (IR_H) also binds when $\beta = \beta_L$, then $s_L = \bar{U} + \beta_L e_H - \frac{p_H \beta_L \Delta e}{p_H - p_L}$. Then

$$
\begin{aligned}
s_L + p_L \Delta s - \beta_H e_L &= \bar{U} + \beta_L e_H - \frac{p_H \beta_L \Delta e}{p_H - p_L} + \frac{p_L \beta_L \Delta e}{p_H - p_L} - \beta_H e_L \\
&= \bar{U} + \beta_L e_H - \beta_L \Delta e - \beta_H e_L \\
&= \bar{U} - \Delta\beta e_L \\
&< \bar{U}
\end{aligned}
$$

So (IR_L) fails and the salesperson exits if $\beta = \beta_H$, so this contract induces $(e^{\beta_L}, e^{\beta_H}) = (e_H, 0)$.

On the other hand, s_L can be made arbitrarily high to satisfy (IR_L) for both types and induce $(e^{\beta_L}, e^{\beta_H}) = (e_H, e_L)$.

Trivially, (e_H, e_H) can be induced by offering an arbitrarily high s_H with any non-negative s_L.

Thus, in the absence of constraints, there exists a contract to induce any combination of efforts $(e^{\beta_L}, e^{\beta_H})$ with $e^{\beta_L} \geq e^{\beta_H}$. □

WEB APPENDIX B: PROOF OF LEMMA 2

By assumption, when the manager is indifferent between choices of (s_L, s_H) (i.e., when multiple contracts induce the same $(e^{\beta_L}, e^{\beta_H})$), she chooses optimally for the firm.

Thus, if the firm delegates to the manager with no constraints, she will choose an efficient contract that induces $\boldsymbol{(e^{\beta_L}, e^{\beta_H}) = (e_H, e_H)}$.

In general, the firm's best contract to induce non-zero efforts $(e^{\beta_L}, e^{\beta_H})$ solves the following:

$$\max_{(s_L, s_H)} E[\pi] = x_L - m_L - s_L + \left(\gamma_L^F p[e^{\beta_L}] + [1 - \gamma_L^F] p[e^{\beta_H}]\right) (\Delta x - \Delta m - \Delta s)$$

$$\text{s.t. } s_L + p(e^{\beta_L})\Delta s - \beta_L e^{\beta_L} \geq \bar{U} \quad (\text{IR}^{\beta_L})$$

$$s_L + p(e^{\beta_H})\Delta s - \beta_H e^{\beta_H} \geq \bar{U} \quad (\text{IR}^{\beta_H})$$

$$s_L + p(e^{\beta_L})\Delta s - \beta_L e^{\beta_L} \geq s_L + p(e^*)\Delta s - \beta_L e^* \quad \forall e^* \in \{e_L, e_H\} \quad (\text{IC}^{\beta_L})$$

$$s_L + p(e^{\beta_H})\Delta s - \beta_H e^{\beta_H} \geq s_L + p(e^*)\Delta s - \beta_H e^* \quad \forall e^* \in \{e_L, e_H\} \quad (\text{IC}^{\beta_H})$$

$$s_L \geq 0 \quad (\text{LL})$$

First, consider the identical problem, but with the (LL) constraint removed. I will show that the solutions identified for this reduced problem remain feasible when (LL) is imposed. Since the complete problem contains all of the same constraints as the reduced one, plus (LL), this implies that the solutions to the latter are also solutions to the former.

At least one of the (IR) constraints must bind. (Otherwise, the firm could decrease s_L while holding Δs constant, increasing the objective function without violating any constraints.)

Now, by (IC$^{\beta_L}$):

$$s_L + p(e^{\beta_L})\Delta s - \beta_L e^{\beta_L} \geq s_L + p(e^{\beta_H})\Delta s - \beta_L e^{\beta_H}$$
$$\therefore \quad \geq \bar{U} + \Delta\beta e^{\beta_H} \qquad \text{by (IR}^{\beta_H}\text{)}$$

$\therefore$ (IR$^{\beta_L}$) holds with slack, so (IR$^{\beta_H}$) must bind in any efficient contract.

$\therefore s_L = \bar{U} + \beta_H e^{\beta_H} - p(e^{\beta_H})\Delta s$

$\therefore s_H = s_L + \Delta s = \bar{U} + \beta_H e^{\beta_H} + [1 - p(e^{\beta_H})]\Delta s$

Suppose $(e^{\beta_L}, e^{\beta_H}) = (e_H, e_H)$

By (IC$^{\beta_H}$), $\Delta s \geq \frac{\beta_H \Delta e}{p_H - p_L}$

$\therefore s_H \geq \bar{U} + \beta_H e_H + \frac{(1-p_H)\beta_H \Delta e}{p_H - p_L}$

Thus, while no constraint is necessary, the lowest total payout cap that *allows* (e_H, e_H) is

$$\widehat{s_H}^{(e_H,e_H)} = \bar{U} + \beta_H e_H + \frac{(1 - p_H)\beta_H \Delta e}{p_H - p_L}$$

Now, suppose $(e^{\beta_L}, e^{\beta_H}) = (e_H, e_L)$:

(IR$^{\beta_H}$) binds $\implies s_L = \bar{U} + \beta_H e_L - p_L \Delta s$

From (IC$^{\beta_L}$), $\Delta s \geq \frac{\beta_L \Delta e}{p_H - p_L}$

The firm's objective function can be simplified to:

$$\max_{(s_L, s_H)} - \left(s_L + \left[\gamma_L^F p_H + (1 - \gamma_L^F) p_L\right] \Delta s\right)$$

Substituting for s_L gives:

$$\max_{(s_L, s_H)} - \left(\bar{U} + \beta_H e_L + \gamma_L^F [p_H - p_L]\Delta s\right)$$
$$\therefore \max_{(s_L, s_H)} - \Delta s$$

Therefore, under the efficient contract for (e_H, e_L), $\Delta s = \frac{\beta_L \Delta e}{p_H - p_L}$

$\therefore s_H = \bar{U} + \beta_H e_L + \frac{(1-p_L)\beta_L \Delta e}{p_H - p_L}$

Suppose, then, that the firm sets $\widehat{s_H}^{(e_H,e_L)} = \bar{U} + \beta_H e_L + \frac{(1-p_L)\beta_L \Delta e}{p_H - p_L}$

$$
\begin{aligned}
\widehat{s_H}^{(e_H,e_H)} - \widehat{s_H}^{(e_H,e_L)} &= \bar{U} + \beta_H e_H + \frac{(1-p_H)\beta_H \Delta e}{p_H - p_L} - \left(\bar{U} + \beta_H e_L + \frac{(1-p_L)\beta_L \Delta e}{p_H - p_L}\right) \\
&= \beta_H \Delta e + \frac{\Delta e}{p_H - p_L}\left[(1-p_H)\beta_H - (1-p_L)\beta_L\right] \\
&= \frac{\Delta e}{p_H - p_L}\left[\beta_H(p_H - p_L) + \Delta\beta - \beta_H p_H + \beta_L p_L\right] \\
&= \frac{(1-p_L)\Delta\beta\Delta e}{p_H - p_L} \\
&> 0 \\
\therefore \widehat{s_H}^{(e_H,e_L)} &< \widehat{s_H}^{(e_H,e_H)}
\end{aligned}
$$

From above, $\widehat{s_H}^{(e_H,e_H)}$ is the lowest total payout cap that allows (e_H, e_H). Therefore, $\widehat{s_H}^{(e_H,e_L)}$ does *not* allow (e_H, e_H), so the manager will choose to implement $\boldsymbol{(e^{\beta_L}, e^{\beta_H}) = (e_H, e_L)}$ efficiently. Trivially, $\boldsymbol{(e^{\beta_L}, e^{\beta_H}) = (0, 0)}$ can be implemented efficiently by setting $\widehat{s_H}^{(0,0)} = 0$.

Next, suppose the firm constrains the total payout with $\widehat{s_H}^{(e_L,0)} = \bar{U} + \beta_L e_L$.

Under this constraint, (IR^{β_L}) can be satisfied (and binds) only when $s_L = s_H = \bar{U} + \beta_L e_L$. Under that contract, $e^{\beta_L} = e_L$ and (IR^{β_H}) is not satisfed for any e.

Thus, $\widehat{s_H}^{(e_L,0)}$ is sufficient to implement $\boldsymbol{(e^{\beta_L}, e^{\beta_H}) = (e_L, 0)}$.

That leaves $\boldsymbol{(e_H, 0)}$ and $\boldsymbol{(e_L, e_L)}$:

Under an efficient contract for $(e_H, 0)$, the salesperson is expected to exit if the territory is hard, so the firm's efficient contract matches that of a fully informed firm in an easy territory. Therefore, from the standard principal-agent model, (IR^{β_L}) must bind, so $s_L = \bar{U} + \beta_L e_H - p_H \Delta s$

To induce e_H, by (IC^{β_L}), $\Delta s \geq \frac{\beta_L \Delta e}{p_H - p_L}$.

$\therefore s_H = \bar{U} + \beta_L e_H + (1-p_H)\Delta s \geq \bar{U} + \beta_L e_H + \frac{(1-p_H)\beta_L \Delta e}{p_H - p_L}$

Thus, the minimum total payout cap that could allow $(e_H, 0)$ to be implemented efficiently is $\widehat{s_H}^{(e_H,0)} = \bar{U} + \beta_L e_H + \frac{(1-p_H)\beta_L \Delta e}{p_H - p_L}$

Given that cap, if the manager chooses to induce $e^{\beta_L} = e_H$, by offering $(s_L, s_H) = (\bar{U} + \beta_L e_H - p_H \Delta s, \widehat{s_H}^{(e_H,0)})$, it is straightforward to show that (IR^{β_H}) cannot be satisfied for e_L or e_H, so the salesperson does, indeed, exit if the territory is hard. In other words, $\widehat{s_H}^{(e_H,0)}$ allows $(e_H, 0)$, but not

(e_H, e) for $e > 0$.

From above, under the efficient contract for (e_L, e_L), (IR^{β_H}) must bind, so $s_L = \bar{U} + \beta_H e_L - p_L \Delta s$.

Since there is no minimum constraint on Δs to induce e_L, the minimum total payout cap that could allow (e_L, e_L) is simply $\widehat{s_H}^{(e_L,e_L)} = \bar{U} + \beta_H e_L$.

If the total payout cap allows the manager to choose between $(e_H, 0)$ and (e_L, e_L), her choice depends on her belief γ_L, which the firm does not observe. Therefore, if the firm sets $\widehat{s_H} = \max\{\widehat{s_H}^{(e_H,0)}, \widehat{s_H}^{(e_L,e_L)}\}$, then it is unclear what contract (i.e., which combination of efforts) the manager will choose. Thus, a total payout cap alone is not sufficient to implement the firm's desired outcome.

Case 1: Suppose $\widehat{s_H}^{(e_L,e_L)} < \widehat{s_H}^{(e_H,0)}$:

It is straightforward to show that $\widehat{s_H}^{(e_H,0)} < \widehat{s_H}^{(e_H,e_L)}$.

From above, then, $\widehat{s_H}^{(e_L,e_L)}$ is sufficient to implement (e_L, e_L) efficiently (because it prevents the manager from inducing any preferred combination of efforts).

To implement $(e_H, 0)$, suppose the firm imposes salary cap $\widehat{s_L}^{(e_H,0)} = \bar{U} + \beta_L e_H - \frac{p_H \beta_L \Delta e}{p_H - p_L}$, in addition to $\widehat{s_H}^{(e_H,0)} = \bar{U} + \beta_L e_H + \frac{(1-p_H)\beta_L \Delta e}{p_H - p_L}$.

Under $(s_L, s_H) = (\widehat{s_L}^{(e_H,0)}, \widehat{s_H}^{(e_H,0)})$, (IR^{β_L}) and (IC^{β_L}) bind when $e = e_H$, while (IR^{β_H}) cannot be satisfied for either $e > 0$.

Therefore, $(\widehat{s_L}^{(e_H,0)}, \widehat{s_H}^{(e_H,0)})$ is sufficient to implement $(e_H, 0)$).

Case 2: Suppose $\widehat{s_H}^{(e_H,0)} < \widehat{s_H}^{(e_L,e_L)}$:

It is straightforward to show that $\widehat{s_H}^{(e_L,e_L)} < \widehat{s_H}^{(e_H,e_L)}$.

From above, then, $\widehat{s_H}^{(e_H,0)}$ is sufficient to implement $(e_H, 0)$.

To implement (e_L, e_L), suppose the firm imposes $\widehat{\Delta s}^{(e_L,e_L)} = 0$ in addition to $\widehat{s_H}^{(e_L,e_L)} = \bar{U} + \beta_H e_L$.

This clearly prohibits any contract that induces effort e_H, making $(\widehat{\Delta s}^{(e_L,e_L)}, \widehat{s_H}^{(e_L,e_L)})$ sufficient to implement (e_L, e_L).

Claim: The solutions above remain feasible when the (LL) constraint is applied, implying that they are also solutions to this more-constrained problem.

Proof: Under $\widehat{s_H}^{(e_H,e_H)} = \bar{U} + \beta_H e_H + \frac{(1-p_H)\beta_H \Delta e}{p_H - p_L}$, the manager can induce (e_H, e_H) by offering

$(s_L, s_H) = (\bar{U} + \beta_H e_H - \frac{p_H \beta_H \Delta e}{p_H - p_L}, \widehat{s_H}^{(e_H, e_H)})$. By assumption, $\bar{U} \geq \beta_H(\frac{p_L e_H - p_H e_L}{p_H - p_L}) \geq 0$, so $\bar{U} + \beta_H e_H - \frac{p_H \beta_H \Delta e}{p_H - p_L} \geq 0$. Therefore, (LL) is not violated.

Under $\widehat{s_H}^{(e_H, e_L)} = \bar{U} + \beta_H e_L + \frac{(1-p_L)\beta_L \Delta e}{p_H - p_L}$, the manager can induce (e_H, e_L) by offering $(s_L, s_H) = (\bar{U} + \beta_H e_L - \frac{p_L \beta_L \Delta e}{p_H - p_L}, \widehat{s_H}^{(e_H, e_L)})$. By the same assumption, $\bar{U} + \beta_H e_L - \frac{p_L \beta_L \Delta e}{p_H - p_L} \geq \frac{p_L \Delta\beta \Delta e}{p_H - p_L} > 0$, so (LL) is not violated.

Under $(\widehat{s_L}^{(e_H, 0)}, \widehat{s_H}^{(e_H, 0)}) = (\bar{U} + \beta_L e_H - \frac{p_H \beta_L \Delta e}{p_H - p_L}, \bar{U} + \beta_L e_H + \frac{(1-p_H)\beta_L \Delta e}{p_H - p_L})$, the manager can induce $(e_H, 0)$ by offering $(s_L, s_H) = (\widehat{s_L}^{(e_H, 0)}, \widehat{s_H}^{(e_H, 0)})$. Here, $\bar{U} + \beta_L e_H - \frac{p_H \beta_L \Delta e}{p_H - p_L} \geq \Delta\beta(\frac{p_L e_H - p_H e_L}{p_H - p_L}) \geq 0$, so (LL) is not violated.

Under $(\widehat{\Delta s}^{(e_L, e_L)}, \widehat{s_H}^{(e_L, e_L)}) = (0, \bar{U} + \beta_H e_L)$, the manager can induce (e_L, e_L) by offering $(s_L, s_H) = (\widehat{s_H}^{(e_L, e_L)}, \widehat{s_H}^{(e_L, e_L)})$. Clearly, $\bar{U} + \beta_H e_L > 0$, so (LL) is not violated.

Similarly, under $\widehat{s_H}^{(e_L, 0)} = \bar{U} + \beta_L e_L$, the manager can induce $(e_L, 0)$ by offering $(s_L, s_H) = (\widehat{s_H}^{(e_L, 0)}, \widehat{s_H}^{(e_L, 0)})$. Again, it is clear that $\bar{U} + \beta_L e_L > 0$, so (LL) is not violated. □

Therefore, a hard cap on the salesperson's total payout combined with a hard cap on either his salary or his bonus is sufficient to implement an efficient contract for any combination of efforts $(e^{\beta_L}, e^{\beta_H})$ with $e^{\beta_L} \geq e^{\beta_H}$. □

WEB APPENDIX C: PROOF OF LEMMA 3

Suppose $e^{\beta_H} = 0$, so the salesperson will exit if the territory is hard. In that case, the firm can simply act as though it knows that the territory is easy, so the efficient contract matches the full-information (i.e., standard principal-agent model) solution for an easy territory. It is straightforward to show that the salesperson will, indeed, exit (and receive no surplus) under that contract if the territory is hard.

In the standard principal-agent model, (IR^{β_L}) binds under the full-information solution, so the salesperson's surplus is 0 $(= \Delta\beta e^{\beta_H})$ if the territory is easy.

Therefore, the Lemma holds when $e^{\beta_H} = 0$.

Next, **suppose $e^{\beta_L} = e^{\beta_H} > 0$**:

From the proof of Lemma 2 (Web Appendix B), (IR^{β_H}) binds in any efficient contract inducing non-zero $(e^{\beta_L}, e^{\beta_H})$, so the salesperson receives no surplus when the territory is hard.

$$\therefore s_L + p(e^{\beta_H})\Delta s - \beta_H e^{\beta_H} = \bar{U}$$

$$\therefore s_L + p(e^{\beta_L})\Delta s - \beta_L e^{\beta_L} = \bar{U} + \Delta\beta e^{\beta_H} \qquad \text{by } e^{\beta_L} = e^{\beta_H}$$

$\therefore$ the salesperson receives a surplus of $\Delta\beta e^{\beta_H}$ when it is easy.

Therefore, the Lemma holds when $e^{\beta_L} = e^{\beta_H} > 0$

Lastly, **suppose $(e^{\beta_L}, e^{\beta_H}) = (e_H, e_L)$**:

From Web Appendix B, the efficient contract to induce (e_H, e_L) is

$(s_L, s_H) = (\bar{U} + \beta_H e_L - \frac{p_L \beta_L \Delta e}{p_H - p_L}, \bar{U} + \beta_H e_L + \frac{(1-p_L)\beta_L \Delta e}{p_H - p_L})$

Again, (IR^{β_H}) binds, so the salesperson receives no surplus when the territory is hard.

$$\begin{aligned} s_L + p_H \Delta s - \beta_L e_H &= \bar{U} + \beta_H e_L - \frac{p_L \beta_L \Delta e}{p_H - p_L} + p_H \frac{\beta_L \Delta e}{p_H - p_L} - \beta_L e_H \\ &= \bar{U} + \beta_H e_L + \beta_L \Delta e - \beta_L e_H \\ &= \bar{U} + \Delta\beta e_L \end{aligned}$$

$\therefore$ the salesperson receives a surplus of $\Delta\beta e^{\beta_H}$ when the territory is easy.

Thus, under an efficient contract to induce $(e^{\beta_L}, e^{\beta_H})$ with $e^{\beta_L} \geq e^{\beta_H}$, the salesperson receives no surplus when the territory is hard and a surplus of $\Delta\beta e^{\beta_H}$ when it is easy. □

WEB APPENDIX D: ADDENDUM TO PROOF OF PROPOSITION 2

As noted in Appendix C, three questions remain to complete the proof of Proposition 2. These questions relate to the sequential nature of the two requests in Case 2. (Recall that the firm sets default constraints that result in $(e^{\beta_L}, e^{\beta_H}) = (e_H, 0)$ and sequential requirements $e_m^{rL} = (1-\gamma_L^0)(p_L -$

$p_0)\Delta m$ and $e_m^{rH} = (1-\gamma_L^2)(p_H - p_L)\Delta m$ for the manager to request constraints allowing (e_H, e_L) and (e_H, e_H), respectively.)

1) Does the sequential nature of these requests ever prevent the first request from being made when the second is desired (by the firm)?

Note that (e_H, e_H) is preferred when $\gamma_L < \gamma_L^2 < \gamma_L^0$, so (e_H, e_L) is preferred over $(e_H, 0)$. Therefore, if the second request is preferred, then the first request is always preferred as well. In other words, the sequential nature of the requests never prevents the first request from being made when the second request is desired.

2) Does the manager ever make a suboptimal first request in order to gain the opportunity to make the second one?

In other words, assuming that the manager foresees the opportunity for a second request, does considering the total effort exerted (and benefit gained) across both requests ever result in suboptimal behavior?

From Equation 2, the manager prefers this combined request if and only if $\gamma_L < \frac{(p_H-p_0)\Delta m-(e_m^{rL}+e_m^{rH})}{(p_H-p_0)\Delta m}$. Substituting for e_m^{rL} and e_m^{rH}, this gives:

$$\begin{aligned}
\gamma_L &< \frac{(p_H-p_0)\Delta m - \left(\left[1-\gamma_L^0\right]\left[p_L-p_0\right]\Delta m + \left[1-\gamma_L^2\right]\left[p_H-p_L\right]\Delta m\right)}{(p_H-p_0)\Delta m} \\
&< \frac{(p_H-p_0)-(1-\gamma_L^2)(p_H-p_0)+(\gamma_L^0-\gamma_L^2)(p_L-p_0)}{p_H-p_0} \\
\therefore\ \gamma_L &< \gamma_L^2 + \frac{(\gamma_L^0-\gamma_L^2)(p_L-p_0)}{p_H-p_0}
\end{aligned}$$

Since the firm prefers (e_H, e_H) only when $\gamma_L < \gamma_L^2$, this suggests a potential problem with the manager preferring to make both requests when $\gamma_L \in (\gamma_L^2, \gamma_L^2 + \frac{(\gamma_L^0-\gamma_L^2)(p_L-p_0)}{p_H-p_0})$. However:

$$\gamma_L^2 + \frac{(\gamma_L^0-\gamma_L^2)(p_L-p_0)}{p_H-p_0} = \gamma_L^0 - \frac{(\gamma_L^0-\gamma_L^2)(p_H-p_L)}{p_H-p_0} < \gamma_L^0$$

This indicates that when $\gamma_L \in (\gamma_L^2, \gamma_L^2 + \frac{(\gamma_L^0-\gamma_L^2)(p_L-p_0)}{p_H-p_0})$, the first request (for (e_H, e_L)) is, indeed, preferred. Having made that first request, the second request is then incentive-aligned, with the manager making it only when best for the firm. In other words, the combined effort required for

the two sequential requests is less than the firm would require of the manager if it were allowing her to go directly from $(e_H, 0)$ to (e_H, e_H), but this does not lead to suboptimal decisions.

3) Why is it necessary for the requests to be sequential?

To see why the sequential nature of the requests is necessary, consider the alternative. The firm could implement parallel requests, in which the manager could choose to *either* request constraints allowing (e_H, e_L) by completing requirements e_m^{rL} *or* request constraints allowing (e_H, e_H) by completing requirements e_m^{rH}. As above, the firm would set $e_m^{rL} = (1-\gamma_L^0)(p_L - p_0)\Delta m$ and $e_m^{rH} = (1-\gamma_L^2)(p_H - p_0)\Delta m$, so the manager would make the former request if and only if $\gamma_L < \gamma_L^0$ and the latter if and only if $\gamma_L < \gamma_L^2$, as desired.

Comparing these two choices, however, the manager prefers the latter if and only if:

$$
\begin{aligned}
& m_L + p_H \Delta m - e_m^{rH} > m_L + (\gamma_L p_H + [1-\gamma_L] p_L)\Delta m - e_m^{rL} \\
\iff \quad & \gamma_L < \frac{(p_H - p_L)\Delta m - (e_m^{rH} - e_m^{rL})}{(p_H - p_L)\Delta m} \\
\iff \quad & \gamma_L < \frac{(p_H - p_L)\Delta m - \left([1-\gamma_L^2][p_H - p_0]\Delta m - [1-\gamma_L^0][p_L - p_0]\Delta m\right)}{(p_H - p_L)\Delta m} \\
\iff \quad & \gamma_L < \frac{\gamma_L^2 (p_H - p_L) - [1-\gamma_L^2][p_L - p_0] + [1-\gamma_L^0][p_L - p_0]}{(p_H - p_L)} \\
\iff \quad & \gamma_L < \gamma_L^2 - \frac{(\gamma_L^0 - \gamma_L^2)(p_L - p_0)}{(p_H - p_L)}
\end{aligned}
$$

Therefore, when $\gamma_L \in (\gamma_L^2 - \frac{(\gamma_L^0 - \gamma_L^2)(p_L - p_0)}{(p_H - p_L)}, \gamma_L^2)$, the manager chooses to request (e_H, e_L) while the firm prefers (e_H, e_H). Implementing the requests sequentially resolves this issue. □

WEB APPENDIX E: PROOF OF LEMMA 4

Given a contract (s_L, s_H), the salesperson's choice is as in Step 6 of the main model. He is willing to accept if (IR) is satisfied for some positive effort and he prefers $\begin{cases} e_L & \text{if } \beta \Delta e \geq (p_H - p_L)\Delta s \\ e_H & \text{if } \beta \Delta e \leq (p_H - p_L)\Delta s \end{cases}$

Given a menu of contracts $\{(s_L^1, s_H^1), (s_L^2, s_H^2), ..., (s_L^n, s_H^n)\}$, he chooses the one that maximizes his expected utility, $E[U(s_L^i, s_H^i)] = s_L^i + p(e)\Delta s^i - \beta e$, where e is his optimal effort given (s_L^i, s_H^i). If none of the contracts satisfy his (IR) constraint, then he exits.

As in the main model, a menu of contracts will induce efforts $(e^{\beta_L}, e^{\beta_H})$ in an easy and hard territory, respectively, where $e^{\beta_L}, e^{\beta_H} \in \{0, e_L, e_H\}$. Since there are only two territory types, the menu need not contain more than two options. Thus, the firm chooses a menu $\{(s_L^{\beta_L}, s_H^{\beta_L}), (s_L^{\beta_H}, s_H^{\beta_H})\}$ to maximize its expected profit given its belief γ_L^F, where $(s_L^{\beta}, s_H^{\beta})$ is the contract intended to be chosen by the salesperson in a territory of type β. (If $e^{\beta_H} = 0$, then all contracts are designed to be rejected if $\beta = \beta_H$.) It is possible for the optimal 'menu' to be a single contract, so $(s_L^{\beta_L}, s_H^{\beta_L}) = (s_L^{\beta_H}, s_H^{\beta_H})$.

Extension of Lemma 3

Case a: $e^{\beta_H} > 0$ The salesperson is intended to accept a contract in either territory type. The firm's problem can be expressed as:

$$\max_{(s_L^{\beta_L}, \Delta s^{\beta_L}), (s_L^{\beta_H}, \Delta s^{\beta_H})} E[\pi] = \gamma_L^F \left[x_L - m_L - s_L^{\beta_L} + p(e^{\beta_L})(\Delta x - \Delta m - \Delta s^{\beta_L}) \right] +$$

$$[1 - \gamma_L^F] \left[x_L - m_L - s_L^{\beta_H} + p(e^{\beta_H})(\Delta x - \Delta m - \Delta s^{\beta_H}) \right]$$

$$\text{s.t. } s_L^{\beta_L} + p(e^{\beta_L})\Delta s^{\beta_L} - \beta_L e^{\beta_L} \geq \bar{U} \qquad (\text{IR}^{\beta_L})$$

$$s_L^{\beta_H} + p(e^{\beta_H})\Delta s^{\beta_H} - \beta_H e^{\beta_H} \geq \bar{U} \qquad (\text{IR}^{\beta_H})$$

$$s_L^{\beta_L} + p(e^{\beta_L})\Delta s^{\beta_L} - \beta_L e^{\beta_L} \geq s_L^{\beta_L} + p(e)\Delta s^{\beta_L} - \beta_L e \quad \forall e \in \{e_L, e_H\} \qquad (\text{IC}^{\beta_L})$$

$$s_L^{\beta_H} + p(e^{\beta_H})\Delta s^{\beta_H} - \beta_H e^{\beta_H} \geq s_L^{\beta_H} + p(e)\Delta s^{\beta_H} - \beta_H e \quad \forall e \in \{e_L, e_H\} \qquad (\text{IC}^{\beta_H})$$

$$s_L^{\beta_L} + p(e^{\beta_L})\Delta s^{\beta_L} - \beta_L e^{\beta_L} \geq s_L^{\beta_H} + p(e)\Delta s^{\beta_H} - \beta_L e \quad \forall e \in \{e_L, e_H\} \qquad (\text{SS}^{\beta_L})$$

$$s_L^{\beta_H} + p(e^{\beta_H})\Delta s^{\beta_H} - \beta_H e^{\beta_H} \geq s_L^{\beta_L} + p(e)\Delta s^{\beta_L} - \beta_H e \quad \forall e \in \{e_L, e_H\} \qquad (\text{SS}^{\beta_H})$$

$$s_L^{\beta_L} \geq 0 \qquad (\text{LL}^{\beta_L})$$

$$s_L^{\beta_H} \geq 0 \qquad (\text{LL}^{\beta_H})$$

where the (SS) (self-selection) constraints ensure that the salesperson chooses the contract intended for his territory type.

I begin with an adapted version of this problem, omitting the (LL) constraints. I then show that the optimal solution to that problem can be achieved without violating those additional constraints, making it a solution to the complete problem.

Claim: (IR$^{\beta_H}$) binds.

Proof: At least one of the (IR) constraints must bind, because otherwise the firm can lower $s_L^{\beta_L}$ and $s_L^{\beta_H}$ equally, increasing the objective function without violating any constraints.

Suppose (IR$^{\beta_L}$) binds. Then (SS$^{\beta_L}$) implies that

$$\begin{aligned} \bar{U} &\geq s_L^{\beta_H} + p(e^{\beta_H})\Delta s^{\beta_H} - \beta_L e^{\beta_H} \\ &> s_L^{\beta_H} + p(e^{\beta_H})\Delta s^{\beta_H} - \beta_H e^{\beta_H} \\ \therefore \bar{U} &> \bar{U} \qquad \text{by (IR}^{\beta_H}) \end{aligned}$$

This is a contradiction, so (IR$^{\beta_H}$) must bind. □

∴ When the territory is hard, the salesperson receives no surplus, as in Lemma 3.

Claim: When the territory is easy, the salesperson receives an expected utility surplus of $\Delta\beta e^{\beta_H}$.

Proof: Substituting (IR$^{\beta_H}$) into (SS$^{\beta_L}$) gives $s_L^{\beta_L} + p(e^{\beta_L})\Delta s^{\beta_L} - \beta_L e^{\beta_L} \geq \bar{U} + \Delta\beta e^{\beta_H}$. So (IR$^{\beta_L}$) has slack (i.e., the salesperson receives rent in an easy territory) of *at least* $\Delta\beta e^{\beta_H}$.

(SS$^{\beta_L}$) must bind for some $e \in \{e_L, e_H\}$, because otherwise the firm can lower $s_L^{\beta_L}$ and increase the objective function without violating any constraints.

$$\therefore s_L^{\beta_L} + p(e^{\beta_L})\Delta s^{\beta_L} - \beta_L e^{\beta_L} = \max\{s_L^{\beta_H} + p_L\Delta s^{\beta_H} - \beta_L e_L, s_L^{\beta_H} + p_H\Delta s^{\beta_H} - \beta_L e_H\}$$

Subcase a.i: $\boldsymbol{e^{\beta_H} = e_L}$ In this case, the firm can set $\Delta s^{\beta_H} = 0$, because the salesperson is expected to exert the minimum effort. So, by (IR$^{\beta_H}$), $s_L^{\beta_H} = s_H^{\beta_H} = \bar{U} + \beta_H e_L$. From above, then,

$$\begin{aligned} s_L^{\beta_L} + p(e^{\beta_L})\Delta s^{\beta_L} - \beta_L e^{\beta_L} &= \max\{\bar{U} + \beta_H e_L - \beta_L e_L, \bar{U} + \beta_H e_L - \beta_L e_H\} \\ \therefore s_L^{\beta_L} + p(e^{\beta_L})\Delta s^{\beta_L} - \beta_L e^{\beta_L} &= \bar{U} + \Delta\beta e_L \end{aligned}$$

So, under this menu, the salesperson receives rent of $\Delta\beta e^{\beta_H}$ if the territory is easy. Therefore, since an efficient menu of contracts for a given $(e^{\beta_L}, e^{\beta_H})$ minimizes the rent paid in each territory type, *any* efficient menu for (e^{β_L}, e_L) must offer rent of $\Delta\beta e^{\beta_H}$ if the territory is easy.

$s_L^{\beta_H} = \bar{U} + \beta_H e_L > 0$, so imposing (LL^{β_H}) does not affect this solution.

If $e^{\beta_L} = e_L$, the firm can set $\Delta s^{\beta_L} = 0$, so $s_L^{\beta_L} = \bar{U} + \beta_H e_L > 0$.

If $e^{\beta_L} = e_H$, the firm can set $\Delta s^{\beta_L} = \frac{\beta_L \Delta e}{p_H - p_L}$, so $s_L^{\beta_L} = \bar{U} + \beta_H e_L - \frac{\beta_L p_L \Delta e}{p_H - p_L}$. By assumption, $\bar{U} \geq \beta_H(\frac{p_L e_H - p_H e_L}{p_H - p_L})$, so

$$\begin{aligned} s_L^{\beta_L} &\geq \beta_H(\frac{p_L e_H - p_H e_L}{p_H - p_L}) + \beta_H e_L - \frac{\beta_L p_L \Delta e}{p_H - p_L} \\ \therefore &\geq \frac{1}{p_H - p_L}(\beta_H[p_L e_H - p_H e_L] + \beta_H e_L[p_H - p_L] - \beta_L p_L \Delta e) \\ \therefore &\geq \frac{\Delta\beta p_L \Delta e}{p_H - p_L} \\ \therefore s_L^{\beta_L} &> 0 \end{aligned}$$

$\therefore$ imposing (LL^{β_L}) does not prevent the firm from implementing this solution for either e^{β_L}.

Subcase a.ii: $\boldsymbol{e^{\beta_H} = e_H}$ $\therefore e^{\beta_L} = e_H$, because $e^{\beta_L} \geq e^{\beta_H}$.

Suppose (SS^{β_L}) binds for $e = e_L$. Then, substituting in (IR^{β_H}) gives

$s_L^{\beta_L} + p_H \Delta s^{\beta_L} - \beta_L e_H = \bar{U} - (p_H - p_L)\Delta s^{\beta_H} + \beta_H e_H - \beta_L e_L$

From (IC^{β_H}), $(p_H - p_L)\Delta s^{\beta_H} \geq \beta_H e_H - \beta_H e_L$, so

$$\begin{aligned} s_L^{\beta_L} + p_H \Delta s^{\beta_L} - \beta_L e_H &\leq \bar{U} - (\beta_H e_H - \beta_H e_L) + \beta_H e_H - \beta_L e_L \\ &\leq \bar{U} + \Delta\beta e_L \\ \therefore s_L^{\beta_L} + p_H \Delta s^{\beta_L} - \beta_L e_H &< \bar{U} + \Delta\beta e_H \end{aligned}$$

This contradicts the finding above that (IR^{β_L}) has slack of at least $\Delta\beta e^{\beta_H}$. Therefore, (SS^{β_L}) cannot bind for $e = e_L$, so it must bind for $e = e_H$. Substituting in (IR^{β_H}) gives

$$s_L^{\beta_L} + p_H \Delta s^{\beta_L} - \beta_L e_H = \bar{U} + \Delta\beta e_H$$

So, the salesperson receives rent of $\Delta\beta e^{\beta_H}$ if the territory is easy.

Again, since an efficient menu of contracts for a given $(e^{\beta_L}, e^{\beta_H})$ minimizes the rent paid in each territory type, *any* efficient menu for (e_H, e_H) must offer rent of $\Delta\beta e^{\beta_H}$ if the territory is easy. This can be achieved with $\Delta s = \frac{\beta_H \Delta e}{p_H - p_L}$ and $s_L = \bar{U} + \beta_H e_H - \frac{\beta_H p_H \Delta e}{p_H - p_L}$ for *both* territory types (i.e., a menu composed of a single contract).

Since $\bar{U} \geq \beta_H(\frac{p_L e_H - p_H e_L}{p_H - p_L})$,

$$\begin{aligned} s_L &\geq \beta_H(\frac{p_L e_H - p_H e_L}{p_H - p_L}) + \beta_H e_H - \frac{\beta_H p_H \Delta e}{p_H - p_L} \\ \therefore &\geq \frac{\beta_H}{p_H - p_L}(p_L e_H - p_H e_L + e_H[p_H - p_L] - p_H \Delta e) \\ \therefore s_L &\geq 0 \end{aligned}$$

$\therefore$ this solution can be achieved without violating either (LL) constraint. □

Therefore, Lemma 3 always holds in Case a.

Case b: $e^{\beta_H} = 0$ This case allows for the possibility that, even under the menu of contracts approach, the firm might prefer to allow the salesperson to exit when his territory is hard. As noted in Lal and Staelin (1986), this occurs when the expected cost of losing the salesperson from a hard territory is outweighed by the rent paid in an easy one. Thus, the firm offers a 'menu' consisting of a single contract, designed such that the salesperson will accept only if the territory is easy.

Since the salesperson will exit when the territory is hard, the contract can be optimized as though it is known to be easy. In other words, the firm's problem reduces to a standard principal-agent model, with the following well-established solution:

To induce $(e_L, 0)$, by (IR$^{\beta_L}$), the firm sets $s_L^{\beta_L} = s_H^{\beta_L} = \bar{U} + \beta_L e_L$. Under this contract, (IR$^{\beta_L}$) binds, so the salesperson accepts and earns a surplus of 0 ($= \Delta\beta e^{\beta_H}$) if the territory is easy. Conversely, (IR$^{\beta_H}$) is not satisfied for any e^{β_H}, so he exits (and earns 0 surplus) if it is hard. Clearly, $s_L^{\beta_L} = \bar{U} + \beta_L e_L > 0$, so this solution does not violate the (LL) constraints.

To induce $(e_H, 0)$, the firm offers

$$(s_L, s_H) = \left(\bar{U} + \beta_L \left[e_H - p_H \frac{\Delta e}{p_H - p_L}\right], \bar{U} + \beta_L \left[e_H + (1 - p_H) \frac{\Delta e}{p_H - p_L}\right]\right)$$

$\therefore$ $(p_H - p_L)\Delta s = \beta_L \Delta e$ and $s_L + p_H \Delta s - \beta_L e_H = \bar{U}$, so the salesperson accepts and chooses e_H, earning 0 surplus, if the territory is easy. If it is hard, then the salesperson's expected utility if he stays and exerts $e \in \{e_L, e_H\}$ is

$$
\begin{aligned}
s_L + p(e)\Delta s - \beta_H e &= \bar{U} + \beta_L \left[e_H - p_H \frac{\Delta e}{p_H - p_L} \right] + p(e)\beta_L \frac{\Delta e}{p_H - p_L} - \beta_H e \\
&= \bar{U} + \beta_L \left(e_H - [p_H - p(e)] \frac{\Delta e}{p_H - p_L} \right) - \beta_H e \\
&= \bar{U} + \beta_L e - \beta_H e \\
\therefore\ s_L + p(e)\Delta s - \beta_H e &< \bar{U}
\end{aligned}
$$

Therefore, if the territory is hard, then the salesperson exits and earns 0 surplus.

Since $\bar{U} \geq \beta_H (\frac{p_L e_H - p_H e_L}{p_H - p_L}) \geq 0$,

$$
\begin{aligned}
s_L &\geq \beta_H (\frac{p_L e_H - p_H e_L}{p_H - p_L}) + \beta_L \left[e_H - p_H \frac{\Delta e}{p_H - p_L} \right] \\
\therefore &\geq \frac{1}{p_H - p_L} (\beta_H [p_L e_H - p_H e_L] + \beta_L [(p_H - p_L) e_H - p_H \Delta e]) \\
\therefore &\geq \frac{\Delta \beta}{p_H - p_L} (p_L e_H - p_H e_L) \\
\therefore\ s_L &\geq 0
\end{aligned}
$$

$\therefore$ this solution can be achieved without violating either (LL) constraint.

Therefore, Lemma 3 holds in all cases. □

The proof of Proposition 1 extends directly, by replacing "efficient contract" with "efficient menu of contracts". □

WEB APPENDIX F: DETAILED PROOF OF PROPOSITION 3

First, consider the firm's expected profits using a menu of contracts. For the optimal design of the menu, see the proof of Lemma 4 in Web Appendix E.

When deciding whether to accept her contract (m_L, m_H), the manager anticipates the menu that the firm will offer the salesperson and the resulting $(e^{\beta_L}, e^{\beta_H})$, so she accepts if and only if:

$$E[U_m|(e^{\beta_L}, e^{\beta_H})] = m_L + \left(p(e^{\beta_H}) + \gamma_L \left[p(e^{\beta_L}) - p(e^{\beta_H})\right]\right) \Delta m \geq \bar{U}_m$$

Since the firm does not observe the manager's belief when designing her contract, it must ensure that she will accept for *any* $\gamma_L \in (0,1)$. Her expected utility weakly increases with γ_L, so it is necessary and sufficient to ensure that she accepts for $\gamma_L \to 0$. Therefore, her contract must satisfy

$$m_L + p(e^{\beta_H})\Delta m \geq \bar{U}_m \tag{W1}$$

Given that constraint, the firm maximizes its expected profit. From Lemma 4, that is given by:

$$E[\pi(e^{\beta_L}, e^{\beta_H})] = x_L - m_L + \gamma_L^F \left[p(e^{\beta_L})(\Delta x - \Delta m) - (\bar{U} + \beta_L e^{\beta_L} + \Delta\beta e^{\beta_H})\right] +$$
$$(1 - \gamma_L^F)\left[p(e^{\beta_H})(\Delta x - \Delta m) - \mathbb{1}_{e^{\beta_H}>0}(\bar{U} + \beta_H e^{\beta_H})\right]$$

It is clear that, for any $(e^{\beta_L}, e^{\beta_H})$, the firm prefers to minimize m_L, subject to Inequality W1.

$$\therefore m_L = \bar{U}_m - p(e^{\beta_H})\Delta m$$

Therefore, the firm's expected profit given an efficient menu of contracts to induce $(e^{\beta_L}, e^{\beta_H})$ is

$$E[\pi(e^{\beta_L}, e^{\beta_H})] = x_L - [\bar{U}_m - p(e^{\beta_H})\Delta m] + \gamma_L^F \left[p(e^{\beta_L})(\Delta x - \Delta m) - (\bar{U} + \beta_L e^{\beta_L} + \Delta\beta e^{\beta_H})\right]$$
$$+(1 - \gamma_L^F)\left[p(e^{\beta_H})(\Delta x - \Delta m) - \mathbb{1}_{e^{\beta_H}>0}(\bar{U} + \beta_H e^{\beta_H})\right] \tag{W2}$$

By Lemma 4, the relevant cases are the same as under the request mechanism, as given in the proof of Proposition 2 (Appendix C).

Comparison of expected profits

Case 1: $\boldsymbol{(p_L - p_0)(\Delta x - \Delta m) < \bar{U} + \beta_H e_L}$ ***OR*** $\boldsymbol{\gamma_L^0 < \gamma_L^2}$ The firm prefers to induce $(e^{\beta_L}, e^{\beta_H}) = (e_H, e_H)$ if and only if $\gamma_L < \gamma_L^1 = \frac{(p_H - p_0)(\Delta x - \Delta m) - (\bar{U} + \beta_H e_H)}{(p_H - p_0)(\Delta x - \Delta m) - (\bar{U} + \beta_L e_H)}$ and $(e_H, 0)$ otherwise.

Under the menu of contracts, the firm chooses based on its own belief, $\gamma_L^F = 0.5$. From Equation

W2, its expected profits are:

$$E[\pi|\,\text{menu}, \gamma_L^1 < 0.5] = x_L + p_0\Delta x - \bar{U}_m + \frac{1}{2}\left[(p_H - p_0)(\Delta x - \Delta m) - (\bar{U} + \beta_L e_H)\right]$$

$$E[\pi|\,\text{menu}, \gamma_L^1 > 0.5] = x_L + p_H\Delta x - (\bar{U}_m + \bar{U} + \beta_H e_H)$$

Under the request mechanism, the manager chooses based on *her* belief, which is unknown to the firm. Therefore, the expected profit is:

$$E[\pi|\,\text{req}] = \int_0^{\gamma_L^1} E[\pi(e_H, e_H)]f(\gamma_L)\mathrm{d}\gamma_L + \int_{\gamma_L^1}^1 E[\pi(e_H, 0)]f(\gamma_L)\mathrm{d}\gamma_L$$

The manager's signal $Q \sim U(0.5, 1) \implies \gamma_L \sim U(0, 1)$, so $f(\gamma_L) = 1$ for all values of $\gamma_L \in (0, 1)$.

From Equation 3:

$$E[\pi(e_H, 0)] = x_L - m_L + p_0(\Delta x - \Delta m) + \gamma_L\left[(p_H - p_0)(\Delta x - \Delta m) - (\bar{U} + \beta_L e_H)\right]$$

$$E[\pi(e_H, e_H)] = x_L - m_L + p_H(\Delta x - \Delta m) - (\bar{U} + \beta_H e_H)$$

Substituting in $m_L = \bar{U}_m - \left(p_0 + \gamma_L^1[p_H - p_0]\right)\Delta m$ from Table 3:

$$E[\pi(e_H, 0)] = x_L + p_0\Delta x - \bar{U}_m + \gamma_L^1[p_H - p_0]\Delta m + \gamma_L\left[(p_H - p_0)(\Delta x - \Delta m) - (\bar{U} + \beta_L e_H)\right]$$

$$E[\pi(e_H, e_H)] = x_L + p_H\Delta x - \bar{U}_m - (1 - \gamma_L^1)(p_H - p_0)\Delta m - (\bar{U} + \beta_H e_H)$$

$$\therefore E[\pi|\,\text{req}] = \int_0^{\gamma_L^1}\left[x_L + p_H\Delta x - \bar{U}_m - (1 - \gamma_L^1)(p_H - p_0)\Delta m - (\bar{U} + \beta_H e_H)\right]\mathrm{d}\gamma_L +$$

$$\int_{\gamma_L^1}^1\left[x_L + p_0\Delta x - \bar{U}_m + \gamma_L^1[p_H - p_0]\Delta m + \gamma_L\left[(p_H - p_0)(\Delta x - \Delta m) - (\bar{U} + \beta_L e_H)\right]\right]\mathrm{d}\gamma_L$$

$$= \gamma_L^1\left[x_L + p_H\Delta x - \bar{U}_m - (1 - \gamma_L^1)(p_H - p_0)\Delta m - (\bar{U} + \beta_H e_H)\right] +$$

$$(1 - \gamma_L^1)\left(x_L + p_0\Delta x - \bar{U}_m + \gamma_L^1[p_H - p_0]\Delta m + \frac{1 + \gamma_L^1}{2}\left[(p_H - p_0)(\Delta x - \Delta m) - (\bar{U} + \beta_L e_H)\right]\right)$$

$$\therefore E[\pi|\,\text{req}] = x_L + p_0\Delta x - \bar{U}_m + \gamma_L^1\left[(p_H - p_0)\Delta x - (\bar{U} + \beta_H e_H)\right] +$$

$$(1 - \gamma_L^1)\frac{1 + \gamma_L^1}{2}\left[(p_H - p_0)(\Delta x - \Delta m) - (\bar{U} + \beta_L e_H)\right]$$

Now the two approaches can be compared. First, when $\gamma_L^1 < 0.5$:

$$E[\pi|\,\text{req}]{-}E[\pi|\,\text{menu},\gamma_L^1 < 0.5] = x_L + p_0\Delta x - \bar{U}_m + \gamma_L^1\left[(p_H - p_0)\Delta x - (\bar{U} + \beta_H e_H)\right] +$$

$$(1-\gamma_L^1)\left(\frac{1+\gamma_L^1}{2}\left[(p_H - p_0)(\Delta x - \Delta m) - (\bar{U} + \beta_L e_H)\right]\right) -$$

$$\left(x_L + p_0\Delta x - \bar{U}_m + \frac{1}{2}\left[(p_H - p_0)(\Delta x - \Delta m) - (\bar{U} + \beta_L e_H)\right]\right)$$

$$= \gamma_L^1\left[(p_H - p_0)\Delta x - (\bar{U} + \beta_H e_H) - \frac{\gamma_L^1}{2}\left[(p_H - p_0)(\Delta x - \Delta m) - (\bar{U} + \beta_L e_H)\right]\right]$$

$$= \gamma_L^1\left(\left[1 - \frac{\gamma_L^1}{2}\right]\left[(p_H - p_0)(\Delta x - \Delta m) - (\bar{U} + \beta_L e_H)\right] - \Delta\beta e_H\right) + \gamma_L^1(p_H - p_0)\Delta m$$

$$= \gamma_L^1\left[(p_H - p_0)(\Delta x - \Delta m) - (\bar{U} + \beta_L e_H)\right]\left(\left[1 - \frac{\gamma_L^1}{2}\right] - [1 - \gamma_L^1]\right) + \gamma_L^1(p_H - p_0)\Delta m$$

$$\therefore\ E[\pi|\,\text{req}]{-}E[\pi|\,\text{menu},\gamma_L^1 < 0.5] = \gamma_L^1\left(\frac{\gamma_L^1}{2}\left[(p_H - p_0)(\Delta x - \Delta m) - (\bar{U} + \beta_L e_H)\right] + (p_H - p_0)\Delta m\right)$$

As shown in (Appendix B), $(p_H - p_0)(\Delta x - \Delta m) > \bar{U} + \beta_H e_H > \bar{U} + \beta_L e_H$. Therefore, $E[\pi|\,\text{req}] - E[\pi|\,\text{menu},\gamma_L^1 < 0.5] > 0$, so the firm prefers the request mechanism in Case 1 when its best menu of contracts induces the salesperson to exit if his territory is hard.

Next, consider $\gamma_L^1 > 0.5$:

$$E[\pi|\,\text{req}]{-}E[\pi|\,\text{menu},\gamma_L^1 > 0.5] = x_L + p_0\Delta x - \bar{U}_m + \gamma_L^1\left[(p_H - p_0)\Delta x - (\bar{U} + \beta_H e_H)\right] +$$

$$(1-\gamma_L^1)\left(\frac{1+\gamma_L^1}{2}\left[(p_H - p_0)(\Delta x - \Delta m) - (\bar{U} + \beta_L e_H)\right]\right) - \left(x_L + p_H\Delta x - (\bar{U}_m + \bar{U} + \beta_H e_H)\right)$$

$$= (1-\gamma_L^1)\left(\left[-\frac{1-\gamma_L^1}{2}\right]\left[(p_H - p_0)(\Delta x - \Delta m) - (\bar{U} + \beta_L e_H)\right] + \Delta\beta e_H\right) - (1-\gamma_L^1)(p_H - p_0)\Delta m$$

$$= (1-\gamma_L^1)\left[(p_H - p_0)(\Delta x - \Delta m) - (\bar{U} + \beta_L e_H)\right]\left(\left[-\frac{1-\gamma_L^1}{2}\right] + [1-\gamma_L^1]\right) - (1-\gamma_L^1)(p_H - p_0)\Delta m$$

$$= (1-\gamma_L^1)\left(\frac{1-\gamma_L^1}{2}\left[(p_H - p_0)(\Delta x - \Delta m) - (\bar{U} + \beta_L e_H)\right] - (p_H - p_0)\Delta m\right)$$

$$E[\pi|\,\text{req}]{-}E[\pi|\,\text{menu},\gamma_L^1 > 0.5] = (1-\gamma_L^1)\left(\frac{\Delta\beta e_H}{2} - (p_H - p_0)\Delta m\right)$$

This is positive if and only if: $\Delta m < \frac{\Delta\beta e_H}{2(p_H - p_0)}$

Therefore, when the firm's best menu of contracts does *not* induce the salesperson to exit in Case 1, the request mechanism is preferred as long as Δm is not too large ($< \frac{\Delta\beta e_H}{2(p_H - p_0)}$).

Case 2: Otherwise $\left((p_L - p_0)(\Delta x - \Delta m) > \bar{U} + \beta_H e_L \text{ AND } \gamma_L^0 > \gamma_L^2\right)$ The firm considers all three of $(e^{\beta_L}, e^{\beta_H}) = (e_H, 0), (e_H, e_L)$ and (e_H, e_H), choosing as follows: $\begin{cases} (e_H, 0) & \text{if } \gamma_L^0 < \gamma_L \\ (e_H, e_L) & \text{if } \gamma_L \in (\gamma_L^2, \gamma_L^0) \\ (e_H, e_H) & \text{if } \gamma_L < \gamma_L \end{cases}$

where $\gamma_L^0 = \frac{(p_L - p_0)(\Delta x - \Delta m) - (\bar{U} + \beta_H e_L)}{(p_L - p_0)(\Delta x - \Delta m) - (\bar{U} + \beta_L e_L)}$, $\gamma_L^1 = \frac{(p_H - p_0)(\Delta x - \Delta m) - (\bar{U} + \beta_H e_H)}{(p_H - p_0)(\Delta x - \Delta m) - (\bar{U} + \beta_L e_H)}$, and $\gamma_L^2 = \frac{(p_H - p_L)(\Delta x - \Delta m) - \beta_H \Delta e}{(p_H - p_L)(\Delta x - \Delta m) - \beta_L \Delta e}$.

Again, under the menu of contracts, the firm chooses based on its own belief, $\gamma_L^F = 0.5$. From Equation W2, its expected profits are:

$$E[\pi|\,\text{menu}, \gamma_L^0 < 0.5] = x_L + p_0 \Delta x - \bar{U}_m + \frac{1}{2}\left[(p_H - p_0)(\Delta x - \Delta m) - (\bar{U} + \beta_L e_H)\right]$$

$$E[\pi|\,\text{menu}, 0.5 \in (\gamma_L^2, \gamma_L^0)] = x_L + p_L \Delta x - \bar{U}_m - (\bar{U} + \beta_H e_L) + \frac{1}{2}\left[(p_H - p_L)(\Delta x - \Delta m) - \beta_L \Delta e\right]$$

$$E[\pi|\,\text{menu}, \gamma_L^2 > 0.5] = x_L + p_H \Delta x - \bar{U}_m - (\bar{U} + \beta_H e_H)$$

Under the request mechanism, the firm's expected profit is:

$$E[\pi|\,\text{req}] = \int_0^{\gamma_L^2} E[\pi(e_H, e_H)] f(\gamma_L) \mathrm{d}\gamma_L + \int_{\gamma_L^2}^{\gamma_L^0} E[\pi(e_H, e_L)] f(\gamma_L) \mathrm{d}\gamma_L + \int_{\gamma_L^0}^{1} E[\pi(e_H, 0)] f(\gamma_L) \mathrm{d}\gamma_L$$

From Equation 3:

$$E[\pi(e_H, 0)] = x_L - m_L + p_0(\Delta x - \Delta m) + \gamma_L\left[(p_H - p_0)(\Delta x - \Delta m) - (\bar{U} + \beta_L e_H)\right]$$

$$E[\pi(e_H, e_L)] = x_L - m_L + p_L(\Delta x - \Delta m) - (\bar{U} + \beta_H e_L) + \gamma_L\left[(p_H - p_L)(\Delta x - \Delta m) - \beta_L \Delta e\right]$$

$$E[\pi(e_H, e_H)] = x_L - m_L + p_H(\Delta x - \Delta m) - (\bar{U} + \beta_H e_H)$$

Therefore,

$$
\begin{aligned}
E[\pi|\,\text{req}] = &\int_0^{\gamma_L^2} \left(x_L - m_L + p_H(\Delta x - \Delta m) - (\bar{U} + \beta_H e_H)\right) f(\gamma_L)\,\mathrm{d}\gamma_L + \\
&\int_{\gamma_L^2}^{\gamma_L^0} \left(x_L - m_L + p_L(\Delta x - \Delta m) - (\bar{U} + \beta_H e_L) + \gamma_L\left[(p_H - p_L)(\Delta x - \Delta m) - \beta_L \Delta e\right]\right) f(\gamma_L)\mathrm{d}\gamma_L + \\
&\int_{\gamma_L^0}^{1} \left(x_L - m_L + p_0(\Delta x - \Delta m) + \gamma_L\left[(p_H - p_0)(\Delta x - \Delta m) - (\bar{U} + \beta_L e_H)\right]\right) f(\gamma_L)\mathrm{d}\gamma_L \\
&= \gamma_L^2 \left(x_L - m_L + p_H(\Delta x - \Delta m) - (\bar{U} + \beta_H e_H)\right) + \\
&(\gamma_L^0 - \gamma_L^2)\left(x_L - m_L + p_L(\Delta x - \Delta m) - (\bar{U} + \beta_H e_L) + \frac{\gamma_L^0 + \gamma_L^2}{2}\left[(p_H - p_L)(\Delta x - \Delta m) - \beta_L \Delta e\right]\right) + \\
&(1 - \gamma_L^0)\left(x_L - m_L + p_0(\Delta x - \Delta m) + \frac{1 + \gamma_L^0}{2}\left[(p_H - p_0)(\Delta x - \Delta m) - (\bar{U} + \beta_L e_H)\right]\right) \\
&= x_L - m_L + p_0(\Delta x - \Delta m) + \gamma_L^0\left[(p_L - p_0)(\Delta x - \Delta m) - (\bar{U} + \beta_H e_L)\right] + \\
&\quad \gamma_L^2\left[(p_H - p_L)(\Delta x - \Delta m) - \beta_H \Delta e\right] + (\gamma_L^0 - \gamma_L^2)\frac{\gamma_L^0 + \gamma_L^2}{2}\left[(p_H - p_L)(\Delta x - \Delta m) - \beta_L \Delta e\right] + \\
&\quad (1 - \gamma_L^0)\frac{1 + \gamma_L^0}{2}\left[(p_H - p_0)(\Delta x - \Delta m) - (\bar{U} + \beta_L e_H)\right]
\end{aligned}
$$

Substituting in $m_L = \bar{U}_m - \left(p_0 + \gamma_L^0[p_L - p_0] + \gamma_L^2[p_H - p_L]\right)\Delta m$ from Table 3:

$$
\begin{aligned}
\therefore\ E[\pi|\,\text{req}] = &\ x_L + p_0 \Delta x - \bar{U}_m + \gamma_L^0\left[(p_L - p_0)\Delta x - (\bar{U} + \beta_H e_L)\right] + \gamma_L^2\left[(p_H - p_L)\Delta x - \beta_H \Delta e\right] + \\
&(\gamma_L^0 - \gamma_L^2)\frac{\gamma_L^0 + \gamma_L^2}{2}\left[(p_H - p_L)(\Delta x - \Delta m) - \beta_L \Delta e\right] + \\
&(1 - \gamma_L^0)\frac{1 + \gamma_L^0}{2}\left[(p_H - p_0)(\Delta x - \Delta m) - (\bar{U} + \beta_L e_H)\right]
\end{aligned}
$$

Now the two approaches can be compared. First, when $\gamma_L^1 < 0.5$:

$$
\begin{aligned}
E[\pi|\,\text{req}]-E[\pi|\,\text{menu},\gamma_L^0 < 0.5] &= x_L + p_0\Delta x - \bar{U}_m + \gamma_L^0\left[(p_L - p_0)\Delta x - (\bar{U} + \beta_H e_L)\right] + \\
&\gamma_L^2\left[(p_H - p_L)\Delta x - \beta_H \Delta e\right] + (\gamma_L^0 - \gamma_L^2)\frac{\gamma_L^0 + \gamma_L^2}{2}\left[(p_H - p_L)(\Delta x - \Delta m) - \beta_L \Delta e\right] + \\
&(1-\gamma_L^0)\frac{1+\gamma_L^0}{2}\left[(p_H - p_0)(\Delta x - \Delta m) - (\bar{U} + \beta_L e_H)\right] - \\
&\left(x_L + p_0\Delta x - \bar{U}_m + \frac{1}{2}\left[(p_H - p_0)(\Delta x - \Delta m) - (\bar{U} + \beta_L e_H)\right]\right) \\
= \gamma_L^0\left[(p_L - p_0)\Delta x - (\bar{U} + \beta_H e_L)\right] + \gamma_L^2\left[(p_H - p_L)\Delta x - \beta_H\Delta e\right] + \\
(\gamma_L^0 - \gamma_L^2)\frac{\gamma_L^0 + \gamma_L^2}{2}\left[(p_H - p_L)(\Delta x - \Delta m) - \beta_L\Delta e\right] - \frac{(\gamma_L^0)^2}{2}\left[(p_H - p_0)(\Delta x - \Delta m) - (\bar{U} + \beta_L e_H)\right] \\
= \left[\gamma_L^0(p_L - p_0) + \gamma_L^2(p_H - p_L)\right]\Delta m + \gamma_L^0\left[(p_L - p_0)(\Delta x - \Delta m) - (\bar{U} + \beta_H e_L)\right] + \\
\gamma_L^2\left[(p_H - p_L)(\Delta x - \Delta m) - \beta_H\Delta e\right] - \frac{(\gamma_L^0)^2}{2}\left[(p_L - p_0)(\Delta x - \Delta m) - (\bar{U} + \beta_L e_L)\right] - \\
\frac{(\gamma_L^2)^2}{2}\left[(p_H - p_L)(\Delta x - \Delta m) - \beta_L\Delta e\right] \\
= \left[\gamma_L^0(p_L - p_0) + \gamma_L^2(p_H - p_L)\right]\Delta m + \gamma_L^0\left(\left[1 - \frac{\gamma_L^0}{2}\right]\left[(p_L - p_0)(\Delta x - \Delta m) - (\bar{U} + \beta_L e_L)\right] - \Delta\beta e_L\right) + \\
\gamma_L^2\left(\left[1 - \frac{\gamma_L^2}{2}\right]\left[(p_H - p_L)(\Delta x - \Delta m) - \beta_L\Delta e\right] - \Delta\beta\Delta e\right) \\
= \left[\gamma_L^0(p_L - p_0) + \gamma_L^2(p_H - p_L)\right]\Delta m + \gamma_L^0\left[(p_L - p_0)(\Delta x - \Delta m) - (\bar{U} + \beta_L e_L)\right]\left(\left[1 - \frac{\gamma_L^0}{2}\right] - [1 - \gamma_L^0]\right) \\
+ \gamma_L^2\left[(p_H - p_L)(\Delta x - \Delta m) - \beta_L\Delta e\right]\left(\left[1 - \frac{\gamma_L^2}{2}\right] - [1 - \gamma_L^2]\right) \\
= \left[\gamma_L^0(p_L - p_0) + \gamma_L^2(p_H - p_L)\right]\Delta m + \frac{(\gamma_L^0)^2}{2}\left[(p_L - p_0)(\Delta x - \Delta m) - (\bar{U} + \beta_L e_L)\right] + \\
\frac{(\gamma_L^2)^2}{2}\left[(p_H - p_L)(\Delta x - \Delta m) - \beta_L\Delta e\right]
\end{aligned}
$$

From the definition of Case 2, $(p_L - p_0)(\Delta x - \Delta m) > \bar{U} + \beta_H e_L > \bar{U} + \beta_L e_L$ and from Appendix B, $(p_H - p_L)(\Delta x - \Delta m) > \beta_H\Delta e > \beta_L\Delta e$. Therefore, $E[\pi|\,\text{req}] - E[\pi|\,\text{menu},\gamma_L^0 < 0.5] > 0$, so the firm prefers the request mechanism in Case 2 when its best menu of contracts induces the salesperson to exit if his territory is hard.

Next, consider $\gamma_L^2 < 0.5 < \gamma_L^0$:

$$
\begin{aligned}
E[\pi|\,\text{req}] - E[\pi|\,\text{menu}, \gamma_L^2 < 0.5 < \gamma_L^0] &= x_L + p_0\Delta x - \bar{U}_m + \gamma_L^0 \left[(p_L - p_0)\Delta x - (\bar{U} + \beta_H e_L)\right] + \\
&\quad \gamma_L^2 \left[(p_H - p_L)\Delta x - \beta_H \Delta e\right] + (\gamma_L^0 - \gamma_L^2)\frac{\gamma_L^0 + \gamma_L^2}{2}\left[(p_H - p_L)(\Delta x - \Delta m) - \beta_L \Delta e\right] + \\
&\quad (1-\gamma_L^0)\frac{1+\gamma_L^0}{2}\left[(p_H - p_0)(\Delta x - \Delta m) - (\bar{U} + \beta_L e_H)\right] - \\
&\quad \left(x_L + p_L\Delta x - \bar{U}_m - (\bar{U} + \beta_H e_L) + \frac{1}{2}\left[(p_H - p_L)(\Delta x - \Delta m) - \beta_L \Delta e\right]\right) \\
&= -(1-\gamma_L^0)\left[(p_L - p_0)\Delta x - (\bar{U} + \beta_H e_L)\right] - \frac{1}{2}\left[(p_H - p_L)(\Delta x - \Delta m) - \beta_L \Delta e\right] + \\
&\quad \gamma_L^2 \left[(p_H - p_L)\Delta x - \beta_H \Delta e\right] + (1-\gamma_L^2)\frac{1+\gamma_L^2}{2}\left[(p_H - p_L)(\Delta x - \Delta m) - \beta_L \Delta e\right] + \\
&\quad (1-\gamma_L^0)\frac{1+\gamma_L^0}{2}\left[(p_L - p_0)(\Delta x - \Delta m) - (\bar{U} + \beta_L e_L)\right] \\
&= (1-\gamma_L^0)\left(\left[\frac{1+\gamma_L^0}{2} - 1\right]\left[(p_L - p_0)(\Delta x - \Delta m) - (\bar{U} + \beta_L e_L)\right] + \Delta\beta e_L\right) + \\
&\quad \gamma_L^2\left(\left[1 - \frac{\gamma_L^2}{2}\right]\left[(p_H - p_L)(\Delta x - \Delta m) - \beta_L \Delta e\right] - \Delta\beta\Delta e\right) + \left[\gamma_L^2(p_H - p_L) - (1-\gamma_L^0)(p_L - p_0)\right]\Delta m \\
&= (1-\gamma_L^0)\left[(p_L - p_0)(\Delta x - \Delta m) - (\bar{U} + \beta_L e_L)\right]\left(\left[\frac{1+\gamma_L^0}{2} - 1\right] + \left[1 - \gamma_L^0\right]\right) + \\
&\quad \gamma_L^2\left[(p_H - p_L)(\Delta x - \Delta m) - \beta_L \Delta e\right]\left(\left[1 - \frac{\gamma_L^2}{2}\right] - \left[1 - \gamma_L^2\right]\right) + \\
&\quad \left[\gamma_L^2(p_H - p_L) - (1-\gamma_L^0)(p_L - p_0)\right]\Delta m \\
&= \left[\gamma_L^2(p_H - p_L) - (1-\gamma_L^0)(p_L - p_0)\right]\Delta m + \frac{1-\gamma_L^0}{2}\Delta\beta e_L + \frac{\gamma_L^2}{2}\left[(p_H - p_L)(\Delta x - \Delta m) - \beta_H \Delta e\right] \\
&= \left[\gamma_L^2(p_H - p_L) - (1-\gamma_L^0)(p_L - p_0)\right]\Delta m + \frac{1-\gamma_L^0}{2}\Delta\beta e_L + \\
&\quad \frac{1}{2}\left[(p_H - p_L)(\Delta x - \Delta m) - \beta_H \Delta e\right] - \frac{\gamma_L^2}{2}\Delta\beta\Delta e \\
&= \frac{1}{2}\left[(p_H - p_L)\Delta x - \beta_H \Delta e\right] + \frac{1-\gamma_L^0}{2}\Delta\beta e_L - \frac{\gamma_L^2}{2}\Delta\beta\Delta e - \left[\left(\frac{1}{2} - \gamma_L^2\right)(p_H - p_L) + (1-\gamma_L^0)(p_L - p_0)\right]\Delta m
\end{aligned}
$$

$\therefore$ the request mechanism is preferred if and only if

$$
\Delta m < \frac{(p_H - p_L)\Delta x - \beta_H \Delta e + (1-\gamma_L^0)\Delta\beta e_L - \gamma_L^2 \Delta\beta\Delta e}{2\left[\left(\frac{1}{2} - \gamma_L^2\right)(p_H - p_L) + (1-\gamma_L^0)(p_L - p_0)\right]}
$$

Therefore, when the firm's best menu of contracts induces (e_H, e_L), the request mechanism is preferred as long as Δm is not too large.

Finally, consider $\gamma_L^2 > 0.5$. Similar to above:

$$
\begin{aligned}
E[\pi|\,\text{req}]-E[\pi|\,\text{menu}, \gamma_L^2 > 0.5] &= x_L + p_0\Delta x - \bar{U}_m + \gamma_L^0 \left[(p_L - p_0)\Delta x - (\bar{U} + \beta_H e_L)\right] + \\
&\quad \gamma_L^2 \left[(p_H - p_L)\Delta x - \beta_H \Delta e\right] + (\gamma_L^0 - \gamma_L^2)\frac{\gamma_L^0 + \gamma_L^2}{2}\left[(p_H - p_L)(\Delta x - \Delta m) - \beta_L \Delta e\right] + \\
&\quad (1-\gamma_L^0)\frac{1+\gamma_L^0}{2}\left[(p_H - p_0)(\Delta x - \Delta m) - (\bar{U} + \beta_L e_H)\right] - \left[x_L + p_H \Delta x - (\bar{U}_m + \bar{U} + \beta_H e_H)\right] \\
&= -(1-\gamma_L^0)\left[(p_L - p_0)(\Delta x - \Delta m) - (\bar{U} + \beta_H e_L)\right] - (1-\gamma_L^2)\left[(p_H - p_L)(\Delta x - \Delta m) - \beta_H \Delta e\right] - \\
&\quad \left[(1-\gamma_L^0)(p_L - p_0) + (1-\gamma_L^2)(p_H - p_L)\right]\Delta m + (1-\gamma_L^2)\frac{1+\gamma_L^2}{2}\left[(p_H - p_L)(\Delta x - \Delta m) - \beta_L \Delta e\right] + \\
&\quad (1-\gamma_L^0)\frac{1+\gamma_L^0}{2}\left[(p_L - p_0)(\Delta x - \Delta m) - (\bar{U} + \beta_L e_L)\right] \\
&= (1-\gamma_L^0)\frac{\Delta\beta e_L}{2} + (1-\gamma_L^2)\frac{\Delta\beta\Delta e}{2} - \left[(1-\gamma_L^0)(p_L - p_0) + (1-\gamma_L^2)(p_H - p_L)\right]\Delta m
\end{aligned}
$$

$\therefore$ the request mechanism is preferred if and only if

$$
\Delta m < \frac{(1-\gamma_L^0)\Delta\beta e_L + (1-\gamma_L^2)\Delta\beta\Delta e}{2\left[(1-\gamma_L^0)(p_L - p_0) + (1-\gamma_L^2)(p_H - p_L)\right]}
$$

Thus, when the firm's best menu of contracts induces (e_H, e_H), the request mechanism is preferred as long as Δm is not too large.

Therefore, the request mechanism is preferred when the firm's best menu of contracts induces the salesperson to exit *or* when Δm is not too large. □

WEB APPENDIX G: PROOF OF PROPOSITION 4

First, consider the request mechanism. Following the approach from the main model, the extension is solved using backward induction.

Salesperson's contract acceptance & effort choice (Step 6)

Given a contract (s_L, s_H), the salesperson either exits or chooses effort e_i, $i \in \{L, H\}$, to satisfy:

$$s_L + [p(e_i) + p_m(e^s_m)]\Delta s - \beta e_i \geq \bar{U} \qquad (\text{IR}_i)$$

$$s_L + [p(e_i) + p_m(e^s_m)]\Delta s - \beta e_i \geq s_L + [p(e_j) + p_m(e^s_m)]\Delta s - \beta e_j \quad \forall j \in \{L, H\} \qquad (\text{IC}_i)$$

Thus, his approach is unchanged from the main model. If (IR_i) is not satisfied for either i, then he exits the firm. Otherwise, he accepts the contract and chooses $\begin{cases} e_L & \text{if } \beta\Delta e \geq (p_H - p_L)\Delta s \\ e_H & \text{if } \beta\Delta e \leq (p_H - p_L)\Delta s \end{cases}$

Manager's design of salesperson's contract & choice of support effort (Step 5)

Given $(e^{\beta_L}, e^{\beta_H})$ and her own request and support effort, the manager's expected utility is $E[U_m] = m_L + \left[\gamma_L p(e^{\beta_L}) + (1-\gamma_L)p(e^{\beta_H}) + p_m(e^s_m)\right]\Delta m - e^r_m - e^s_m$.

Therefore, she prefers $e^s_m = \begin{cases} 0 & \text{if } \Delta m < \frac{e^{s*}_m}{p_m(e^{s*}_m)} \\ e^{s*}_m & \text{if } \Delta m \geq \frac{e^{s*}_m}{p_m(e^{s*}_m)} \end{cases}$.

Recall **Lemma 1**: In the absence of constraints, there exists a contract to induce any combination of efforts $(e^{\beta_L}, e^{\beta_H})$ with $e^{\beta_L} \geq e^{\beta_H}$.

Proof: Following the same reasoning as in Web Appendix A:

The effort combination $(e^{\beta_L}, e^{\beta_H}) = (0,0)$ can be induced with an offer of $s_L = s_H = 0$.

$(e_L, 0)$ can be induced with $s_L = s_H = \bar{U} + \beta_L e_L$.

(e_L, e_L) can be induced with $s_L = s_H = \bar{U} + \beta_H e_L$.

$(e_H, 0)$ can be induced with $(s_L, s_H) = (\bar{U} + \beta_L e_H - [p_H + p_m(e^s_m)]\frac{\beta_L \Delta e}{p_H - p_L}, \bar{U} + \beta_L e_H + [1 - p_H - p_m(e^s_m)]\frac{\beta_L \Delta e}{p_H - p_L})$.

(e_H, e_L) can be induced with the same $\Delta s = \frac{\beta_L \Delta e}{p_H - p_L}$ and an arbitrarily high s_L to ensure that (IR_L) is satisfied in both territory types.

Finally, (e_H, e_L) can be induced with an arbitrarily high Δs and any non-negative s_L. □

As in the main model, the manager benefits from the highest possible effort in each territory type, so her preferences among combinations $(e^{\beta_L}, e^{\beta_H})$ are unchanged.

Manager's choice of request effort (Step 4)

From Step 5, the manager's choice of e_m^s depends only on Δm and the value of e_m^{s*}, so it does *not* depend on whether she chooses to make a request. Therefore, given baseline constraints that allow her to induce $(e^{\beta_L}, e^{\beta_H})$ with the opportunity to exert effort e_m^{r*} to request relaxed constraints allowing $(e^{\beta_L\prime}, e^{\beta_H\prime})$, the manager makes the request if and only if she prefers $(e^{\beta_L\prime}, e^{\beta_H\prime})$ over $(e^{\beta_L}, e^{\beta_H})$ and:

$$m_L + \left[\gamma_L p(e^{\beta_L\prime}) + (1-\gamma_L)p(e^{\beta_H\prime}) + p_m(e_m^s)\right]\Delta m - e_m^{r*} - e_m^s$$
$$> m_L + \left[\gamma_L p(e^{\beta_L}) + (1-\gamma_L)p(e^{\beta_H}) + p_m(e_m^s)\right]\Delta m - e_m^s$$

$$\iff \gamma_L\left(\left[p(e^{\beta_H\prime}) - p(e^{\beta_H})\right] - \left[p(e^{\beta_L\prime}) - p(e^{\beta_L})\right]\right)\Delta m < \left[p(e^{\beta_H\prime}) - p(e^{\beta_H})\right]\Delta m - e_m^{r*}$$

This is the same condition as in the main model, so the remainder of this step is unchanged. Therefore, the manager requests relaxed constraints when γ_L is sufficiently low and Equation 2 holds when $e^{\beta_L} = e^{\beta_L\prime}$.

Firm's choice of constraints & request requirements (Step 3)

Recall **Lemma 2**: A hard cap on the total payout $(\widehat{s_H})$ and a hard cap on either the salary $(\widehat{s_L})$ or the bonus $(\widehat{\Delta s})$ is sufficient to implement an efficient contract for any combination of efforts $(e^{\beta_L}, e^{\beta_H})$ with $e^{\beta_L} \geq e^{\beta_H}$.

Proof: The firm's best contract to induce non-zero efforts $(e^{\beta_L}, e^{\beta_H})$ solves the following:

$$\max_{(s_L, s_H)} E[\pi] = x_L - m_L - s_L + \left(\gamma_L^F p[e^{\beta_L}] + [1-\gamma_L^F]p[e^{\beta_H}] + p_m[e_m^s]\right)(\Delta x - \Delta m - \Delta s)$$

$$\text{s.t. } s_L + [p(e^{\beta_L}) + p_m(e_m^s)]\Delta s - \beta_L e^{\beta_L} \geq \bar{U} \qquad (\text{IR}^{\beta_L})$$

$$s_L + [p(e^{\beta_H}) + p_m(e_m^s)]\Delta s - \beta_H e^{\beta_H} \geq \bar{U} \qquad (\text{IR}^{\beta_H})$$

$$s_L + [p(e^{\beta_L}) + p_m(e_m^s)]\Delta s - \beta_L e^{\beta_L} \geq s_L + [p(e^*) + p_m(e_m^s)]\Delta s - \beta_L e^* \quad \forall e^* \in \{e_L, e_H\} \qquad (\text{IC}^{\beta_L})$$

$$s_L + [p(e^{\beta_H}) + p_m(e_m^s)]\Delta s - \beta_H e^{\beta_H} \geq s_L + [p(e^*) + p_m(e_m^s)]\Delta s - \beta_H e^* \quad \forall e^* \in \{e_L, e_H\} \qquad (\text{IC}^{\beta_H})$$

$$s_L \geq 0 \qquad (\text{LL})$$

where e_m^s is the manager's best response to (m_L, m_H).

First, consider the identical problem, but with the (LL) constraint removed.

At least one of the (IR) constraints must bind. (Otherwise, the firm could decrease s_L while holding Δs constant, increasing the objective function without violating any constraints.) As shown for the main model, (IR$^{\beta_L}$) holds with slack, so (IR$^{\beta_H}$) must bind in any efficient contract.

$\therefore\ s_L = \bar{U} + \beta_H e^{\beta_H} - [p(e^{\beta_H}) + p_m(e_m^s)]\Delta s$ and $s_H = \bar{U} + \beta_H e^{\beta_H} + [1 - p(e^{\beta_H}) - p_m(e_m^s)]\Delta s$

Suppose $(e^{\beta_L}, e^{\beta_H}) = \boldsymbol{(e_H, e_H)}$

By (IC$^{\beta_H}$), $\Delta s \geq \frac{\beta_H \Delta e}{p_H - p_L}$

$\therefore\ s_H \geq \bar{U} + \beta_H e_H + [1 - p_H - p_m(e_m^s)]\frac{\beta_H \Delta e}{p_H - p_L}$

Thus, while the manager will induce (e_H, e_H) efficiently with *no* constraints, the lowest total payout cap that *allows* (e_H, e_H) is $\widehat{s_H}^{(e_H, e_H)} = \bar{U} + \beta_H e_H + [1 - p_H - p_m(e_m^s)]\frac{\beta_H \Delta e}{p_H - p_L}$

Now, suppose $(e^{\beta_L}, e^{\beta_H}) = \boldsymbol{(e_H, e_L)}$:

(IR$^{\beta_H}$) binds $\implies s_L = \bar{U} + \beta_H e_L - [p_L + p_m(e_m^s)]\Delta s$

From (IC$^{\beta_L}$), $\Delta s \geq \frac{\beta_L \Delta e}{p_H - p_L}$

The firm's objective function can be simplified to:

$$\max_{(s_L, s_H)} -\left(s_L + \left[\gamma_L^F p_H + (1 - \gamma_L^F)p_L + p_m(e_m^s)\right]\Delta s\right)$$

Substituting for s_L gives:

$$\max_{(s_L, s_H)} -\left(\bar{U} + \beta_H e_L + \gamma_L^F [p_H - p_L]\Delta s\right)$$

$$\implies \max_{(s_L, s_H)} -\Delta s$$

Therefore, under the efficient contract for (e_H, e_L), $\Delta s = \frac{\beta_L \Delta e}{p_H - p_L}$

$\therefore\ s_H = \bar{U} + \beta_H e_L + (1 - [p_L + p_m(e_m^s)])\frac{\beta_L \Delta e}{p_H - p_L}$

Suppose, then, that the firm sets $\widehat{s_H}^{(e_H, e_L)} = \bar{U} + \beta_H e_L + (1 - [p_L + p_m(e_m^s)])\frac{\beta_L \Delta e}{p_H - p_L}$

$$
\begin{aligned}
\widehat{s_H}^{(e_H,e_H)} - \widehat{s_H}^{(e_H,e_L)} &= \bar{U} + \beta_H e_H + [1 - p_H - p_m(e_m^s)]\frac{\beta_H \Delta e}{p_H - p_L} - \left(\bar{U} + \beta_H e_L + (1 - [p_L + p_m(e_m^s)])\frac{\beta_L \Delta e}{p_H - p_L}\right) \\
&= \frac{\Delta e}{p_H - p_L}[\beta_H(p_H - p_L) + [1 - p_m(e_m^s)]\Delta\beta - \beta_H p_H + \beta_L p_L] \\
&= [1 - p_L - p_m(e_m^s)]\frac{\Delta\beta\Delta e}{p_H - p_L} \qquad \text{(because } p_H + p_m(e_m^{s*}) < 1\text{)} \\
&> 0 \\
\therefore \widehat{s_H}^{(e_H,e_L)} &< \widehat{s_H}^{(e_H,e_H)}
\end{aligned}
$$

Therefore, $\widehat{s_H}^{(e_H,e_L)}$ does not allow (e_H, e_H), so the manager will choose to implement $(e^{\beta_L}, e^{\beta_H}) = (e_H, e_L)$ efficiently.

As shown for the main model, the firm can implement $(e^{\beta_L}, e^{\beta_H}) = \mathbf{(0, 0)}$ or $\boldsymbol{(e_L, 0)}$ by setting $\widehat{s_H}^{(0,0)} = 0$ or $\widehat{s_H}^{(e_L,0)} = \bar{U} + \beta_L e_L$, respectively.

That leaves $\boldsymbol{(e_H, 0)}$ and $\boldsymbol{(e_L, e_L)}$:

Under an efficient contract for $(e_H, 0)$, the salesperson is expected to exit if the territory is hard, so the firm's efficient contract matches that of a fully informed firm in an easy territory. Therefore, from the standard principal-agent model, (IR^{β_L}) must bind, so $s_L = \bar{U} + \beta_L e_H - [p_H + p_m(e_m^s)]\Delta s$

By (IC^{β_L}), $\Delta s \geq \frac{\beta_L \Delta e}{p_H - p_L}$.

$\therefore s_H \geq \bar{U} + \beta_L e_H + [1 - p_H - p_m(e_m^s)]\frac{\beta_L \Delta e}{p_H - p_L}$

Thus, the minimum total payout cap that could allow $(e_H, 0)$ to be implemented efficiently is $\widehat{s_H}^{(e_H,0)} = \bar{U} + \beta_L e_H + [1 - p_H - p_m(e_m^s)]\frac{\beta_L \Delta e}{p_H - p_L}$

Given that cap, if the manager chooses to induce $e^{\beta_L} = e_H$, by offering $(s_L, s_H) = (\bar{U} + \beta_L e_H - [p_H + p_m(e_m^s)]\frac{\beta_L \Delta e}{p_H - p_L}, \widehat{s_H}^{(e_H,0)})$, it is straightforward to show that (IR^{β_H}) cannot be satisfied for e_L or e_H, so the salesperson does, indeed, exit if the territory is hard. In other words, $\widehat{s_H}^{(e_H,0)}$ allows $(e_H, 0)$, but not (e_H, e) for $e > 0$.

From above, under the efficient contract for (e_L, e_L), (IR^{β_H}) must bind, so $s_L = \bar{U} + \beta_H e_L - [p_L + p_m(e_m^s)]\Delta s$.

Since there is no minimum constraint on Δs to induce e_L, the minimum total payout cap that

could allow (e_L, e_L) is simply $\widehat{s_H}^{(e_L,e_L)} = \bar{U} + \beta_H e_L$.

Case 1: Suppose $\widehat{s_H}^{(e_L,e_L)} < \widehat{s_H}^{(e_H,0)}$:

By the same reasoning as in the main model, $\widehat{s_H}^{(e_L,e_L)}$ is sufficient to implement (e_L, e_L) efficiently and $(e_H, 0)$ can be implemented by imposing $\widehat{s_L}^{(e_H,0)} = \bar{U} + \beta_L e_H - [p_H + p_m(e_m^s)]\frac{\beta_L \Delta e}{p_H - p_L}$, in addition to $\widehat{s_H}^{(e_H,0)}$.

Case 2: Suppose $\widehat{s_H}^{(e_H,0)} < \widehat{s_H}^{(e_L,e_L)}$:

Again, following the reasoning from the main model, $\widehat{s_H}^{(e_H,0)}$ is sufficient to implement $(e_H, 0)$ and (e_L, e_L) can be implemented by imposing $\widehat{\Delta s}^{(e_L,e_L)} = 0$ in addition to $\widehat{s_H}^{(e_L,e_L)}$.

Claim: The solutions above remain feasible when the (LL) constraint is applied, implying that they are also solutions to this more-constrained problem.

Proof: Under $\widehat{s_H}^{(e_H,e_H)} = \bar{U} + \beta_H e_H + [1 - p_H - p_m(e_m^s)]\frac{\beta_H \Delta e}{p_H - p_L}$, the manager can induce (e_H, e_H) by offering $(s_L, s_H) = (\bar{U} + \beta_H e_H - [p_H + p_m(e_m^s)]\frac{\beta_H \Delta e}{p_H - p_L}, \widehat{s_H}^{(e_H,e_H)})$. By assumption, $\bar{U} \geq \frac{\beta_H}{p_H - p_L}(p_L e_H - p_H e_L + p_m(e_m^{s*})\Delta e) \geq 0$, so $s_L \geq [p_m(e_m^{s*}) - p_m(e_m^s)]\frac{\beta_H \Delta e}{p_H - p_L} \geq 0$.

Under $\widehat{s_H}^{(e_H,e_L)} = \bar{U} + \beta_H e_L + (1 - [p_L + p_m(e_m^s)])\frac{\beta_L \Delta e}{p_H - p_L}$, the manager can induce (e_H, e_L) by offering $(s_L, s_H) = (\bar{U} + \beta_H e_L - [p_L + p_m(e_m^s)]\frac{\beta_L \Delta e}{p_H - p_L}, \widehat{s_H}^{(e_H,e_L)})$. $\therefore$ $s_L \geq [p_L + p_m(e_m^{s*})]\frac{\Delta\beta \Delta e}{p_H - p_L} > 0$.

Under $(\widehat{s_L}^{(e_H,0)}, \widehat{s_H}^{(e_H,0)}) = (\bar{U} + \beta_L e_H - [p_H + p_m(e_m^s)]\frac{\beta_L \Delta e}{p_H - p_L}, \bar{U} + \beta_L e_H + [1 - p_H - p_m(e_m^s)]\frac{\beta_L \Delta e}{p_H - p_L})$, the manager can induce $(e_H, 0)$ by offering $(s_L, s_H) = (\widehat{s_L}^{(e_H,0)}, \widehat{s_H}^{(e_H,0)})$. Here, $s_L \geq \frac{\Delta\beta}{p_H - p_L}(p_L e_H - p_H e_L + p_m(e_m^{s*})\Delta e) \geq 0$.

Under $(\widehat{\Delta s}^{(e_L,e_L)}, \widehat{s_H}^{(e_L,e_L)}) = (0, \bar{U} + \beta_H e_L)$, the manager can induce (e_L, e_L) by offering $(s_L, s_H) = (\widehat{s_H}^{(e_L,e_L)}, \widehat{s_H}^{(e_L,e_L)})$. $\therefore$ $s_L > 0$.

Similarly, under $\widehat{s_H}^{(e_L,0)} = \bar{U} + \beta_L e_L$, the manager can induce $(e_L, 0)$ by offering $(s_L, s_H) = (\widehat{s_H}^{(e_L,0)}, \widehat{s_H}^{(e_L,0)})$, so $s_L > 0$.

$\therefore$ (LL) does not prevent the firm from implementing any of the above solutions. □

Therefore, a hard cap on the salesperson's total payout combined with a hard cap on either his salary or his bonus is sufficient to implement an efficient contract for any combination of efforts $(e^{\beta_L}, e^{\beta_H})$ with $e^{\beta_L} \geq e^{\beta_H}$. □

Next, recall **Lemma 3**: An efficient contract to induce efforts $(e^{\beta_L}, e^{\beta_H})$ has the following

properties: When the territory is hard, the salesperson receives no surplus; when the territory is easy, the salesperson receives an expected utility surplus of $\Delta\beta e^{\beta_H}$.

Proof: **Suppose $e^{\beta_H} = 0$**. Again, the firm can act as though it knows that the territory is easy, so the efficient contract matches the full-information solution for an easy territory. (IR$^{\beta_L}$) binds under the full-information solution, so the salesperson's surplus is 0 ($= \Delta\beta e^{\beta_H}$) if the territory is easy. It is straightforward to show that the salesperson will exit (and receive no surplus) if the territory is hard. Therefore, the Lemma holds when $e^{\beta_H} = 0$.

Next, **suppose $e^{\beta_L} = e^{\beta_H} > 0$**:

From the proof of Lemma 2 above, (IR$^{\beta_H}$) binds in any efficient contract inducing non-zero $(e^{\beta_L}, e^{\beta_H})$, so the salesperson receives no surplus when the territory is hard. Therefore, $s_L + [p(e^{\beta_H}) + p_m(e_m^s)]\Delta s - \beta_H e^{\beta_H} = \bar{U}$ and $s_L + [p(e^{\beta_L}) + p_m(e_m^s)]\Delta s - \beta_L e^{\beta_L} = \bar{U} + \Delta\beta e^{\beta_H}$ (because $e^{\beta_L} = e^{\beta_H}$), so he receives a surplus of $\Delta\beta e^{\beta_H}$ when it is easy. Therefore, the Lemma holds when $e^{\beta_L} = e^{\beta_H} > 0$.

Lastly, **suppose $(e^{\beta_L}, e^{\beta_H}) = (e_H, e_L)$**:

From above, the efficient contract to induce (e_H, e_L) is $(s_L, s_H) = (\bar{U} + \beta_H e_L - [p_L + p_m(e_m^s)]\frac{\beta_L \Delta e}{p_H - p_L}, \bar{U} + \beta_H e_L + [1 - p_L - p_m(e_m^s)]\frac{\beta_L \Delta e}{p_H - p_L})$. Again, (IR$^{\beta_H}$) binds, so the salesperson receives no surplus when the territory is hard. Furthermore, $s_L + [p_H + p_m(e_m^s)]\Delta s - \beta_L e_H = \bar{U} + \Delta\beta e_L$, so the salesperson receives a surplus of $\Delta\beta e^{\beta_H}$ when the territory is easy.

Thus, under an efficient contract to induce $(e^{\beta_L}, e^{\beta_H})$ with $e^{\beta_L} \geq e^{\beta_H}$, the salesperson receives no surplus when the territory is hard and a surplus of $\Delta\beta e^{\beta_H}$ when it is easy. □

Now, recall **Proposition 1**: The firm's best possible contract has the following properties: a) Effort induced in a hard territory (weakly) decreases in the belief (γ_L) that the territory is easy; and b) Effort induced in an easy territory is independent of γ_L. In this setting, that effort is always e_H.

Proof: By Lemma 3, under an efficient contract that induces positive $(e^{\beta_L}, e^{\beta_H})$: $s_L + [p(e^{\beta_H}) + p_m(e_m^s)]\Delta s = \bar{U} + \beta_H e^{\beta_H}$ and $s_L + [p(e^{\beta_L}) + p_m(e_m^s)]\Delta s = \bar{U} + \beta_L e^{\beta_L} + \Delta\beta e^{\beta_H}$.

The manager's contract is fixed at this point, so her support effort (e_m^s) can be anticipated, as it depends only on Δm and e_m^{s*}. Therefore, given belief γ_L, the firm's expected profit under each

feasible $(e^{\beta_L}, e^{\beta_H})$ is:

$$E[\pi(e_L,0)] = x_L - m_L + \left[p_0 + p_m(e_m^s) + \gamma_L(p_L - p_0)\right](\Delta x - \Delta m) - \gamma_L(\bar{U} + \beta_L e_L)$$
$$E[\pi(e_L,e_L)] = x_L - m_L + \left[p_L + p_m(e_m^s)\right](\Delta x - \Delta m) - (\bar{U} + \beta_H e_L)$$
$$E[\pi(e_H,0)] = x_L - m_L + \left[p_0 + p_m(e_m^s) + \gamma_L(p_H - p_0)\right](\Delta x - \Delta m) - \gamma_L(\bar{U} + \beta_L e_H)$$
$$E[\pi(e_H,e_L)] = x_L - m_L + \left[p_L + p_m(e_m^s) + \gamma_L(p_H - p_L)\right](\Delta x - \Delta m) - (\bar{U} + \beta_H e_L + \gamma_L \beta_L \Delta e)$$
$$E[\pi(e_H,e_H)] = x_L - m_L + \left[p_H + p_m(e_m^s)\right](\Delta x - \Delta m) - (\bar{U} + \beta_H e_H)$$

Claim: If the territory is easy, the effort induced is independent of γ_L.

Proof: Comparing the above gives:

$$E[\pi(e_L,0)] - E[\pi(e_H,0)] = \gamma_L[\beta_L \Delta e - (p_H - p_L)(\Delta x - \Delta m)]$$
$$E[\pi(e_L,e_L)] - E[\pi(e_H,e_L)] = \gamma_L[\beta_L \Delta e - (p_H - p_L)(\Delta x - \Delta m)]$$
$$E[\pi(e_L,e_L)] - E[\pi(e_H,e_H)] = \beta_H \Delta e - (p_H - p_L)(\Delta x - \Delta m)$$

It is clear that a contract inducing $e^{\beta_L} = e_L$ can be best if and only if $(p_H - p_L)(\Delta x - \Delta m) < \beta_L \Delta e$. Otherwise, the firm's best contract must induce either $(e_H, 0)$, (e_H, e_L), or (e_H, e_H).

Therefore, the effort induced if the territory is easy (e^{β_L}) is independent of γ_L. □

Claim: $(p_H - p_L)(\Delta x - \Delta m) > \beta_H \Delta e$

Proof: By assumption, the firm prefers to employ the salesperson (over letting him exit) even when the territory is known to be hard. This implies that at least one of the following must hold:

$$(p_L - p_0)(\Delta x - \Delta m) > \bar{U} + \beta_H e_L \quad \text{(W3)}$$
$$(p_H - p_0)(\Delta x - \Delta m) > \bar{U} + \beta_H e_H \quad \text{(W4)}$$

By assumption, $\bar{U} \geq \frac{\beta_H}{p_H - p_L}(p_L e_H - p_H e_L + p_m(e_m^{s*})\Delta e)$. Substituting this into Equation W3

gives

$$(p_L - p_0)(\Delta x - \Delta m) > \frac{\beta_H}{p_H - p_L}(p_L e_H - p_H e_L + p_m(e_m^{s*})\Delta e) + \beta_H e_L$$
$$\therefore (p_L - p_0)(\Delta x - \Delta m) > \frac{\beta_H \Delta e}{p_H - p_L}[p_L + p_m(e_m^{s*})]$$
$$\therefore (p_H - p_L)(\Delta x - \Delta m) > \beta_H \Delta e$$

Similarly, Equation W4 gives

$$(p_H - p_0)(\Delta x - \Delta m) > \frac{\beta_H \Delta e}{p_H - p_L}[p_H + p_m(e_m^{s*})]$$
$$\therefore (p_H - p_L)(\Delta x - \Delta m) > \beta_H \Delta e$$

Since at least one of these must hold, $\therefore (p_H - p_L)(\Delta x - \Delta m) > \beta_H \Delta e$. □

From above, this implies that the firm's best contract must induce $(e_H, 0)$, (e_H, e_L), or (e_H, e_H).

Among those three options:

$E[\pi(e_H,0)] - E[\pi(e_H,e_L)] = \gamma_L[(p_L - p_0)(\Delta x - \Delta m) - (\bar{U} + \beta_L e_L)] - [(p_L - p_0)(\Delta x - \Delta m) - (\bar{U} + \beta_H e_L)]$

$\therefore$ if $(p_L - p_0)(\Delta x - \Delta m) < \bar{U} + \beta_H e_L$, then $E[\pi(e_H,0)] - E[\pi(e_H,e_L)] > 0$, so $(e_H, 0)$ is preferred over (e_H, e_L) for any γ_L.

Otherwise, $E[\pi(e_H,0)] > E[\pi(e_H,e_L)] \iff \gamma_L > \frac{(p_L - p_0)(\Delta x - \Delta m) - (\bar{U} + \beta_H e_L)}{(p_L - p_0)(\Delta x - \Delta m) - (\bar{U} + \beta_L e_L)} = \gamma_L^0$

$E[\pi(e_H,0)] - E[\pi(e_H,e_H)] = \gamma_L[(p_H - p_0)(\Delta x - \Delta m) - (\bar{U} + \beta_L e_H)] - [(p_H - p_0)(\Delta x - \Delta m) - (\bar{U} + \beta_H e_H)]$

$\therefore E[\pi(e_H,0)] > E[\pi(e_H,e_H)] \iff \gamma_L > \frac{(p_H - p_0)(\Delta x - \Delta m) - (\bar{U} + \beta_H e_H)}{(p_H - p_0)(\Delta x - \Delta m) - (\bar{U} + \beta_L e_H)} = \gamma_L^1$

$E[\pi(e_H,e_L)] - E[\pi(e_H,e_H)] = \gamma_L[(p_H - p_L)(\Delta x - \Delta m) - \beta_L \Delta e] - [(p_H - p_L)(\Delta x - \Delta m) - \beta_H \Delta e]$

$\therefore E[\pi(e_H,e_L)] > E[\pi(e_H,e_H)] \iff \gamma_L > \frac{(p_H - p_L)(\Delta x - \Delta m) - \beta_H \Delta e}{(p_H - p_L)(\Delta x - \Delta m) - \beta_L \Delta e} = \gamma_L^2$

$\therefore$ In each comparison, the firm's preferred effort if the territory is hard weakly decreases in γ_L. □

Next, recall **Proposition 2**: The request mechanism is always feasible. In other words, the firm can always define baseline and relaxed constraints and corresponding request requirements such that the manager will choose to request constraints that are best for the firm, given her own beliefs.

Proof: From Proposition 1, the firm can narrow consideration to constraints resulting in $(e^{\beta_L}, e^{\beta_H}) = (e_H, 0)$, (e_H, e_L), or (e_H, e_H). As in the main model, the manager prefers to in-

duce the highest possible e^{β_H}, so the firm sets baseline constraints that result in $e^{\beta_H} = 0$ and requirements for the manager to request relaxed constraints that allow for $e^{\beta_H} = e_L$ and/or e_H.

From above, as in the main model, it is clear that the firm can rule out (e_H, e_L) for any value of γ_L when either: a) $(p_L - p_0)(\Delta x - \Delta m) < \bar{U} + \beta_H e_L$; or b) $\gamma_L^0 < \gamma_L^2$, so this is considered as a separate case. The analysis of these cases is unchanged from the main model, so it is briefly summarized here.

Case 1: $(p_L - p_0)(\Delta x - \Delta m) < \bar{U} + \beta_H e_L$ OR $\gamma_L^0 < \gamma_L^2$

The firm sets baseline constraints that result in $(e^{\beta_L}, e^{\beta_H}) = (e_H, 0)$ and requirements $e_m^{r*} = (1 - \gamma_L^1)(p_H - p_0)\Delta m$ for the manager to request constraints allowing (e_H, e_H). From Equation 2, the manager will make the request if and only if $\gamma_L < \frac{(p_H - p_0)\Delta m - e_m^{r*}}{(p_H - p_0)\Delta m} = \gamma_L^1$, as desired by the firm.

Thus, the mechanism is feasible when $(p_L - p_0)(\Delta x - \Delta m) < \bar{U} + \beta_H e_L$ or $\gamma_L^0 < \gamma_L^2$.

Case 2: Otherwise $\big((p_L - p_0)(\Delta x - \Delta m) > \bar{U} + \beta_H e_L$ AND $\gamma_L^0 > \gamma_L^2\big)$

By Proposition 1 and the profit comparisons above, the firm prefers
$$\begin{cases} (e_H, e_H) & \text{if } \gamma_L < \gamma_L^2 \\ (e_H, e_L) & \text{if } \gamma_L^2 < \gamma_L < \gamma_L^0 \\ (e_H, 0) & \text{if } \gamma_L^0 < \gamma_L \end{cases}$$

The firm sets baseline constraints that result in $(e^{\beta_L}, e^{\beta_H}) = (e_H, 0)$ and sequential requirements $e_m^{rL} = (1 - \gamma_L^0)(p_L - p_0)\Delta m$ and $e_m^{rH} = (1 - \gamma_L^2)(p_H - p_L)\Delta m$ for the manager to request constraints allowing (e_H, e_L) and (e_H, e_H), respectively.

From Equation 2, the manager makes the first request if and only if $\gamma_L < \frac{(p_L - p_0)\Delta m - e_m^{rL}}{(p_L - p_0)\Delta m} = \gamma_L^0$ and the second request if and only if $\gamma_L < \frac{(p_H - p_L)\Delta m - e_m^{rH}}{(p_H - p_L)\Delta m} = \gamma_L^2$, as the firm prefers.

Once again, three questions remain: 1) Does the sequential nature of these requests ever prevent the first request from being made when the second is desired (by the firm)? 2) Does the manager ever make a suboptimal first request in order to gain the opportunity to make the second one? 3) Why is it necessary for the requests to be sequential? The answers to these are unchanged from the main model and are found in Web Appendix D. □

From this proof, it is clear that the constraints and request requirements chosen by the firm, summarized in Table 2, are unchanged from the main model.

Manager's contract acceptance (Step 2)

As in the main model, when deciding whether to accept the firm's contract offer, the manager can anticipate the request mechanism and her own request effort. In this extension, she can also anticipate her choice of support effort in Step 5.

When $(e^{\beta_L}, e^{\beta_H}) = (e_H, e_L)$ can be ruled out (i.e., Case 1), the manager's expected utility if she makes a request is

$$
\begin{aligned}
E[U_m|\gamma_L < \gamma_L^1] &= m_L + [p_H + p_m(e_m^s)]\Delta m - e_m^{r*} - e_m^s \\
&= m_L + [p_H + p_m(e_m^s)]\Delta m - (1-\gamma_L^1)(p_H - p_0)\Delta m - e_m^s \\
\therefore E[U_m|\gamma_L < \gamma_L^1] &= m_L + \left(p_0 + \gamma_L^1[p_H - p_0] + p_m(e_m^s)\right)\Delta m - e_m^s
\end{aligned}
$$

When she does not make a request, the baseline constraints allow her to induce $(e_H, 0)$, so

$$E[U_m|\gamma_L > \gamma_L^1] = m_L + \left(p_0 + \gamma_L[p_H - p_0] + p_m(e_m^s)\right)\Delta m - e_m^s$$

In the remaining case (when (e_H, e_L) is not ruled out), a similar analysis gives

$$
\begin{aligned}
E[U_m|\gamma_L < \gamma_L^2] &= m_L + \left(p_0 + \gamma_L^0[p_L - p_0] + \gamma_L^2[p_H - p_L] + p_m(e_m^s)\right)\Delta m - e_m^s \\
E[U_m|\gamma_L \in (\gamma_L^2, \gamma_L^0)] &= m_L + \left(p_0 + \gamma_L^0[p_L - p_0] + \gamma_L[p_H - p_L] + p_m(e_m^s)\right)\Delta m - e_m^s \\
E[U_m|\gamma_L > \gamma_L^0] &= m_L + \left(p_0 + \gamma_L[p_H - p_0] + p_m(e_m^s)\right)\Delta m - e_m^s
\end{aligned}
$$

In either case, then, the manager's expected utility is weakly increasing in her belief, γ_L. Thus, if she accepts a given contract when $\gamma_L \to 0$, then she accepts it for *any* γ_L.

Firm's design of manager's contract (Step 1)

When designing the manager's contract, the firm does not observe her belief about the territory type. Therefore, in order to implement the request mechanism, the firm must ensure that the manager accepts her contract for *any* value of γ_L. Furthermore, by assumption, the firm prefers that the manager not only accepts, but exerts support effort e_m^{s*} (rather than 0). From Step 5, then, the firm must set $\Delta m \geq \frac{e_m^{s*}}{p_m(e_m^{s*})}$.

Case 1: $(p_L - p_0)(\Delta x - \Delta m) < \bar{U} + \beta_H e_L$ OR $\gamma_L^0 < \gamma_L^2$

The firm chooses the manager's contract by solving the following:

$$\max_{m_L,\Delta m} E[\pi] = \int_0^{\gamma_L^1} E[\pi(e_H, e_H)] f(\gamma_L) \mathrm{d}\gamma_L + \int_{\gamma_L^1}^1 E[\pi(e_H, 0)] f(\gamma_L) \mathrm{d}\gamma_L$$

$$\text{s.t. } m_L + \left(p_0 + \gamma_L^1[p_H - p_0] + p_m(e_m^{s*})\right) \Delta m - e_m^{s*} \geq \bar{U}_m \qquad (\text{IR}_\text{m})$$

$$\Delta m \geq \frac{e_m^{s*}}{p_m(e_m^{s*})} \qquad (\text{IC}_\text{m})$$

$$m_L \geq 0 \qquad (\text{LL}_\text{m})$$

where, from Lemma 3:

$$\begin{aligned} E[\pi(e_H, e^{\beta_H})] = x_L - m_L + \gamma_L \left(\left[p_H + p_m(e_m^{s*}) \right] (\Delta x - \Delta m) - (\bar{U} + \beta_L e_H + \Delta\beta e^{\beta_H}) \right) + \\ (1 - \gamma_L) \left(\left[p(e^{\beta_H}) + p_m(e_m^{s*}) \right] (\Delta x - \Delta m) - \mathbb{1}_{e^{\beta_H} > 0} (\bar{U} + \beta_H e^{\beta_H}) \right) \end{aligned} \qquad (\text{W5})$$

As in the main model, $f(\gamma_L) = 1 \; \forall \; \gamma_L \in (0, 1)$. Therefore, the objective function is:

$$\begin{aligned} E[\pi] = &\int_0^{\gamma_L^1} \left[x_L - m_L + \left[p_H + p_m(e_m^{s*}) \right] (\Delta x - \Delta m) - (\bar{U} + \beta_H e_H) \right] \mathrm{d}\gamma_L + \\ &\int_{\gamma_L^1}^1 \left[x_L - m_L + \left[p_0 + p_m(e_m^{s*}) \right] (\Delta x - \Delta m) + \gamma_L \left([p_H - p_0] (\Delta x - \Delta m) - [\bar{U} + \beta_L e_H] \right) \right] \mathrm{d}\gamma_L \\ &= \gamma_L^1 \left[x_L - m_L + \left[p_H + p_m(e_m^{s*}) \right] (\Delta x - \Delta m) - (\bar{U} + \beta_H e_H) \right] + \\ &(1 - \gamma_L^1) \left[x_L - m_L + \left[p_0 + p_m(e_m^{s*}) \right] (\Delta x - \Delta m) + \frac{1 + \gamma_L^1}{2} \left([p_H - p_0] (\Delta x - \Delta m) - [\bar{U} + \beta_L e_H] \right) \right] \\ &= x_L - m_L + \left[p_0 + p_m(e_m^{s*}) \right] (\Delta x - \Delta m) + \gamma_L^1 \left[(p_H - p_0)(\Delta x - \Delta m) - (\bar{U} + \beta_H e_H) \right] + \\ &\quad (1 - \gamma_L^1) \frac{1 + \gamma_L^1}{2} \left([p_H - p_0] [\Delta x - \Delta m] - [\bar{U} + \beta_L e_H] \right) \end{aligned}$$

Removing fixed values, the firm's problem becomes

$$\max_{m_L,\Delta m} -\left(m_L + \left[p_0 + \gamma_L^1[p_H - p_0] + (1-\gamma_L^1)\frac{1+\gamma_L^1}{2}[p_H - p_0] + p_m(e_m^{s*})\right]\Delta m\right)$$

$$\text{s.t. } m_L + \left(p_0 + \gamma_L^1[p_H - p_0] + p_m(e_m^{s*})\right)\Delta m - e_m^{s*} \geq \bar{U}_m \qquad (\text{IR}_\text{m})$$

$$\Delta m \geq \frac{e_m^{s*}}{p_m(e_m^{s*})} \qquad (\text{IC}_\text{m})$$

$$m_L \geq 0 \qquad (\text{LL}_\text{m})$$

Again, consider first the identical problem, but with the (LL_m) constraint removed. Then (IR_m) must bind, because otherwise the firm can decrease m_L and increase the objective function without violating any constraints.

$\therefore m_L = \bar{U}_m + e_m^{s*} - \left(p_0 + \gamma_L^1[p_H - p_0] + p_m(e_m^{s*})\right)\Delta m$

Then the objective function is

$$\max_{\Delta m} -\left(\bar{U}_m + e_m^{s*} + (1-\gamma_L^1)\frac{1+\gamma_L^1}{2}[p_H - p_0]\Delta m\right)$$

$$\iff \min_{\Delta m}\left(1 - \left[\gamma_L^1\right]^2\right)\Delta m$$

Since $\gamma_L^1 = 1 - \frac{\Delta\beta e_H}{(p_H - p_0)(\Delta x - \Delta m) - (\bar{U} + \beta_L e_H)}$ is positive and decreasing in Δm, $\left(1 - \left[\gamma_L^1\right]^2\right)\Delta m$ is increasing in Δm. Therefore, the firm prefers to minimize Δm. This is driven by the fact that when $\gamma_L > \gamma_L^1$, the manager earns rent that is increasing in Δm.

$$\therefore \Delta m^* = \frac{e_m^{s*}}{p_m(e_m^{s*})}$$

$$\therefore m_L^* = \bar{U}_m + e_m^{s*} - \left(p_0 + \gamma_L^1[p_H - p_0] + p_m(e_m^{s*})\right)\frac{e_m^{s*}}{p_m(e_m^{s*})}$$

$$= \bar{U}_m - \left(p_0 + \gamma_L^1[p_H - p_0]\right)\frac{e_m^{s*}}{p_m(e_m^{s*})}$$

By assumption, $\bar{U}_m \geq p_H \frac{e_m^{s*}}{p_m(e_m^{s*})}$. Therefore, $m_L^* > 0$, so this solution remains feasible when (LL_m) is imposed and is therefore a solution to the complete problem. Note that this solution matches that given in Table 3 when $\Delta m = \Delta m^*$.

Note: Given this solution, Case 1 applies when $(p_L - p_0)(\Delta x - \frac{e_m^{s*}}{p_m(e_m^{s*})}) < \bar{U} + \beta_H e_L$ or $\gamma_L^0 < \gamma_L^2$,

where $\gamma_L^0 = \frac{(p_L-p_0)(\Delta x-\frac{e_m^{s*}}{p_m(e_m^{s*})})-(\bar{U}+\beta_H e_L)}{(p_L-p_0)(\Delta x-\frac{e_m^{s*}}{p_m(e_m^{s*})})-(\bar{U}+\beta_L e_L)}$ and $\gamma_L^2 = \frac{(p_H-p_L)(\Delta x-\frac{e_m^{s*}}{p_m(e_m^{s*})})-\beta_H \Delta e}{(p_H-p_L)(\Delta x-\frac{e_m^{s*}}{p_m(e_m^{s*})})-\beta_L \Delta e}$.

It is possible that the firm might consider lowering Δm so that Case 2 applies instead. In that case, however, $\Delta m < \frac{e_m^{s*}}{p_m(e_m^{s*})}$, so the manager chooses to exert $e_m^s = 0$, which the firm does not prefer (by assumption).

Case 2: Otherwise $\left((p_L - p_0)(\Delta x - \Delta m) > \bar{U} + \beta_H e_L \text{ AND } \gamma_L^0 > \gamma_L^2\right)$

The firm chooses the manager's contract by solving the following:

$$\max_{m_L,\Delta m} E[\pi] = \int_0^{\gamma_L^2} E[\pi(e_H,e_H)]f(\gamma_L)\mathrm{d}\gamma_L + \int_{\gamma_L^2}^{\gamma_L^0} E[\pi(e_H,e_L)]f(\gamma_L)\mathrm{d}\gamma_L + \int_{\gamma_L^0}^{1} E[\pi(e_H,0)]f(\gamma_L)\mathrm{d}\gamma_L$$

$$\text{s.t. } m_L + \left(p_0 + \gamma_L^0[p_L - p_0] + \gamma_L^2[p_H - p_L] + p_m(e_m^s)\right)\Delta m - e_m^s \geq \bar{U}_m \qquad (\mathrm{IR_m})$$

$$\Delta m \geq \frac{e_m^{s*}}{p_m(e_m^{s*})} \qquad (\mathrm{IC_m})$$

$$m_L \geq 0 \qquad (\mathrm{LL_m})$$

With some substitutions, the objective function is:

$$\begin{aligned}
E[\pi] = &\int_0^{\gamma_L^2} \left[x_L - m_L + \left[p_H + p_m(e_m^{s*})\right](\Delta x - \Delta m) - (\bar{U} + \beta_H e_H)\right]\mathrm{d}\gamma_L + \\
&\int_{\gamma_L^2}^{\gamma_L^0} \left[x_L - m_L + \left[p_L + p_m(e_m^{s*})\right](\Delta x - \Delta m) - [\bar{U} + \beta_H e_L] + \gamma_L\left([p_H - p_L](\Delta x - \Delta m) - \beta_L \Delta e\right)\right]\mathrm{d}\gamma_L + \\
&\int_{\gamma_L^0}^{1} \left[x_L - m_L + \left[p_0 + p_m(e_m^{s*})\right](\Delta x - \Delta m) + \gamma_L\left([p_H - p_0](\Delta x - \Delta m) - [\bar{U} + \beta_L e_H]\right)\right]\mathrm{d}\gamma_L \\
&= \gamma_L^2\left[x_L - m_L + \left[p_H + p_m(e_m^{s*})\right](\Delta x - \Delta m) - (\bar{U} + \beta_H e_H)\right] + \\
&(\gamma_L^0 - \gamma_L^2)\left[x_L - m_L + \left[p_L + p_m(e_m^{s*})\right](\Delta x - \Delta m) - [\bar{U} + \beta_H e_L] + \frac{\gamma_L^0 + \gamma_L^2}{2}\left([p_H - p_L](\Delta x - \Delta m) - \beta_L \Delta e\right)\right] + \\
&(1 - \gamma_L^0)\left[x_L - m_L + \left[p_0 + p_m(e_m^{s*})\right](\Delta x - \Delta m) + \frac{1 + \gamma_L^0}{2}\left([p_H - p_0](\Delta x - \Delta m) - [\bar{U} + \beta_L e_H]\right)\right] \\
&= x_L - m_L + p_m(e_m^{s*})(\Delta x - \Delta m) + \gamma_L^2\left[p_H(\Delta x - \Delta m) - (\bar{U} + \beta_H e_H)\right] + \\
&\quad (\gamma_L^0 - \gamma_L^2)\left[p_L(\Delta x - \Delta m) - (\bar{U} + \beta_H e_L) + \frac{\gamma_L^0 + \gamma_L^2}{2}\left([p_H - p_L][\Delta x - \Delta m] - \beta_L \Delta e\right)\right] + \\
&\quad (1 - \gamma_L^0)\left[p_0(\Delta x - \Delta m) + \frac{1 + \gamma_L^0}{2}\left([p_H - p_0][\Delta x - \Delta m] - [\bar{U} + \beta_L e_H]\right)\right]
\end{aligned}$$

Removing fixed values, the firm's problem becomes

$$\max_{m_L,\Delta m} -\left(m_L + \left[p_0 + \gamma_L^0(p_L - p_0) + \gamma_L^2(p_H - p_L) + \frac{(\gamma_L^0)^2 - (\gamma_L^2)^2}{2}(p_H - p_L) + \frac{1-(\gamma_L^0)^2}{2}(p_H - p_0) + p_m(e_m^{s*})\right]\Delta m\right)$$

$$\text{s.t. } m_L + \left(p_0 + \gamma_L^0[p_L - p_0] + \gamma_L^2[p_H - p_L] + p_m(e_m^s)\right)\Delta m - e_m^s \geq \bar{U}_m \qquad (\text{IR}_\text{m})$$

$$\Delta m \geq \frac{e_m^{s*}}{p_m(e_m^{s*})} \qquad (\text{IC}_\text{m})$$

$$m_L \geq 0 \qquad (\text{LL}_\text{m})$$

Again, consider first the identical problem, but with the (LL_m) constraint removed. Then (IR_m) must bind, because otherwise the firm can decrease m_L and increase the objective function without violating any constraints.

$\therefore m_L = \bar{U}_m + e_m^{s*} - \left(p_0 + \gamma_L^0[p_L - p_0] + \gamma_L^2[p_H - p_L] + p_m(e_m^s)\right)\Delta m$

Then the objective function is

$$\max_{\Delta m} -\left(\bar{U}_m + e_m^{s*} + \left[\frac{(\gamma_L^0)^2 - (\gamma_L^2)^2}{2}(p_H - p_L) + \frac{1-(\gamma_L^0)^2}{2}(p_H - p_0)\right]\Delta m\right)$$

$$\iff \min_{\Delta m}\frac{1}{2}\left(\left[1-(\gamma_L^0)^2\right][p_H - p_0] + \left[(\gamma_L^0)^2 - (\gamma_L^2)^2\right][p_H - p_L]\right)\Delta m$$

$$\iff \min_{\Delta m}\left(p_H - p_0 - (\gamma_L^2)^2[p_H - p_L] - (\gamma_L^0)^2[p_L - p_0]\right)\Delta m$$

Since $\gamma_L^0 = 1 - \frac{\Delta\beta e_L}{(p_L - p_0)(\Delta x - \Delta m) - (\bar{U} + \beta_L e_L)}$ and $\gamma_L^2 = 1 - \frac{\Delta\beta\Delta e}{(p_H - p_L)(\Delta x - \Delta m) - \beta_L\Delta e}$ are each positive and decreasing in Δm, $\therefore \left(p_H - p_0 - (\gamma_L^2)^2[p_H - p_L] - (\gamma_L^0)^2[p_L - p_0]\right)\Delta m$ is increasing in Δm. Therefore, the firm prefers to minimize Δm. Again, this is driven by the fact that when $\gamma_L > \gamma_L^2$, the manager earns rent that is increasing in Δm.

$$\therefore \Delta m^* = \frac{e_m^{s*}}{p_m(e_m^{s*})}$$

$$\therefore m_L^* = \bar{U}_m + e_m^{s*} - \left(p_0 + \gamma_L^0[p_L - p_0] + \gamma_L^2[p_H - p_L] + p_m(e_m^s)\right)\frac{e_m^{s*}}{p_m(e_m^{s*})}$$

$$= \bar{U}_m - \left(p_0 + \gamma_L^0[p_L - p_0] + \gamma_L^2[p_H - p_L]\right)\frac{e_m^{s*}}{p_m(e_m^{s*})}$$

By assumption, $\bar{U}_m \geq p_H\frac{e_m^{s*}}{p_m(e_m^{s*})}$. Therefore, $m_L^* > 0$, so this solution remains feasible when (LL_m)

is imposed and is therefore a solution to the complete problem. Note again that this solution matches that given in Table 3 when $\Delta m = \Delta m^*$.

Note: Given this solution, Case 2 applies when $(p_L - p_0)(\Delta x - \frac{e_m^{s*}}{p_m(e_m^{s*})}) > \bar{U} + \beta_H e_L$ and $\gamma_L^0 > \gamma_L^2$, where $\gamma_L^0 = \frac{(p_L - p_0)(\Delta x - \frac{e_m^{s*}}{p_m(e_m^{s*})}) - (\bar{U} + \beta_H e_L)}{(p_L - p_0)(\Delta x - \frac{e_m^{s*}}{p_m(e_m^{s*})}) - (\bar{U} + \beta_L e_L)}$ and $\gamma_L^2 = \frac{(p_H - p_L)(\Delta x - \frac{e_m^{s*}}{p_m(e_m^{s*})}) - \beta_H \Delta e}{(p_H - p_L)(\Delta x - \frac{e_m^{s*}}{p_m(e_m^{s*})}) - \beta_L \Delta e}$.

Again, it is possible that the firm might consider raising Δm so that $(p_L - p_0)(\Delta x - \Delta m) > \bar{U} + \beta_H e_L$ and Case 1 applies instead. However, increasing Δm clearly makes the solution less attractive to the firm, both by increasing the manager's expected surplus and by limiting the firm's viable options for $(e^{\beta_L}, e^{\beta_H})$, so the firm cannot benefit by "forcing" Case 1.

Comparison with menu of contracts

Recall **Lemma 4:** Lemma 3 and Proposition 1 hold when "contract" is replaced with "menu of contracts".

Proof: Given a menu of contracts $\{(s_L^1, s_H^1), (s_L^2, s_H^2), ..., (s_L^n, s_H^n)\}$, the salesperson chooses the one that maximizes his expected utility, $E[U(s_L^i, s_H^i)] = s_L^i + [p(e) + p_m(e_m^s)]\Delta s^i - \beta e$, where e is his optimal effort given (s_L^i, s_H^i). If none of the contracts satisfy his (IR) constraint, then he exits. Again, since there are only two territory types, the menu need not contain more than two options. Thus, the firm chooses a menu $\{(s_L^{\beta_L}, s_H^{\beta_L}), (s_L^{\beta_H}, s_H^{\beta_H})\}$ to maximize its expected profit given its belief γ_L^F, where (s_L^β, s_H^β) is the contract intended to be chosen by the salesperson in a territory of type β.

As above, the manager prefers support effort $e_m^s = \begin{cases} 0 & \text{if } \Delta m < \frac{e_m^{s*}}{p_m(e_m^{s*})} \\ e_m^{s*} & \text{if } \Delta m \geq \frac{e_m^{s*}}{p_m(e_m^{s*})} \end{cases}$.

Extension of Lemma 3 (An efficient *menu of contracts* to induce $(e^{\beta_L}, e^{\beta_H})$ has the following properties: When the territory is hard, the salesperson receives no surplus; and when the territory is easy, the salesperson receives an expected utility surplus of $\Delta\beta e^{\beta_H}$.)

Case a: $e^{\beta_H} > 0$ The salesperson is intended to accept a contract in either territory type. The

firm's problem can be expressed as:

$$\max_{(s_L^{\beta_L},\Delta s^{\beta_L}),(s_L^{\beta_H},\Delta s^{\beta_H})} E[\pi] = \gamma_L^F \left[x_L - m_L - s_L^{\beta_L} + [p(e^{\beta_L}) + p_m(e_m^s)](\Delta x - \Delta m - \Delta s^{\beta_L})\right] +$$
$$[1-\gamma_L^F]\left[x_L - m_L - s_L^{\beta_H} + [p(e^{\beta_H}) + p_m(e_m^s)](\Delta x - \Delta m - \Delta s^{\beta_H})\right]$$

$$\text{s. t. } s_L^{\beta_L} + [p(e^{\beta_L}) + p_m(e_m^s)]\Delta s^{\beta_L} - \beta_L e^{\beta_L} \geq \bar{U} \qquad (\text{IR}^{\beta_L})$$

$$s_L^{\beta_H} + [p(e^{\beta_H}) + p_m(e_m^s)]\Delta s^{\beta_H} - \beta_H e^{\beta_H} \geq \bar{U} \qquad (\text{IR}^{\beta_H})$$

$$s_L^{\beta_L} + [p(e^{\beta_L}) + p_m(e_m^s)]\Delta s^{\beta_L} - \beta_L e^{\beta_L} \geq s_L^{\beta_L} + [p(e) + p_m(e_m^s)]\Delta s^{\beta_L} - \beta_L e \ \forall e \in \{e_L, e_H\} \qquad (\text{IC}^{\beta_L})$$

$$s_L^{\beta_H} + [p(e^{\beta_H}) + p_m(e_m^s)]\Delta s^{\beta_H} - \beta_H e^{\beta_H} \geq s_L^{\beta_H} + [p(e) + p_m(e_m^s)]\Delta s^{\beta_H} - \beta_H e \ \forall e \in \{e_L, e_H\} \qquad (\text{IC}^{\beta_H})$$

$$s_L^{\beta_L} + [p(e^{\beta_L}) + p_m(e_m^s)]\Delta s^{\beta_L} - \beta_L e^{\beta_L} \geq s_L^{\beta_H} + [p(e) + p_m(e_m^s)]\Delta s^{\beta_H} - \beta_L e \ \forall e \in \{e_L, e_H\} \qquad (\text{SS}^{\beta_L})$$

$$s_L^{\beta_H} + [p(e^{\beta_H}) + p_m(e_m^s)]\Delta s^{\beta_H} - \beta_H e^{\beta_H} \geq s_L^{\beta_L} + [p(e) + p_m(e_m^s)]\Delta s^{\beta_L} - \beta_H e \ \forall e \in \{e_L, e_H\} \qquad (\text{SS}^{\beta_H})$$

$$s_L^{\beta_L} \geq 0 \qquad (\text{LL}^{\beta_L})$$

$$s_L^{\beta_H} \geq 0 \qquad (\text{LL}^{\beta_H})$$

Once again, I begin with an adapted version of this problem, omitting the (LL) constraints, then show that the optimal solution can be achieved without violating those additional constraints, making it a solution to the complete problem.

Claim: (IR^{β_H}) binds.

Proof: At least one of the (IR) constraints must bind, because otherwise the firm can lower $s_L^{\beta_L}$ and $s_L^{\beta_H}$ equally, increasing the objective function without violating any constraints.

Suppose (IR^{β_L}) binds. Then (SS^{β_L}) implies that

$$\bar{U} \geq s_L^{\beta_H} + [p(e^{\beta_H}) + p_m(e_m^s)]\Delta s^{\beta_H} - \beta_L e^{\beta_H}$$
$$> s_L^{\beta_H} + [p(e^{\beta_H}) + p_m(e_m^s)]\Delta s^{\beta_H} - \beta_H e^{\beta_H}$$
$$\therefore \bar{U} > \bar{U} \qquad \text{by } (\text{IR}^{\beta_H})$$

This is a contradiction, so (IR^{β_H}) must bind. □

∴ When the territory is hard, the salesperson receives no surplus, as in Lemma 3.

Claim: When the territory is easy, the salesperson receives an expected utility surplus of $\Delta\beta e^{\beta_H}$.

Proof: Substituting (IR^{β_H}) into (SS^{β_L}) gives $s_L^{\beta_L} + [p(e^{\beta_L}) + p_m(e_m^s)]\Delta s^{\beta_L} - \beta_L e^{\beta_L} \geq \bar{U} + \Delta\beta e^{\beta_H}$. So ($IR^{\beta_L}$) has slack (i.e., the salesperson receives rent in an easy territory) of *at least* $\Delta\beta e^{\beta_H}$.

(SS^{β_L}) must bind for some $e \in \{e_L, e_H\}$, because otherwise the firm can lower $s_L^{\beta_L}$ and increase the objective function without violating any constraints.

$$\therefore\ s_L^{\beta_L} + [p(e^{\beta_L}) + p_m(e_m^s)]\Delta s^{\beta_L} - \beta_L e^{\beta_L} = s_L^{\beta_H} + p_m(e_m^s)\Delta s^{\beta_H} + \max\{p_L \Delta s^{\beta_H} - \beta_L e_L, p_H \Delta s^{\beta_H} - \beta_L e_H\}$$

Subcase a.i: $e^{\beta_H} = e_L$ In this case, the firm can set $\Delta s^{\beta_H} = 0$, because the salesperson is expected to exert the minimum effort. So, by (IR^{β_H}), $s_L^{\beta_H} = s_H^{\beta_H} = \bar{U} + \beta_H e_L$. From above, then,

$$s_L^{\beta_L} + [p(e^{\beta_L}) + p_m(e_m^s)]\Delta s^{\beta_L} - \beta_L e^{\beta_L} = \bar{U} + \beta_H e_L + \max\{-\beta_L e_L, -\beta_L e_H\}$$
$$\therefore\ s_L^{\beta_L} + [p(e^{\beta_L}) + p_m(e_m^s)]\Delta s^{\beta_L} - \beta_L e^{\beta_L} = \bar{U} + \Delta\beta e_L$$

So, under this menu, the salesperson receives rent of $\Delta\beta e^{\beta_H}$ if the territory is easy. Therefore, since an efficient menu of contracts for a given $(e^{\beta_L}, e^{\beta_H})$ minimizes the rent paid in each territory type, *any* efficient menu for (e^{β_L}, e_L) must offer rent of $\Delta\beta e^{\beta_H}$ if the territory is easy.

$s_L^{\beta_H} = \bar{U} + \beta_H e_L > 0$, so imposing ($LL^{\beta_H}$) does not affect this solution.

If $e^{\beta_L} = e_L$, the firm can set $\Delta s^{\beta_L} = 0$, so $s_L^{\beta_L} = \bar{U} + \beta_H e_L > 0$.

If $e^{\beta_L} = e_H$, the firm can set $\Delta s^{\beta_L} = \frac{\beta_L \Delta e}{p_H - p_L}$, so $s_L^{\beta_L} = \bar{U} + \beta_H e_L - \frac{\beta_L \Delta e}{p_H - p_L}[p_L + p_m(e_m^s)]$. By assumption, $\bar{U} \geq \frac{\beta_H}{p_H - p_L}(p_L e_H - p_H e_L + p_m(e_m^{s*})\Delta e)$, so

$$s_L^{\beta_L} \geq \frac{\beta_H}{p_H - p_L}[p_L e_H - p_H e_L + p_m(e_m^{s*})\Delta e] + \beta_H e_L - \frac{\beta_L \Delta e}{p_H - p_L}[p_L + p_m(e_m^s)]$$
$$\therefore \geq \frac{1}{p_H - p_L}(\beta_H[p_L e_H - p_H e_L + p_m(e_m^{s*})] + \beta_H e_L[p_H - p_L] - \beta_L \Delta e[p_L + p_m(e_m^s)])$$
$$\therefore \geq \frac{\Delta\beta p_L \Delta e}{p_H - p_L}$$
$$\therefore\ s_L^{\beta_L} > 0$$

$\therefore$ imposing (LL^{β_L}) does not prevent the firm from implementing this solution for either e^{β_L}.

Subcase a.ii: $e^{\beta_H} = e_H$ $\therefore\ e^{\beta_L} = e_H$, because $e^{\beta_L} \geq e^{\beta_H}$.

Suppose (SS^{β_L}) binds for $e = e_L$. Then, substituting in (IR^{β_H}) gives

$s_L^{\beta_L} + [p_H + p_m(e_m^s)]\Delta s^{\beta_L} - \beta_L e_H = \bar{U} - (p_H - p_L)\Delta s^{\beta_H} + \beta_H e_H - \beta_L e_L$

From (IC^{β_H}), $(p_H - p_L)\Delta s^{\beta_H} \geq \beta_H e_H - \beta_H e_L$, so

$$\begin{aligned} s_L^{\beta_L} + [p_H + p_m(e_m^s)]\Delta s^{\beta_L} - \beta_L e_H &\leq \bar{U} - (\beta_H e_H - \beta_H e_L) + \beta_H e_H - \beta_L e_L \\ &\leq \bar{U} + \Delta\beta e_L \\ \therefore s_L^{\beta_L} + [p_H + p_m(e_m^s)]\Delta s^{\beta_L} - \beta_L e_H &< \bar{U} + \Delta\beta e_H \end{aligned}$$

This contradicts the finding above that (IR^{β_L}) has slack of at least $\Delta\beta e^{\beta_H}$. Therefore, (SS^{β_L}) cannot bind for $e = e_L$, so it must bind for $e = e_H$. Substituting in (IR^{β_H}) gives

$$s_L^{\beta_L} + [p_H + p_m(e_m^s)]\Delta s^{\beta_L} - \beta_L e_H = \bar{U} + \Delta\beta e_H$$

So, the salesperson receives rent of $\Delta\beta e^{\beta_H}$ if the territory is easy.

Again, since an efficient menu of contracts for a given $(e^{\beta_L}, e^{\beta_H})$ minimizes the rent paid in each territory type, *any* efficient menu for (e_H, e_H) must offer rent of $\Delta\beta e^{\beta_H}$ if the territory is easy.

This can be achieved with $\Delta s = \frac{\beta_H \Delta e}{p_H - p_L}$ and $s_L = \bar{U} + \beta_H e_H - \frac{\beta_H \Delta e}{p_H - p_L}[p_H + p_m(e_m^s)]$ for *both* territory types (i.e., a single-contract menu).

By $\bar{U} \geq \frac{\beta_H}{p_H - p_L}(p_L e_H - p_H e_L + p_m(e_m^{s*})\Delta e)$,

$$\begin{aligned} s_L &\geq \frac{\beta_H}{p_H - p_L}(p_L e_H - p_H e_L + p_m(e_m^{s*})\Delta e) + \beta_H e_H - \frac{\beta_H \Delta e}{p_H - p_L}[p_H + p_m(e_m^s)] \\ \therefore &\geq \frac{\beta_H}{p_H - p_L}(p_L e_H - p_H e_L + p_m(e_m^{s*})\Delta e + e_H[p_H - p_L] - [p_H + p_m(e_m^s)]\Delta e) \\ \therefore s_L &\geq 0 \end{aligned}$$

∴ this solution can be achieved without violating either (LL) constraint. □

Therefore, Lemma 3 always holds in Case a.

Case b: $e^{\beta_H} = 0$ The firm's problem reduces to a standard principal-agent model, with the following well-established solution:

To induce $(e_L, 0)$, by (IR^{β_L}), the firm sets $s_L^{\beta_L} = s_H^{\beta_L} = \bar{U} + \beta_L e_L$. Under this contract, ($IR^{\beta_L}$)

binds, so the salesperson accepts and earns a surplus of 0 ($= \Delta\beta e^{\beta_H}$) if the territory is easy. Conversely, (IR^{β_H}) is not satisfied for any e^{β_H}, so he exits (and earns 0 surplus) if it is hard. Clearly, $s_L^{\beta_L} = \bar{U} + \beta_L e_L > 0$, so this solution does not violate the (LL) constraints.

To induce $(e_H, 0)$, the firm offers

$$(s_L, s_H) = \left(\bar{U} + \beta_L \left[e_H - [p_H + p_m(e_m^s)]\frac{\Delta e}{p_H - p_L}\right], \bar{U} + \beta_L \left[e_H + \left(1 - [p_H + p_m(e_m^s)]\right)\frac{\Delta e}{p_H - p_L}\right]\right)$$

$\therefore \Delta s = \frac{\beta_L \Delta e}{p_H - p_L}$ and $s_L + [p_H + p_m(e_m^s)]\Delta s - \beta_L e_H = \bar{U}$, so the salesperson accepts and chooses e_H, earning 0 surplus, if the territory is easy. If the territory is hard, then the salesperson's expected utility if he stays and exerts $e \in \{e_L, e_H\}$ is

$$\begin{aligned}
s_L + [p(e) + p_m(e_m^s)]\Delta s - \beta_H e &= \bar{U} + \beta_L \left[e_H - [p_H + p_m(e_m^s)]\frac{\Delta e}{p_H - p_L}\right] + [p(e) + p_m(e_m^s)]\frac{\beta_L \Delta e}{p_H - p_L} - \beta_H e \\
&= \bar{U} + \beta_L \left(e_H - [p_H - p(e)]\frac{\Delta e}{p_H - p_L}\right) - \beta_H e \\
&= \bar{U} + \beta_L e - \beta_H e \\
&= \bar{U} - \Delta\beta e \\
\therefore s_L + p(e)\Delta s - \beta_H e &< \bar{U}
\end{aligned}$$

Therefore, if the territory is hard, then the salesperson exits and earns 0 surplus.

By $\bar{U} \geq \frac{\beta_H}{p_H - p_L}(p_L e_H - p_H e_L + p_m(e_m^{s*})\Delta e) \geq 0$,

$$\begin{aligned}
s_L &\geq \frac{\beta_H}{p_H - p_L}(p_L e_H - p_H e_L + p_m(e_m^{s*})\Delta e) + \beta_L \left[e_H - [p_H + p_m(e_m^s)]\frac{\Delta e}{p_H - p_L}\right] \\
\therefore &\geq \frac{1}{p_H - p_L}(\beta_H(p_L e_H - p_H e_L + p_m(e_m^{s*})\Delta e) + \beta_L[(p_H - p_L)e_H - (p_H + p_m(e_m^s))\Delta e]) \\
\therefore &\geq \frac{\Delta\beta}{p_H - p_L}(p_L e_H - p_H e_L + p_m(e_m^{s*})\Delta e) \\
\therefore s_L &\geq 0
\end{aligned}$$

$\therefore$ this solution can be achieved without violating either (LL) constraint.

Therefore, Lemma 3 holds in all cases. □

The proof of Proposition 1 extends directly from above, by replacing "efficient contract" with

"efficient menu of contracts". □

When deciding whether to accept her contract, the manager anticipates the menu that the firm will offer the salesperson as well as her own support effort, so she accepts if and only if:

$$E[U_m|(e^{\beta_L}, e^{\beta_H}), e^s_m] = m_L + \left(p(e^{\beta_H}) + \gamma_L \left[p(e^{\beta_L}) - p(e^{\beta_H})\right] + p_m(e^s_m)\right) \Delta m - e^s_m \geq \bar{U}_m \quad \text{(W6)}$$

Therefore:

If $(e^{\beta_L}, e^{\beta_H}) = (e_H, 0)$, she accepts if and only if $m_L + (p_0 + \gamma_L [p_H - p_0] + p_m(e^s_m)) \Delta m \geq \bar{U} + e^s_m$.

If (e_H, e_L), she accepts if and only if $m_L + (p_L + \gamma_L [p_H - p_L] + p_m(e^s_m)) \Delta m \geq \bar{U} + e^s_m$.

If (e_H, e_H), she accepts if and only if $m_L + \left[p_H + p_m(e^s_m)\right] \Delta m \geq \bar{U} + e^s_m$.

Since the firm is not relying on the manager's information, there is no benefit in employing her if she chooses $e^s_m = 0$. Therefore, if the firm chooses to satisfy (IR_m), it always prefers to induce $e^s_m = e^{s*}_m$. Therefore, the firm must choose $\Delta m \geq \frac{e^{s*}_m}{p_m(e^{s*}_m)}$.

Therefore, the firm's expected profit if the manager accepts her contract is

$$\begin{aligned} E[\pi(e_H, e^{\beta_H})|e^{s*}_m] = {} & x_L - m_L + p_m(e^{s*}_m)(\Delta x - \Delta m) + \gamma^F_L \left[p_H(\Delta x - \Delta m) - (\bar{U} + \beta_L e_H + \Delta\beta e^{\beta_H})\right] + \\ & (1 - \gamma^F_L) \left[p(e^{\beta_H})(\Delta x - \Delta m) - \mathbb{1}_{e^{\beta_H} > 0}(\bar{U} + \beta_H e^{\beta_H})\right] \\ = {} & x_L - m_L + \left[p(e^{\beta_H}) + p_m(e^{s*}_m)\right] (\Delta x - \Delta m) + \gamma^F_L \left(\left[p_H - p(e^{\beta_H})\right] (\Delta x - \Delta m) - \left[\bar{U} + \beta_L e_H + \Delta\beta e^{\beta_H}\right]\right) - \\ & (1 - \gamma^F_L)\mathbb{1}_{e^{\beta_H} > 0}(\bar{U} + \beta_H e^{\beta_H}) \end{aligned}$$

If the manager exits instead, then the firm's expected profit is

$$\begin{aligned} E[\pi(e_H, e^{\beta_H})|\,\text{exit}] = {} & x_L + p(e^{\beta_H})\Delta x + \gamma^F_L \left(\left[p_H - p(e^{\beta_H})\right] \Delta x - \left[\bar{U} + \beta_L e_H + \Delta\beta e^{\beta_H}\right]\right) - \\ & (1 - \gamma^F_L)\mathbb{1}_{e^{\beta_H} > 0}(\bar{U} + \beta_H e^{\beta_H}) \end{aligned}$$

Suppose the firm designs the manager's contract such that
$m_L + \left(p(e^{\beta_H}) + \gamma^*_L \left[p_H - p(e^{\beta_H})\right] + p_m(e^{s*}_m)\right) \Delta m = \bar{U}_m + e^{s*}_m$ for some $\gamma^*_L \in [0, 1]$.

If $e^{\beta_H} < e_H$, then from Inequality W6, she accepts (and exerts e^{s*}_m) if and only if $\gamma_L \geq \gamma^*_L$. Otherwise (if $e^{\beta_H} = e_H$), she accepts for all γ_L.

Case a: $e^{\beta_H} < e_H$ The firm's expected profit is

$$
\begin{aligned}
E[\pi] &= \int_0^{\gamma_L^*} E[\pi(e_H, e^{\beta_H})|\,\text{exit}] f(\gamma_L)\mathrm{d}\gamma_L + \int_{\gamma_L^*}^1 E[\pi(e_H, e^{\beta_H})|e_m^{s*}] f(\gamma_L)\mathrm{d}\gamma_L \\
&= \int_0^{\gamma_L^*} \Bigg[x_L + p(e^{\beta_H})\Delta x - \mathbb{1}_{e^{\beta_H}>0}(\bar{U} + \beta_H e^{\beta_H}) + \gamma_L\Bigg(\left[p_H - p(e^{\beta_H})\right]\Delta x - \left[\bar{U} + \beta_L e_H + \Delta\beta e^{\beta_H}\right] + \\
&\quad \mathbb{1}_{e^{\beta_H}>0}(\bar{U} + \beta_H e^{\beta_H})\Bigg)\Bigg]\mathrm{d}\gamma_L + \int_{\gamma_L^*}^1 \Bigg[x_L - m_L + \left[p(e^{\beta_H}) + p_m(e_m^{s*})\right](\Delta x - \Delta m) - \mathbb{1}_{e^{\beta_H}>0}(\bar{U} + \beta_H e^{\beta_H}) + \\
&\quad \gamma_L\left(\left[p_H - p(e^{\beta_H})\right](\Delta x - \Delta m) - \left[\bar{U} + \beta_L e_H + \Delta\beta e^{\beta_H}\right] + \mathbb{1}_{e^{\beta_H}>0}(\bar{U} + \beta_H e^{\beta_H})\right)\Bigg]\mathrm{d}\gamma_L \\
&= \gamma_L^*\Bigg[x_L + p(e^{\beta_H})\Delta x - \mathbb{1}_{e^{\beta_H}>0}(\bar{U} + \beta_H e^{\beta_H}) + \frac{\gamma_L^*}{2}\Bigg(\left[p_H - p(e^{\beta_H})\right]\Delta x - \left[\bar{U} + \beta_L e_H + \Delta\beta e^{\beta_H}\right] + \\
&\quad \mathbb{1}_{e^{\beta_H}>0}(\bar{U} + \beta_H e^{\beta_H})\Bigg)\Bigg] + (1-\gamma_L^*)\Bigg[x_L - m_L + \left[p(e^{\beta_H}) + p_m(e_m^{s*})\right](\Delta x - \Delta m) - \mathbb{1}_{e^{\beta_H}>0}(\bar{U} + \beta_H e^{\beta_H}) + \\
&\quad \frac{1+\gamma_L^*}{2}\left(\left[p_H - p(e^{\beta_H})\right](\Delta x - \Delta m) - \left[\bar{U} + \beta_L e_H + \Delta\beta e^{\beta_H}\right] + \mathbb{1}_{e^{\beta_H}>0}(\bar{U} + \beta_H e^{\beta_H})\right)\Bigg] \\
&= x_L + p(e^{\beta_H})\Delta x - \mathbb{1}_{e^{\beta_H}>0}(\bar{U} + \beta_H e^{\beta_H}) + \frac{1}{2}\Bigg(\left[p_H - p(e^{\beta_H})\right]\Delta x - \left[\bar{U} + \beta_L e_H + \Delta\beta e^{\beta_H}\right] + \\
&\quad \mathbb{1}_{e^{\beta_H}>0}(\bar{U} + \beta_H e^{\beta_H})\Bigg) + (1-\gamma_L^*)\Bigg[p_m(e_m^{s*})\Delta x - m_L - \left[p(e^{\beta_H}) + p_m(e_m^{s*})\right]\Delta m - \frac{1+\gamma_L^*}{2}\left(p_H - p(e^{\beta_H})\right)\Delta m\Bigg]
\end{aligned}
$$

Substituting in $m_L = \bar{U}_m + e_m^{s*} - \left(p(e^{\beta_H}) + \gamma_L^*\left[p_H - p(e^{\beta_H})\right] + p_m(e_m^{s*})\right)\Delta m$:

$$
\begin{aligned}
E[\pi] = x_L + \frac{1}{2}\Bigg(&\left[p_H + p(e^{\beta_H})\right]\Delta x - \left[\bar{U} + \beta_L e_H + \Delta\beta e^{\beta_H}\right] - \mathbb{1}_{e^{\beta_H}>0}(\bar{U} + \beta_H e^{\beta_H})\Bigg) + \\
&(1-\gamma_L^*)\Bigg[p_m(e_m^{s*})\Delta x - [\bar{U}_m + e_m^{s*}] - \frac{1-\gamma_L^*}{2}\left(p_H - p(e^{\beta_H})\right)\Delta m\Bigg]
\end{aligned}
$$

Now, suppose that $\gamma_L^* < 1$. Then $E[\pi]$ is decreasing in Δm, so the firm sets $\Delta m^* = \frac{e_m^{s*}}{p_m(e_m^{s*})}$.

$$
\therefore \frac{\partial E[\pi]}{\partial \gamma_L^*} = \bar{U}_m + e_m^{s*} - p_m(e_m^{s*})\Delta x + (1-\gamma_L^*)\left(p_H - p(e^{\beta_H})\right)\frac{e_m^{s*}}{p_m(e_m^{s*})}
$$

By assumption, $p_m(e_m^{s*})\Delta x > e_m^{s*} + \bar{U}_m + (p_H - p_0)\frac{e_m^{s*}}{p_m(e_m^{s*})}$, so $\frac{\partial E[\pi]}{\partial \gamma_L^*} < 0$. Therefore, the firm chooses m_L^* such that $\gamma_L^* = 0$ (i.e., such that the manager accepts her contract for *any* belief).

$$
\begin{aligned}
\therefore m_L^* &= \bar{U}_m + e_m^{s*} - \left(p(e^{\beta_H}) + p_m(e_m^{s*})\right)\frac{e_m^{s*}}{p_m(e_m^{s*})} \\
&= \bar{U}_m - p(e^{\beta_H})\frac{e_m^{s*}}{p_m(e_m^{s*})}
\end{aligned}
$$

By assumption, $\bar{U}_m \geq p_H \frac{e_m^{s*}}{p_m(e_m^{s*})}$, so $m_L^* \geq 0$. Therefore, the manager's (LL) constraint is satisfied.

$$\therefore\ E[\pi] = x_L - (\bar{U}_m + e_m^{s*}) + \left[p(e^{\beta_H}) + p_m(e_m^{s*})\right]\Delta x + \\ \frac{1}{2}\left(\left[p_H - p(e^{\beta_H})\right]\left[\Delta x - \frac{e_m^{s*}}{p_m(e_m^{s*})}\right] - \left[\bar{U} + \beta_L e_H + \Delta\beta e^{\beta_H}\right] - \mathbb{1}_{e^{\beta_H}>0}(\bar{U} + \beta_H e^{\beta_H})\right)$$

For comparison, suppose $\gamma_L^* = 1$. Then

$$E[\pi] = x_L + \frac{1}{2}\left(\left[p_H + p(e^{\beta_H})\right]\Delta x - \left[\bar{U} + \beta_L e_H + \Delta\beta e^{\beta_H}\right] - \mathbb{1}_{e^{\beta_H}>0}(\bar{U} + \beta_H e^{\beta_H})\right)$$

Therefore, since $p_m(e_m^{s*})\Delta x > e_m^{s*} + \bar{U}_m + (p_H - p_0)\frac{e_m^{s*}}{p_m(e_m^{s*})}$, the firm prefers $\gamma_L^* = 0$.

Case b: $\boldsymbol{e^{\beta_H} = e_H}$ $\quad \therefore m_L = \bar{U}_m + e_m^{s*} - \left[p_H + p_m(e_m^{s*})\right]\Delta m$

$$\begin{aligned} \therefore\ E[\pi] &= x_L - m_L + \left[p_H + p_m(e_m^{s*})\right](\Delta x - \Delta m) - (\bar{U} + \beta_H e_H) \\ &= x_L - \left(\bar{U}_m + e_m^{s*} - \left[p_H + p_m(e_m^{s*})\right]\Delta m\right) + \left[p_H + p_m(e_m^{s*})\right](\Delta x - \Delta m) - (\bar{U} + \beta_H e_H) \\ &= x_L + \left[p_H + p_m(e_m^{s*})\right]\Delta x - (\bar{U}_m + e_m^{s*}) - (\bar{U} + \beta_H e_H) \end{aligned}$$

Thus, the firm is indifferent between values of $\Delta m \geq \frac{e_m^{s*}}{p_m(e_m^{s*})}$, subject to the manager's limited liability constraint.

Suppose $\Delta m^* = \frac{e_m^{s*}}{p_m(e_m^{s*})}$. Then $m_L^* = \bar{U}_m - p_H \frac{e_m^{s*}}{p_m(e_m^{s*})} > 0$, by assumption, so ($\text{LL}_\text{m}$) is satisfied. Therefore, this is a feasible solution.

For comparison, suppose the firm lets the manager exit (i.e., offers $m_L < \bar{U}_m + e_m^{s*} - \left[p_H + p_m(e_m^{s*})\right]\Delta m$). Then $E[\pi] = x_L + p_H\Delta x - (\bar{U} + \beta_H e_H)$. Since $p_m(e_m^{s*})\Delta x > \bar{U}_m + e_m^{s*} + (p_H - p_0)\frac{e_m^{s*}}{p_m(e_m^{s*})}$, this is never preferred over the solution above.

Note that the solutions above indicate the best possible contracts for the manager, *given* e^{β_H}. However, the choice of e^{β_H} is endogenous, and depends on Δm through the definitions of Cases 1 & 2 (as defined throughout the paper) and the values of γ_L^0, γ_L^1, and γ_L^2. Therefore, it is necessary to consider whether the firm might prefer to set Δm differently, in order to result in a different choice of e^{β_H}.

It is straightforward to show that it does not, with the following intuition: Since the firm prefers

that the manager exerts effort e_m^{s*} and the solutions above result in $\Delta m^* = \frac{e_m^{s*}}{p_m(e_m^{s*})}$, the firm can only consider *increasing* Δm. Doing so (weakly) decreases e^{β_H} and increases the rent paid to the manager, so there is no benefit to the firm.

Finally, recall **Proposition 3:** The request mechanism outperforms the menu of contracts when either: a) The best menu of contracts induces the salesperson to exit when his territory is hard; or b) The manager's incentive pay (Δm) is not too large. The relative performance of the request mechanism increases as the firm's decision threshold(s) approach its belief (γ_L^F) about the territory type.

Proof: Under both the request mechanism and the menu of contracts, the firm sets $\Delta m^* = \frac{e_m^{s*}}{p_m(e_m^{s*})}$, so we can once again consider two cases.

Case 1: $\boldsymbol{(p_L - p_0)(\Delta x - \frac{e_m^{s*}}{p_m(e_m^{s*})}) < \bar{U} + \beta_H e_L}$ ***OR*** $\boldsymbol{\gamma_L^0 < \gamma_L^2}$ The firm prefers to induce $(e^{\beta_L}, e^{\beta_H}) = (e_H, e_H)$ if and only if $\gamma_L < \gamma_L^1 = \frac{(p_H - p_0)\left(\Delta x - \frac{e_m^{s*}}{p_m(e_m^{s*})}\right) - (\bar{U} + \beta_H e_H)}{(p_H - p_0)\left(\Delta x - \frac{e_m^{s*}}{p_m(e_m^{s*})}\right) - (\bar{U} + \beta_L e_H)}$ and $(e_H, 0)$ otherwise.

Under the request mechanism, $m_L^* = \bar{U}_m - \left(p_0 + \gamma_L^1 [p_H - p_0]\right) \frac{e_m^{s*}}{p_m(e_m^{s*})}$ and $\Delta m^* = \frac{e_m^{s*}}{p_m(e_m^{s*})}$, so from Step 1 above

$$E[\pi |\, \text{request}] = x_L - (\bar{U}_m + e_m^{s*}) + \left[p_0 + p_m(e_m^{s*})\right] \Delta x + \gamma_L^1 \left[(p_H - p_0)\Delta x - (\bar{U} + \beta_H e_H)\right] +$$
$$(1 - \gamma_L^1)\frac{1 + \gamma_L^1}{2} \left([p_H - p_0]\,[\Delta x - \frac{e_m^{s*}}{p_m(e_m^{s*})}] - [\bar{U} + \beta_L e_H]\right)$$

Under the menu of contracts, the firm chooses based on its own belief, $\gamma_L^F = 0.5$. From above, its expected profits are:

$$E[\pi |\, \text{menu}, \gamma_L^1 < 0.5] = x_L - (\bar{U}_m + e_m^{s*}) + \left[p_0 + p_m(e_m^{s*})\right] \Delta x + \frac{1}{2}\left([p_H - p_0]\left[\Delta x - \frac{e_m^{s*}}{p_m(e_m^{s*})}\right] - \left[\bar{U} + \beta_L e_H\right]\right)$$

$$E[\pi |\, \text{menu}, \gamma_L^1 > 0.5] = x_L + \left(p_H + p_m(e_m^{s*})\right) \Delta x - (\bar{U} + \beta_H e_H) - (\bar{U}_m + e_m^{s*})$$

Therefore, when $\gamma_L^1 < 0.5$:

$$E[\pi|\,\text{req}]{-}E[\pi|\,\text{menu},\gamma_L^1 < 0.5] =$$

$$\gamma_L^1 \left((p_H - p_0)\Delta x - (\bar{U} + \beta_H e_H) - \frac{\gamma_L^1}{2}\left[(p_H - p_0)(\Delta x - \frac{e_m^{s*}}{p_m(e_m^{s*})}) - (\bar{U} + \beta_L e_H) \right] \right)$$

$$= \gamma_L^1 \left[(p_H - p_0)(\Delta x - \frac{e_m^{s*}}{p_m(e_m^{s*})}) - (\bar{U} + \beta_L e_H) \right] \left(\gamma_L^1 - \frac{\gamma_L^1}{2} \right) + \gamma_L^1 (p_H - p_0)\frac{e_m^{s*}}{p_m(e_m^{s*})}$$

$$\therefore\ E[\pi|\,\text{req}]{-}E[\pi|\,\text{menu},\gamma_L^1 < 0.5] = \gamma_L^1 \left(\frac{\gamma_L^1}{2}\left[(p_H - p_0)(\Delta x - \frac{e_m^{s*}}{p_m(e_m^{s*})}) - (\bar{U} + \beta_L e_H) \right] + (p_H - p_0)\frac{e_m^{s*}}{p_m(e_m^{s*})} \right)$$

As shown in the proof of Proposition 1 (Appendix B), $(p_H - p_0)(\Delta x - \Delta m) > \bar{U} + \beta_H e_H > \bar{U} + \beta_L e_H$. Therefore, $E[\pi|\,\text{req}] > E[\pi|\,\text{menu}, \gamma_L^1 < 0.5]$, so the firm prefers the request mechanism in Case 1 when its best menu of contracts induces the salesperson to exit if his territory is hard.

Next, consider $\gamma_L^1 > 0.5$:

$$E[\pi|\,\text{req}]{-}E[\pi|\,\text{menu},\gamma_L^1 > 0.5] =$$

$$(1 - \gamma_L^1)\left[\frac{1 + \gamma_L^1}{2}\left([p_H - p_0]\,[\Delta x - \frac{e_m^{s*}}{p_m(e_m^{s*})}] - [\bar{U} + \beta_L e_H] \right) - \left([p_H - p_0]\Delta x - [\bar{U} + \beta_H e_H] \right) \right]$$

$$= (1 - \gamma_L^1)\left(\left[(p_H - p_0)(\Delta x - \frac{e_m^{s*}}{p_m(e_m^{s*})}) - (\bar{U} + \beta_L e_H) \right] \left[\frac{1 + \gamma_L^1}{2} - \gamma_L^1 \right] - (p_H - p_0)\frac{e_m^{s*}}{p_m(e_m^{s*})} \right)$$

$$E[\pi|\,\text{req}]{-}E[\pi|\,\text{menu},\gamma_L^1 > 0.5] = (1 - \gamma_L^1)\left(\frac{\Delta\beta e_H}{2} - (p_H - p_0)\frac{e_m^{s*}}{p_m(e_m^{s*})} \right)$$

This is positive if and only if: $\frac{e_m^{s*}}{p_m(e_m^{s*})} < \frac{\Delta\beta e_H}{2(p_H - p_0)}$

Therefore, when the firm's best menu of contracts does *not* induce the salesperson to exit in Case 1, the request mechanism is preferred under the same condition as in the main model (from Table 4).

Case 2: Otherwise $\boldsymbol{\left((p_L - p_0)(\Delta x - \frac{e_m^{s*}}{p_m(e_m^{s*})}) > \bar{U} + \beta_H e_L \textit{ AND } \gamma_L^0 > \gamma_L^2 \right)}$ The firm considers all three of $(e^{\beta_L}, e^{\beta_H}) = (e_H, 0), (e_H, e_L)$ and (e_H, e_H), choosing as follows:

$$\begin{cases} (e_H, 0) & \text{if } \gamma_L^0 < \gamma_L \\ (e_H, e_L) & \text{if } \gamma_L \in (\gamma_L^2, \gamma_L^0) \\ (e_H, e_H) & \text{if } \gamma_L < \gamma_L \end{cases}$$

where $\gamma_L^0 = \frac{(p_L-p_0)(\Delta x-\frac{e_m^{s*}}{p_m(e_m^{s*})})-(\bar{U}+\beta_H e_L)}{(p_L-p_0)(\Delta x-\frac{e_m^{s*}}{p_m(e_m^{s*})})-(\bar{U}+\beta_L e_L)}$, $\gamma_L^1 = \frac{(p_H-p_0)(\Delta x-\frac{e_m^{s*}}{p_m(e_m^{s*})})-(\bar{U}+\beta_H e_H)}{(p_H-p_0)(\Delta x-\frac{e_m^{s*}}{p_m(e_m^{s*})})-(\bar{U}+\beta_L e_H)}$, and $\gamma_L^2 = \frac{(p_H-p_L)(\Delta x-\frac{e_m^{s*}}{p_m(e_m^{s*})})-\beta_H\Delta e}{(p_H-p_L)(\Delta x-\frac{e_m^{s*}}{p_m(e_m^{s*})})-\beta_L\Delta e}$.

Under the request mechanism, $m_L^* = \bar{U}_m - \left(p_0 + \gamma_L^0[p_L - p_0] + \gamma_L^2[p_H - p_L]\right)\frac{e_m^{s*}}{p_m(e_m^{s*})}$ and $\Delta m^* = \frac{e_m^{s*}}{p_m(e_m^{s*})}$, so from Step 1 above

$$E[\pi|\,\text{request}] = x_L-(\bar{U}_m+e_m^{s*})+[p_0+p_m(e_m^{s*})]\Delta x+\gamma_L^2\left[(p_H-p_L)\Delta x-\beta_H\Delta e\right]+\gamma_L^0\left[(p_L-p_0)\Delta x-(\bar{U}+\beta_H e_L)\right]$$
$$+\frac{(\gamma_L^0)^2-(\gamma_L^2)^2}{2}\left([p_H-p_L]\,[\Delta x-\frac{e_m^{s*}}{p_m(e_m^{s*})}]-\beta_L\Delta e\right)+\frac{1-(\gamma_L^0)^2}{2}\left([p_H-p_0]\,[\Delta x-\frac{e_m^{s*}}{p_m(e_m^{s*})}]-[\bar{U}+\beta_L e_H]\right)$$

Under the menu of contracts, the firm's expected profits are:

$$E[\pi|\,\text{menu}, \gamma_L^0 < 0.5] = x_L+\left[p_0+p_m(e_m^{s*})\right]\Delta x-(\bar{U}_m+e_m^{s*})+\frac{1}{2}\left([p_H-p_0]\left[\Delta x-\frac{e_m^{s*}}{p_m(e_m^{s*})}\right]-\left[\bar{U}+\beta_L e_H\right]\right)$$

$$E[\pi|\,\text{menu}, 0.5 \in (\gamma_L^2,\gamma_L^0)] = x_L+\left[p_L+p_m(e_m^{s*})\right]\Delta x-(\bar{U}_m+e_m^{s*})-(\bar{U}+\beta_H e_L)+$$
$$\frac{1}{2}\left([p_H-p_L]\left[\Delta x-\frac{e_m^{s*}}{p_m(e_m^{s*})}\right]-\beta_L\Delta e\right)$$

$$E[\pi|\,\text{menu}, \gamma_L^2 > 0.5] = x_L+\left(p_H+p_m(e_m^{s*})\right)\Delta x-(\bar{U}_m+e_m^{s*})-(\bar{U}+\beta_H e_H)$$

Therefore, when $\gamma_L^0 < 0.5$:

$$E[\pi|\,\text{req}]-E[\pi|\,\text{menu}, \gamma_L^0 < 0.5] = \gamma_L^2\left[(p_H-p_L)\Delta x-\beta_H\Delta e\right]+\gamma_L^0\left[(p_L-p_0)\Delta x-(\bar{U}+\beta_H e_L)\right]+$$
$$\frac{(\gamma_L^0)^2-(\gamma_L^2)^2}{2}\left([p_H-p_L]\,[\Delta x-\frac{e_m^{s*}}{p_m(e_m^{s*})}]-\beta_L\Delta e\right)-\frac{(\gamma_L^0)^2}{2}\left([p_H-p_0]\,[\Delta x-\frac{e_m^{s*}}{p_m(e_m^{s*})}]-[\bar{U}+\beta_L e_H]\right)$$
$$=\left[\gamma_L^0(p_L-p_0)+\gamma_L^2(p_H-p_L)\right]\frac{e_m^{s*}}{p_m(e_m^{s*})}+\frac{(\gamma_L^0)^2}{2}\left[(p_L-p_0)(\Delta x-\frac{e_m^{s*}}{p_m(e_m^{s*})})-(\bar{U}+\beta_L e_L)\right]+$$
$$\frac{(\gamma_L^2)^2}{2}\left[(p_H-p_L)(\Delta x-\frac{e_m^{s*}}{p_m(e_m^{s*})})-\beta_L\Delta e\right]$$

The last step is taken from the detailed proof of Proposition 3 (Web Appendix F), with $\Delta m = \frac{e_m^{s*}}{p_m(e_m^{s*})}$.

From the definition of Case 2, $(p_L - p_0)(\Delta x - \frac{e_m^{s*}}{p_m(e_m^{s*})}) > \bar{U} + \beta_H e_L > \bar{U} + \beta_L e_L$ and from the proof of Proposition 1 (Appendix B), $(p_H - p_L)(\Delta x - \frac{e_m^{s*}}{p_m(e_m^{s*})}) > \beta_H\Delta e > \beta_L\Delta e$. Therefore, $E[\pi|\,\text{req}] - E[\pi|\,\text{menu}, \gamma_L^0 < 0.5] > 0$, so the firm prefers the request mechanism in Case 2 when its best menu of contracts induces the salesperson to exit if his territory is hard.

Next, consider $\gamma_L^2 < 0.5 < \gamma_L^0$:

$$E[\pi|\,\text{req}] - E[\pi|\,\text{menu}, \gamma_L^2 < 0.5 < \gamma_L^0] = -(1-\gamma_L^0)\left[(p_L - p_0)\Delta x - (\bar{U} + \beta_H e_L)\right] + \gamma_L^2\left[(p_H - p_L)\Delta x - \beta_H \Delta e\right] - \frac{1}{2}\left[(p_H - p_L)(\Delta x - \frac{e_m^{s*}}{p_m(e_m^{s*})}) - \beta_L \Delta e\right] + (1-\gamma_L^2)\frac{1+\gamma_L^2}{2}\left[(p_H - p_L)(\Delta x - \frac{e_m^{s*}}{p_m(e_m^{s*})}) - \beta_L \Delta e\right] + (1-\gamma_L^0)\frac{1+\gamma_L^0}{2}\left[(p_L - p_0)(\Delta x - \frac{e_m^{s*}}{p_m(e_m^{s*})}) - (\bar{U} + \beta_L e_L)\right]$$

$$= \frac{1}{2}\left[(p_H - p_L)\Delta x - \beta_H \Delta e\right] + \frac{1-\gamma_L^0}{2}\Delta\beta e_L - \frac{\gamma_L^2}{2}\Delta\beta\Delta e - \left[\left(\frac{1}{2} - \gamma_L^2\right)(p_H - p_L) + (1-\gamma_L^0)(p_L - p_0)\right]\frac{e_m^{s*}}{p_m(e_m^{s*})}$$

Again, the last step is taken from the detailed proof of Proposition 3 (Web Appendix F), with $\Delta m = \frac{e_m^{s*}}{p_m(e_m^{s*})}$.

$\therefore$ the request mechanism is preferred if and only if

$$\frac{e_m^{s*}}{p_m(e_m^{s*})} < \frac{(p_H - p_L)\Delta x - \beta_H \Delta e + (1-\gamma_L^0)\Delta\beta e_L - \gamma_L^2 \Delta\beta\Delta e}{2\left[\left(\frac{1}{2} - \gamma_L^2\right)(p_H - p_L) + (1-\gamma_L^0)(p_L - p_0)\right]}$$

Therefore, when the firm's best menu of contracts induces (e_H, e_L), the request mechanism is preferred under the same condition as in the main model (from Table 4).

Finally, consider $\gamma_L^2 > 0.5$:

$$E[\pi|\,\text{req}] - E[\pi|\,\text{menu}, \gamma_L^2 > 0.5] = -(1-\gamma_L^0)\left[(p_L - p_0)(\Delta x - \frac{e_m^{s*}}{p_m(e_m^{s*})}) - (\bar{U} + \beta_H e_L)\right] - (1-\gamma_L^2)\left[(p_H - p_L)(\Delta x - \frac{e_m^{s*}}{p_m(e_m^{s*})}) - \beta_H \Delta e\right] - \left[(1-\gamma_L^0)(p_L - p_0) + (1-\gamma_L^2)(p_H - p_L)\right]\frac{e_m^{s*}}{p_m(e_m^{s*})} + \frac{1-(\gamma_L^2)^2}{2}\left[(p_H - p_L)(\Delta x - \frac{e_m^{s*}}{p_m(e_m^{s*})}) - \beta_L \Delta e\right] + \frac{1-(\gamma_L^0)^2}{2}\left[(p_L - p_0)(\Delta x - \frac{e_m^{s*}}{p_m(e_m^{s*})}) - (\bar{U} + \beta_L e_L)\right]$$

$$= \frac{1}{2}\left[(1-\gamma_L^0)\Delta\beta e_L + (1-\gamma_L^2)\Delta\beta\Delta e\right] - \left[(1-\gamma_L^0)(p_L - p_0) + (1-\gamma_L^2)(p_H - p_L)\right]\frac{e_m^{s*}}{p_m(e_m^{s*})}$$

Once again, the last step is taken from the detailed proof of Proposition 3 (Web Appendix F), with $\Delta m = \frac{e_m^{s*}}{p_m(e_m^{s*})}$.

$\therefore$ the request mechanism is preferred if and only if

$$\frac{e_m^{s*}}{p_m(e_m^{s*})} < \frac{(1-\gamma_L^0)\Delta\beta e_L + (1-\gamma_L^2)\Delta\beta\Delta e}{2\left[(1-\gamma_L^0)(p_L - p_0) + (1-\gamma_L^2)(p_H - p_L)\right]}$$

Thus, when the firm's best menu of contracts induces (e_H, e_H), the request mechanism is preferred under the same condition as in the main model (from Table 4). □

WEB APPENDIX H: PROOF OF PROPOSITION 5

The manager chooses to acquire information if and only if her expected utility with that information (including the cost of acquiring it) exceeds her expected utility without it.

As in the proof of Proposition 2 (Appendix C), there are two cases to consider, based on whether the firm can eliminate $(e^{\beta_L}, e^{\beta_H}) = (e_H, e_L)$ as an option.

Case 1: $(p_L - p_0)(\Delta x - \Delta m) < \bar{U} + \beta_H e_L$ OR $\gamma_L^0 < \gamma_L^2$

The firm prefers $e^{\beta_H} = e_H$ if and only if $\gamma_L < \gamma_L^1 = \frac{(p_H - p_0)(\Delta x - \Delta m) - (\bar{U} + \beta_H e_H)}{(p_H - p_0)(\Delta x - \Delta m) - (\bar{U} + \beta_L e_H)}$ and $e^{\beta_H} = 0$ otherwise. The firm sets baseline constraints allowing $(e_H, 0)$ and requirements such that the manager must incur effort cost $e_m^{r*} = (1 - \gamma_L^1)(p_H - p_0)\Delta m$ to request constraints that allow (e_H, e_H).

From Step 2, if the manager does *not* acquire additional information, then

$$E[U_m | \gamma_L > \gamma_L^1] = m_L + p_H \Delta m - (1 - \gamma_L)(p_H - p_0)\Delta m \tag{W7}$$

and

$$E[U_m | \gamma_L < \gamma_L^1] = m_L + p_H \Delta m - (1 - \gamma_L^1)(p_H - p_0)\Delta m \tag{W8}$$

If the manager *does* acquire additional information, at cost C, then she learns with certainty that the territory is either easy or hard, updating her belief to $\gamma_L' = 0$ or 1. Since her current belief is accurate, $\gamma_L' = 1$ with probability γ_L and $\gamma_L' = 0$ with probability $1 - \gamma_L$.

Thus, her expected utility with additional information is:

$$\begin{aligned} E[U_m | \text{ info}] &= \gamma_L E[U_m | \gamma_L' = 1] + (1 - \gamma_L) E[U_m | \gamma_L' = 0] - C \\ &= \gamma_L (m_L + p_H \Delta m) + (1 - \gamma_L) \left(m_L + p_H \Delta m - [1 - \gamma_L^1][p_H - p_0]\Delta m \right) - C \end{aligned}$$

$$\therefore E[U_m | \text{ info}] = m_L + p_H \Delta m - (1 - \gamma_L)(1 - \gamma_L^1)(p_H - p_0)\Delta m - C \tag{W9}$$

Subtracting Equations W7 and W8 from Equation W9 gives:

$$E[U_m|\,\text{info}] - E[U_m|\gamma_L > \gamma_L^1] = \gamma_L^1(1-\gamma_L)(p_H - p_0)\Delta m - C$$

$$E[U_m|\,\text{info}] - E[U_m|\gamma_L < \gamma_L^1] = \gamma_L(1-\gamma_L^1)(p_H - p_0)\Delta m - C$$

Each of these is maximized as $\gamma_L \rightarrow \gamma_L^1$. Therefore, the manager will acquire information when γ_L is sufficiently close to γ_L^1, if C is not too high. (Specifically, if $C < \gamma_L^1(1-\gamma_L^1)(p_H - p_0)\Delta m$.)

Case 2: Otherwise $\big((p_L - p_0)(\Delta x - \Delta m) > \bar{U} + \beta_H e_L$ AND $\gamma_L^0 > \gamma_L^2\big)$

The firm begins with baseline constraints allowing $(e_H, 0)$ and sets sequential requirements such that the manager must incur effort cost $e_m^{rL} = (1-\gamma_L^0)(p_L - p_0)\Delta m$ to request constraints that allow (e_H, e_L), followed by $e_m^{rH} = (1-\gamma_L^2)(p_H - p_L)\Delta m$ to allow (e_H, e_H), where $\gamma_L^0 = \frac{(p_L - p_0)(\Delta x - \Delta m) - (\bar{U} + \beta_H e_L)}{(p_L - p_0)(\Delta x - \Delta m) - (\bar{U} + \beta_L e_L)}$ and $\gamma_L^2 = \frac{(p_H - p_L)(\Delta x - \Delta m) - \beta_H \Delta e}{(p_H - p_L)(\Delta x - \Delta m) - \beta_L \Delta e}$.

If the manager does *not* acquire additional information, then her expected utility is as follows:

$$E[U_m|\gamma_L > \gamma_L^0] = m_L + p_H\Delta m - (1-\gamma_L)(p_H - p_0)\Delta m \tag{W10}$$

$$E[U_m|\gamma_L \in (\gamma_L^2, \gamma_L^0)] = m_L + p_H\Delta m - [(1-\gamma_L)(p_H - p_L) + (1-\gamma_L^0)(p_L - p_0)]\Delta m \tag{W11}$$

and
$$E[U_m|\gamma_L < \gamma_L^2] = m_L + p_H\Delta m - [(1-\gamma_L^0)(p_L - p_0) + (1-\gamma_L^2)(p_H - p_L)]\Delta m \tag{W12}$$

If the manager *does* acquire additional information, her expected utility is:

$$\begin{aligned}
E[U_m|\,\text{info}] &= \gamma_L E[U_m|\gamma_L' = 1] + (1-\gamma_L)E[U_m|\gamma_L' = 0] - C \\
&= \gamma_L(m_L + p_H\Delta m) + (1-\gamma_L)\Big(m_L + p_H\Delta m - \big[(1-\gamma_L^0)(p_L - p_0) + (1-\gamma_L^2)(p_H - p_L)\big]\,\Delta m\Big) - C \\
&= m_L + p_H\Delta m - (1-\gamma_L)\big[(1-\gamma_L^0)(p_L - p_0) + (1-\gamma_L^2)(p_H - p_L)\big]\,\Delta m - C \qquad \text{(W13)}
\end{aligned}$$

Thus, the manager's net utility from acquiring information is determined by subtracting Equation

W10, W11, or W12 from Equation W13:

$$E[U_m|\,\text{info}] - E[U_m|\gamma_L > \gamma_L^0] = (1-\gamma_L)[\gamma_L^0(p_L - p_0) + \gamma_L^2(p_H - p_L)]\Delta m - C$$

$$E[U_m|\,\text{info}] - E[U_m|\gamma_L \in (\gamma_L^2, \gamma_L^0)] = [\gamma_L(1-\gamma_L^0)(p_L - p_0) + \gamma_L^2(1-\gamma_L)(p_H - p_L)]\Delta m - C$$

$$E[U_m|\,\text{info}] - E[U_m|\gamma_L < \gamma_L^2] = \gamma_L[(1-\gamma_L^0)(p_L - p_0) + (1-\gamma_L^2)(p_H - p_L)]\Delta m - C$$

From the last of these, it is straightforward to see that her net utility increases with γ_L for $\gamma_L < \gamma_L^2$. Similarly, from the first, her net utility decreases with γ_L for $\gamma_L > \gamma_L^0$.

Between γ_L^2 and γ_L^0:

$\frac{\partial}{\partial \gamma_L}\left(E[U_m|\,\text{info}] - E[U_m|\gamma_L \in (\gamma_L^2, \gamma_L^0)]\right) = [(1-\gamma_L^0)(p_L - p_0) - \gamma_L^2(p_H - p_L)]\Delta m$

If this is positive, the manager's net utility is maximized as $\gamma_L \to \gamma_L^0$. Otherwise, it is maximized as $\gamma_L \to \gamma_L^2$.

Therefore, the manager will acquire additional information when γ_L is sufficiently close to γ_L^0 and/or γ_L^2, if C is not too high. $\square$
